# Na^+^ and/or Cl^−^ Toxicities Determine Salt Sensitivity in Soybean (*Glycine max* (L.) *Merr.*), Mungbean (*Vigna radiata* (L.) R. Wilczek), Cowpea (*Vigna unguiculata* (L.) *Walp.*), and Common Bean (*Phaseolus vulgaris* L.)

**DOI:** 10.3390/ijms22041909

**Published:** 2021-02-14

**Authors:** Ly Thi Thanh Le, Lukasz Kotula, Kadambot H. M. Siddique, Timothy D. Colmer

**Affiliations:** 1 School of Agriculture and Environment, The University of Western Australia, Perth, WA 6009, Australia; lukasz.kotula@uwa.edu.au (L.K.); kadambot.siddique@uwa.edu.au (K.H.M.S.); timothy.colmer@uwa.edu.au (T.D.C.); 2 The UWA Institute of Agriculture, The University of Western Australia, Perth, WA 6009, Australia; 3 Field Crops Research Institute, Gialoc, Haiduong, Vietnam

**Keywords:** salinity stress, specific ion stress, osmotic stress, growth responses, photosynthesis responses, ion “exclusion”, tissue tolerance of Na^+^, tissue tolerance of Cl^−^

## Abstract

Grain legumes are important crops, but they are salt sensitive. This research dissected the responses of four (sub)tropical grain legumes to ionic components (Na^+^ and/or Cl^−^) of salt stress. Soybean, mungbean, cowpea, and common bean were subjected to NaCl, Na^+^ salts (without Cl^−^), Cl^−^ salts (without Na^+^), and a “high cation” negative control for 57 days. Growth, leaf gas exchange, and tissue ion concentrations were assessed at different growing stages. For soybean, NaCl and Na^+^ salts impaired seed dry mass (30% of control), more so than Cl^−^ salts (60% of control). All treatments impaired mungbean growth, with NaCl and Cl^−^ salt treatments affecting seed dry mass the most (2% of control). For cowpea, NaCl had the greatest adverse impact on seed dry mass (20% of control), while Na^+^ salts and Cl^−^ salts had similar intermediate effects (~45% of control). For common bean, NaCl had the greatest adverse effect on seed dry mass (4% of control), while Na^+^ salts and Cl^−^ salts impaired seed dry mass to a lesser extent (~45% of control). NaCl and Na^+^ salts (without Cl^−^) affected the photosynthesis (*P_n_*) of soybean more than Cl^−^ salts (without Na^+^) (50% of control), while the reverse was true for mungbean. Na^+^ salts (without Cl^−^), Cl^−^ salts (without Na^+^), and NaCl had similar adverse effects on *P_n_* of cowpea and common bean (~70% of control). In conclusion, salt sensitivity is predominantly determined by Na^+^ toxicity in soybean, Cl^−^ toxicity in mungbean, and both Na^+^ and Cl^−^ toxicity in cowpea and common bean.

## 1. Introduction

Grain legumes (also known as pulses or food legumes) are important food sources for humans and animals, as the seeds are high in protein, dietary fibre, and essential amino acids, and have a low glycemic index [1]. Grain legume seeds contain up to 40% protein compared with only 6−10% in cereal grains, as well as high lysine and methionine contents [2]. In some species, the seeds contain oils with a low cholesterol constituent [3]. Grain legumes play an important role in the nitrogen budget in many agricultural systems owing to their ability to fix atmospheric nitrogen (*N*), which, when released from residues, can benefit a subsequent non-legume crop or, in some farming systems, when used as green manures [4]. It has been estimated that legumes provide soil with 100–200 kg *N* ha^−1^ per crop through a symbiotic relationship with *N*-fixing rhizobia bacteria [5]. The benefits of legumes also include a break in disease, weed, and pest incidence in cropping systems with cereals [6].

Grain legumes are grown in tropical and subtropical regions, while others are grown in temperate regions and semi-arid areas [7]. Soybean (*Glycine max*), mungbean (*Vigna radiata)*, cowpea (*Vigna unguiculata*), and common bean (*Phaseolus vulgaris*) are important crops in many tropical countries, but in some regions of the semi-arid tropics, soil salinity can limit grain legume productivity [8]. In general, salinity stress has a negative effect on growth, nodulation, quality and quantity of seed, and final yield in the grain legumes mentioned above. As such, salinity stress affects reproductive attributes, such as flower number, pod development, seed number, and seed size [9]. In addition, salinity inhibits plant growth due to low external water potentials, ion toxicity, and ion imbalance. Excess ions result in an “osmotic effect” on plant water relations, and specific ions can have individual or synergistic effects on ionic relations/toxicity and nutritional imbalances [10,11,12]. Ion toxicity and cation imbalance are the most destructive for grain legumes, as these species are sensitive to salinity levels with only modest levels of osmotic potential [13,14,15]. Salt-stressed plants accumulate considerable Na^+^ and/or Cl^−^ in tissues, which decreases leaf area and photosynthesis, reduces the supply of assimilates to reproductive organs, and reduces final grain yield [16,17]. The tolerance mechanisms for salt stress include maintenance of ion homeostasis (maintenance of K^+^, “exclusion” of Na^+^) [18], adjustment of internal osmotic potential, and some metabolic acclimation in tissues and structural adaptations in roots [19,20].

Subtropical grain legumes are classified as moderately tolerant to salinity [21,22], but some species are sensitive [23,24,25]. The specific ion effects of salinity have been associated with Na^+^ and/or Cl^−^ accumulation in soybean [26,27], mungbean [28,29], cowpea [30,31], and common bean [32], whereas in some other crops, such as rice and chickpea, the accumulation of Na^+^ in leaves has been highlighted as the major ion determining the adverse effects of salinity [33,34].

For soybean seedlings exposed to iso-osmotic solutions of 150 mM Na^+^ salts, Cl^−^ salts, and NaCl, the Cl^−^ salt treatment caused more leaf damage to *G. max* (cvs. Nannong 1138–2 and Zhongzihuangdou–yi) than Na^+^ salts, while the reverse was true for *Glycine soja* (cvs. BB52 and N23232) [26], although the treatment time was only short. That high Cl^−^ might damage soybean was also evident in a field trial on 15 varieties with KCl (122 kg ha^−1^ of K and 110 kg^−1^ ha^−1^ Cl) incorporated into the soil, which revealed positive relationships between leaf scorch and Cl^−^ concentrations in leaves and seeds, as well as negative relationships between leaf scorch, seed weight, and yield [27]. In the same study, the average Cl^−^ levels in soybean leaves increased from 34 µmol g^−1^ dry mass (0.12% *w*/*w*) in control plants to 264 µmol g^−1^ dry mass (0.94% *w*/*w*) in plants receiving KCl; the five susceptible genotypes had double the average leaf and seed Cl^−^ levels (470 µmol g^−1^ dry mass or 1.67% *w*/*w*) than tolerant genotypes (225 µmol g^−1^ dry mass or 0.8% *w*/*w*); hence, it was concluded that high tissue Cl^−^ levels can limit yield in soybean [27]. In another study, the apparent Cl^−^ toxicity level in soybean leaves after 17 days of salinity treatment (solution culture) ranged from 0.53 to 8.63% *w*/*w* (about 150–2400 µmol g^−1^ dry mass) [35].

Knowledge of specific ion responses of other (sub)tropical grain legumes is even less than that summarised above for soybean. For common bean in a light calcareous sandy loam with 25% of exchangeable Na^+^, 79% soluble Na^+^, and conductivity of 3.0 mmhos cm^−1^, leaf Na^+^ accumulation ranged from 0.1 to 1% *w*/*w* or 40 to 400 µmol g^−1^ dry mass, and growth was reduced [15]. Sodium concentrations in the injured leaves reached 1.0% w/w or 400 µmol g^−1^ dry mass, higher than the level considered toxic in bean (0.7% *w*/*w*) [36]. However, tissue Cl^−^ concentration was not measured in this study [15], so any possible effect of Cl^−^ was unknown. For cowpea exposed to 32 days of salinity treatments with electrical conductivities (saturated paste extract, EC_e_) of 1.3 to 13.8 dS m^−1^ in a sandy loam soil, shoot dry mass declined by 50% with shoot Na^+^ of 1.4% *w*/*w* (560 µmol g^−1^ dry mass) [8]. In contrast, shoot dry mass of soybean decreased by 50% with shoot Na^+^ of 0.22% *w*/*w* (80 µmol g^−1^ dry mass), indicating a greater sensitivity of soybean to tissue Na^+^ than for cowpea. In addition, the relative shoot yield declined by 50% in cowpea at 3.4% (*w*/*w* or 960 µmol g^−1^ dry mass) shoot Cl^−^ concentration, and in soybean at about 1.9% (*w*/*w* or 530 µmol g^−1^ dry mass), suggesting that higher Cl^−^ can be tolerated in shoot tissues than Na^+^ [8]. However, the study was conducted during the vegetative stage; differences in Na^+^ and Cl^−^ may occur in reproductive organs, and reproductive processes are sensitive to these ions, so further evaluations of the effects of these ions are needed for both the vegetative and reproductive stages of (sub)tropical grain legumes.

Physiological mechanisms for salt tolerance in grain legumes need to be evaluated further, as there are conflicting reports on tolerance mechanisms to salinity in some grain legumes [37]. The disagreement is whether Na^+^, Cl^−^, or both ions induce ion toxicity effects in plants. Specific ion effects of salinity stress on plant metabolism have been associated with Na^+^ and/or Cl^−^ accumulation to apparently toxic levels [38]. When the effects of Na^+^ and Cl^−^ were evaluated separately, Na^+^ had greater toxic effects on some plants than Cl^−^, with no relationship found between shoot dry mass and shoot Cl^−^ concentration in chickpea [34], cereals [39,40], or *G*. *soja* [26]. In contrast, Cl^−^ had greater toxic effects on *G. max* than Na^+^ in terms of leaf damage and growth [26]. Other studies that used NaCl have reported negative relationships between shoot Na^+^ and Cl^−^ concentrations and plant dry mass, and concluded that both Na^+^ and Cl^−^ could contribute to “ion toxicity” in, for example, faba bean [41], and barley [42].

Most studies on plant salt tolerance mechanisms have applied Na^+^ and Cl^−^ together, as NaCl is the major salt in the environment and is, therefore, of interest; however, it is not possible to identify each ion’s effects. Even when using alternative salts that have Na^+^ without Cl^−^ or Cl^−^ without Na^+^, it is difficult to separate the adverse effects of Na^+^ and Cl^−^ on plants because these salts introduce high levels of other counter-ions, such as K^+^, Mg^2+^, Ca^2+^, or SO_4_^2−^ [34,43]. Some experiments on grain legumes have applied Na^+^ and Cl^−^ separately, but only during the vegetative stage, and only as a single-species study, preventing direct species comparisons for understanding physiological mechanisms [12,22]. Whether Cl^−^ or Na^+^ is the main cause of ion toxicity in (sub)tropical grain legumes needs to be resolved [8,26,44]. Therefore, this study aimed to identify whether Na^+^, Cl^−^, or both ions cause ion toxicity effects in four subtropical/tropical grain legumes grown in aerated nutrient solution culture. This study evaluated growth, photosynthesis, and tissue ion concentrations in soybean (*G. max*), cowpea (*Vigna unguiculata*), mungbean (*V. radiata*), and common bean (*Phaseolus vulgaris*) exposed to NaCl, Na^+^ (without Cl^−^), and Cl^−^ (without Na^+^).

## 2. Results

### 2.1. Treatment Effects on Shoot and Root Dry Mass at Different Growth Stages

**Figure 1:**
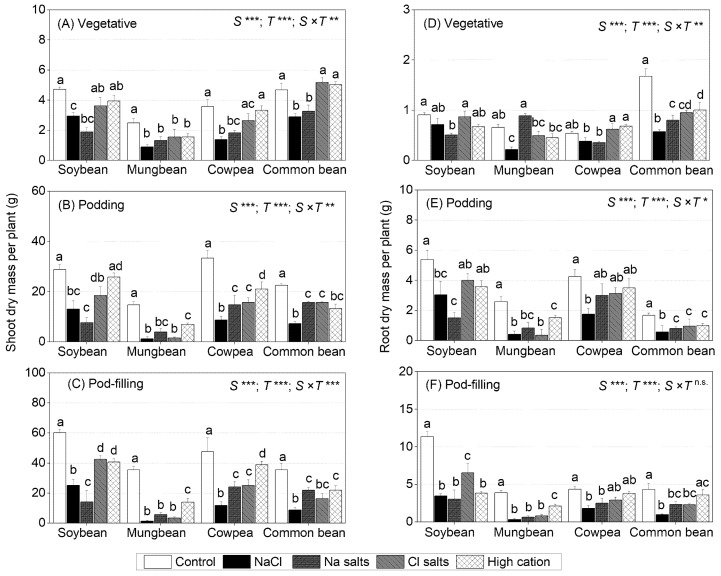
Shoot dry mass (lamina, petioles, and stems) and root dry mass per plant of soybean, mungbean, cowpea, and common bean grown in control (non-saline), 100 mM NaCl, 100 mM Na^+^ (without Cl^−^), 100 mM Cl^−^ (without Na^+^), and high-cation negative control (K^+^, Mg^2+^, and Ca^2+^ equivalent to those in the 100 mM Cl^−^ treatment) treatments. The salts used in the various treatments are given in Table 1. Treatments were imposed on 13 day-old plants and sampled after (**A**,**D**) 15 (vegetative stage), (**B**,**E**) 36 (podding stage), and (**C**,**F**) 57 (pod-filling stage) days of treatment. Values are means ± SE (*n* = 4). Significant differences for treatment means within each species are indicated by different letters (a–d) (*p* = 0.05). The probability levels for two-way analysis of variance (ANOVA) were used to compare species (S), treatment (T), and species × treatment interaction (S × T) effects (* *p* < 0.05, ** *p* < 0.01, *** *p* < 0.001, and n.s. = not significant). Note: Different scales among graphs. Dry mass of lamina, petioles and stems is presented in Supplementary Materials Figure S1; days to first leaf damage and days to first flower are presented in Table S1.

Shoot dry mass differed significantly among treatments, species, and the species × treatment interaction at the three growth stages (sampling times) (Figure 1A–C). Similar significant differences were found for root dry mass at the vegetative and podding stages, but there was no species × treatment interaction at the pod-filling stage (Figure 1D–F).

For soybean, at the vegetative stage, the NaCl and Na^+^ salts (without Cl^−^) treatments reduced shoot dry mass (68% and 39% of control, respectively), but the Cl^−^ salts (without Na^+^) and the high-cation negative control treatments did not impair shoot dry mass as compared to the non-saline control (Figure 1A). At the podding and pod-filling stages, the shoot dry mass of soybean continued to be adversely impacted by the NaCl (45% and 41%, respectively) and Na^+^ salts (without Cl^−^) (26% and 23% of control, respectively) treatments (Figure 1B,C); this also occurred in the Cl^−^ salt (without Na^+^), but to a much lesser extent than that caused by Na^+^ salts (without Cl^−^) (64% of control for the podding stage and 70% for the pod-filling stage). The high-cation negative control had no effect on the shoot dry mass of soybean at the podding stage, but some reduction occurred at the pod-filling stage, which was similar to that of Cl^−^ salts (without Na^+^), relative to the non-saline control (Figure 1B,C). For root dry mass, NaCl, Cl^−^ salts (without Na^+^), and high-cation negative control had no significant effects at the vegetative stage, but Na^+^ salts (without Cl^−^) reduced root dry mass to 55% of the control (Figure 1D). At the podding stage, the root dry mass in soybean was decreased with Na^+^ salts (without Cl^−^) (26%) and NaCl (45%), but not with Cl^−^ salts (without Na^+^) or the high-cation negative control, relative to the non-saline control (Figure 1E). Similarly, at the pod-filling stage, Na^+^ salts (without Cl^−^) and NaCl decreased the soybean root dry mass (~30% of control) more than Cl^−^ salts (without Na^+^) (57% of control). Interestingly, by the pod-filling stage in the high-cation negative control, the root dry mass of soybean had also decreased (33% of control) (Figure 1F). Overall, Na^+^ had a greater adverse effect on the soybean shoot and root growth than Cl^−^, which was evident during vegetative and reproductive growth.

For mungbean, at the vegetative stage, NaCl, Na^+^ salts (without Cl^−^), Cl^−^ salts (without Na^+^), and the high-cation negative control reduced the shoot dry mass to about 50% of the control (Figure 1A). At the podding and pod-filling stages, NaCl, Cl^−^ salts (without Na^+^), and Na^+^ salts (without Cl^−^) reduced the shoot dry mass to 20% (at podding stage) and 10% (at pod-filling stage) of the control, while the high-cation negative control had less impact on shoot dry mass (47% and 39% of the control at the podding and pod-filling stages, respectively) (Figure 1B). The root dry mass at the vegetative stage in mungbean significantly decreased with NaCl (33% of control), Cl^−^ salts (without Na^+^) (74% of control), and high-cation negative control (68% of control), but not with Na^+^ salts (without Cl^−^) (Figure 1D). At the podding and pod-filling stages, all treatments significantly reduced the root dry mass in mungbean, more so with NaCl, Na^+^, and Cl^−^ salts than with the high-cation negative control (Figure 1E,F). Overall, mungbean shoot growth was sensitive to all treatments during vegetative and reproductive growth, particularly NaCl.

For cowpea, at the vegetative stage, the shoot dry mass significantly declined (on average, 44% of control) with NaCl and Na^+^ salts (without Cl^−^), but not with Cl^−^ salts (without Na^+^) or the high-cation negative control (Figure 1A). In cowpea, at the podding and pod-filling stages, NaCl decreased shoot dry mass the most (25% of the control at the podding and pod-filling stages), followed by Na^+^ salts (without Cl^−^) and Cl^−^ salts (without Na^+^) (45% of the control at the podding stage and 50% at the pod-filling stage) and the high-cation negative control (63% of the control at the podding stage and 81% at the pod-filling stage) (Figure 1B,C). The root dry mass significantly declined at all three growth stages with NaCl (70%, 40%, and 19% of the control, respectively), but only at some stages with Na^+^ salts (without Cl^−^), Cl^−^ salts (without Na^+^), and the high-cation negative control (Figure 1D–F). Overall, NaCl and Na^+^ (without Cl^−^) reduced the shoot and root growth in cowpea during vegetative and reproductive growth, and Cl^−^ salts (without Na^+^) had an adverse effect with time.

For common bean, at the vegetative stage, NaCl and Na^+^ salts (without Cl^−^) significantly reduced the shoot dry mass (on average, 40% of the control), while Cl^−^ salts (without Na^+^) and the high-cation negative control had less of an effect (60% of control) (Figure 1A). At the podding and pod-filling stages, NaCl reduced shoot dry mass the most (32% of the control at the podding stage and 24% at the pod-filling stage), followed by Na^+^ salts (without Cl^−^), Cl^−^ salts (without Na^+^), and the high-cation negative control (on average, 40% of the control at the podding stage and 56% at the pod-filling stage) (Figure 1B,C). The root dry mass at the vegetative stage decreased the most with NaCl and Na^+^ salts (without Cl^−^) (on average, 66% of the control), while Cl^−^ salts (without Na^+^) and the high-cation negative control had no effect on root growth (Figure 1D). At the podding stage, all treatments had a significant adverse impact on root growth in common bean, declining to 31% of the control with NaCl and 66% with Na^+^ salts (without Cl^−^), Cl^−^ salts (without Na^+^), and the high-cation negative control (Figure 1E). At the pod-filling stage, NaCl remained the greatest inhibitor to root growth in common bean (22% of the control), followed by Na^+^ salts (without Cl^−^) and Cl^−^ salts (without Na^+^) (on average, 50% of the control), and no significant effect was found in the high-cation negative control relative to the non-saline control (Figure 1F). Overall, NaCl had the greatest adverse effect on shoot and root growth in common bean, which was evident during vegetative and reproductive growth.

### 2.2. Treatment Effects on Plant Reproductive Attributes

There were significant differences among treatments, species, and species × treatment interaction for total mature pod dry mass (pod walls and seeds) per plant, total mature seed dry mass per plant, and the number of mature seeds per plant (Table 2).

For soybean, Na^+^ salts (without Cl^−^) caused the greatest reduction in pod dry mass (25% of control), followed by NaCl, Cl^−^ salts (without Na^+^), and the high-cation negative control (on average, 66% of the control) (Table 2). NaCl, Na^+^ salts (without Cl^−^), and Cl^−^ salts (without Na^+^) had similar adverse effects on total seed dry mass per plant (on average, 51% of the control), while the high-cation negative control did not significantly affect seed dry mass relative to the non-saline control (Table 2).

For mungbean, NaCl, Na^+^ salts (without Cl^−^), and Cl^−^ salts (without Na^+^) each resulted in substantially less pod and seed dry mass (on average, 7% of the control) and seed number per plant (on average, 5% of the control). The high-cation negative control also reduced pod and seed dry mass, but to a lesser extent than the other treatments (Table 2).

For cowpea, NaCl reduced pod and seed dry mass and seed number per plant (~20% of control), followed by Na^+^ salts (without Cl^−^) and Cl^−^ salts (without Na^+^), and to a lesser extent, the high-cation negative control (Table 2).

For common bean, NaCl had the greatest effects on pod and seed dry mass (8% and 4% of the control, respectively), whereas Na^+^ salts (without Cl^−^), Cl^−^ salts (without Na^+^), and the high-cation negative control each also had an adverse effect on pod and seed dry mass per plant (on average, 40% of the control); these effects were not as marked as those of NaCl (Table 2).

### 2.3. Treatment Effects on Na^+^ Concentrations in Tissues

Shoot Na^+^ concentration differed significantly between species, treatments, and the species × treatment interaction at the vegetative (15 days of treatment) and pod-filling (57 days of treatment) stages, but not at the podding stage (Figure S2A–C). Root Na^+^ concentration differed significantly between species, treatments, and the species × treatment interaction at all three sampling times (Figure S2D–F).

**Figure 2:**
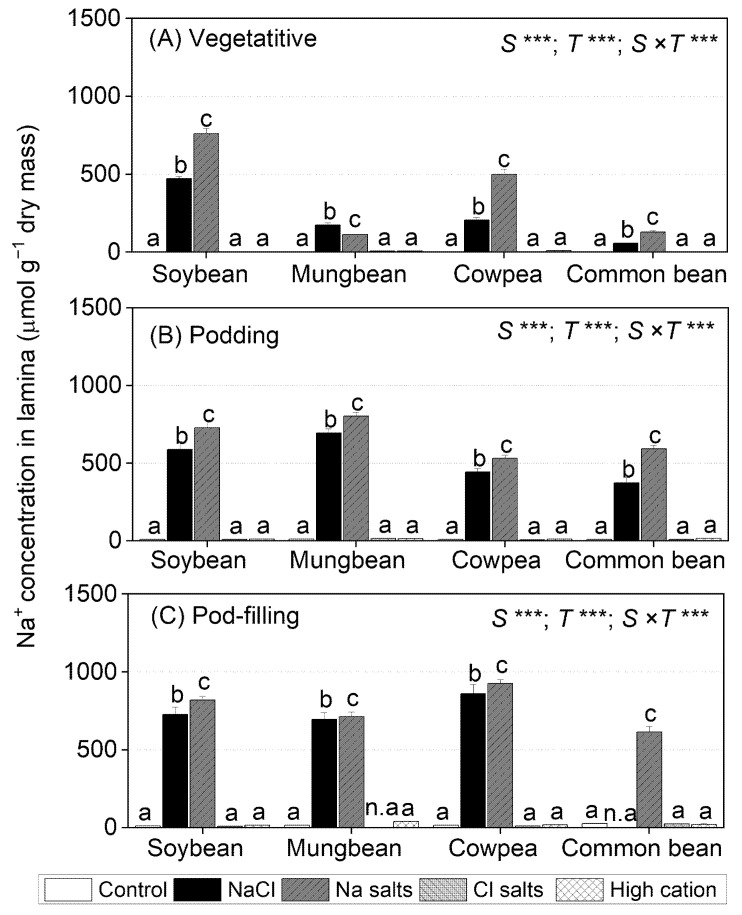
Na^+^ concentration in lamina of soybean, mungbean, cowpea, and common bean grown in control (non-saline), 100 mM NaCl, 100 mM Na^+^ (without Cl^−^), 100 mM Cl^−^ (without Na^+^), and high-cation negative control (K^+^, Mg^2+^, and Ca^2+^ equivalent to those in the 100 mM Cl^−^ treatment) treatments. The salts used in the various treatments are given in Table 1. The treatments were imposed on 13-day-old plants and sampled after (**A**) 15 (vegetative stage), (**B**) 36 (podding stage), and (C57 (pod-filling stage) days of treatment. The values are means ± SE (*n* = 4). Significant differences for treatment means within each species are indicated by different letters (a–c) (*p* = 0.05). The probability levels for two-way ANOVA were used to compare species (S), treatment (T), and species × treatment interaction (S × T) effects (*** *p* < 0.001). Note: Mungbean subjected to Cl^−^ (without Na^+^) and common bean subjected to the NaCl treatment did not have enough green leaf lamina at the pod-filling stage for ion analysis, as indicated by n.a. Na^+^ concentrations in shoots, roots, petioles, and stems are presented in Table S2, Figures S2 and S3; Na^+^ concentrations in flowers, pod walls, and seeds are presented in Figure S10.

All four species subjected to NaCl and Na^+^ salts (without Cl^−^) accumulated more root Na^+^, followed by stems, petioles, and leaf lamina, compared to the other treatments (Figure 2 and Figures S2 and S3). In contrast, all four species subjected to the non-saline control, Cl^−^ salts (without Na^+^), and high-cation negative control had low tissue Na^+^ concentrations (10 µmol g^−1^ dry mass in shoots and 30 µmol g^−1^ dry mass in roots).

In soybean, cowpea, and common bean, at the vegetative stage, Na^+^ salts (without Cl^−^) resulted in 40–60% higher leaf lamina Na^+^ concentrations than with NaCl (Figure 2A). In mungbean, by contrast, NaCl resulted in about 50% higher leaf lamina Na^+^ concentrations than with Na^+^ salts (without Cl^−^). Among the four species grown in either NaCl or Na^+^ salts (without Cl^−^), soybean had the highest leaf lamina Na^+^ concentration at the vegetative stage (759 µmol g^−1^ dry mass in Na^+^ salts), followed by cowpea (449 µmol g^−1^ dry mass in Na^+^ salts), while mungbean and common bean had the lowest (150 µmol g^−1^ dry mass in Na^+^ salts). At the podding stage, leaf lamina Na^+^ concentrations in the four species were 20–40% higher for Na^+^ salts (without Cl^−^) than for plants exposed to NaCl; mungbean had the highest Na^+^ concentration (803 µmol g^−1^ dry mass in Na^+^ salts), followed by soybean (728 µmol g^−1^ dry mass in Na^+^ salts), cowpea, and common bean (560 µmol g^−1^ dry mass in Na^+^ salts) (Figure 2B). Similarly, leaf lamina Na^+^ concentrations were significantly greater for Na^+^ salts (without Cl^−^) than NaCl at the pod-filling stage, ranging from 924 µmol g^−1^ dry mass in Na^+^ salts in cowpea to 613 µmol g^−1^ dry mass in Na^+^ salts in common bean (Figure 2C).

### 2.4. Treatment Effects on Cl^−^ Concentrations in Tissues

At the three sampling times, shoot Cl^−^ concentrations differed significantly among species, treatments, and the species × treatment interaction (Figure S4A–C). Root Cl^−^ concentrations did not differ among the four legume species at the vegetative stage, but significant differences occurred among species, treatments, and the species × treatment interaction at the podding and pod-filling stages (Figure S4D–F).

**Figure 3:**
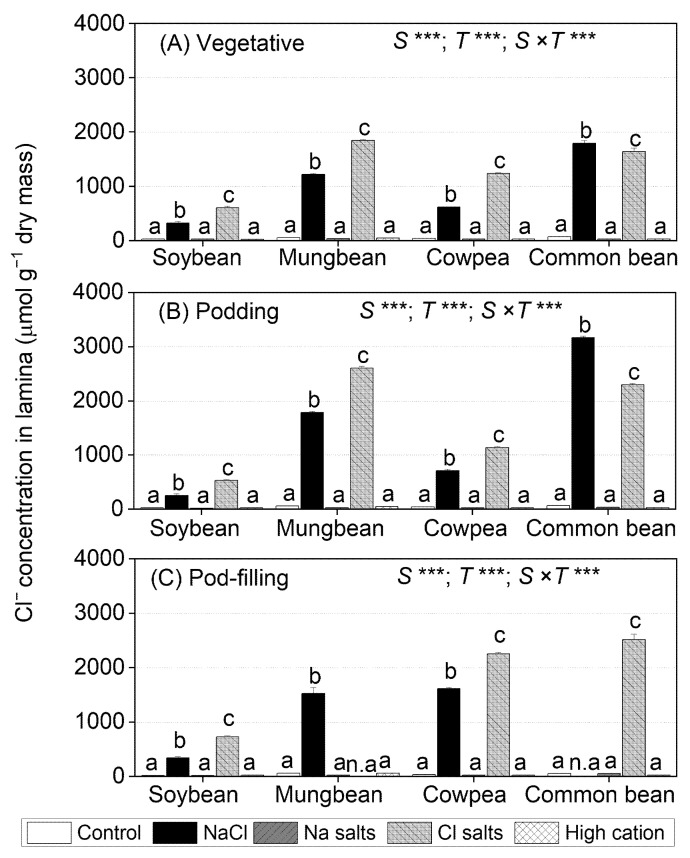
Cl^−^ concentration lamina of soybean, mungbean, cowpea, and common bean grown in control (non-saline), 100 mM NaCl, 100 mM Na^+^ (without Cl^−^), 100 mM Cl^−^ (without Na^+^), and high-cation negative control (K^+^, Mg^2+^, and Ca^2+^ equivalent to those in the 100 mM Cl^−^ treatment) treatments. The salts used in the various treatments are given in Table 1. The treatments were imposed on 13-day-old plants and sampled after (**A**) 15 (vegetative stage), (**B**) 36 (podding stage), and (**C**) 57 (pod-filling stage) days of treatment. The values are means ± SE (*n* = 4). Significant differences for treatment means within each species are indicated by different letters (a–c) (*p* = 0.05). The probability levels for two–way ANOVA were used to compare species (S), treatment (T), and species × treatment interaction (S × T) effects (*** *p* < 0.001). Note: Mungbean subjected to Cl^−^ (without Na^+^) and common bean subjected to the NaCl treatment did not have enough green leaf lamina at the pod-filling stage for ion analysis, as indicated by n.a. Cl^−^ concentrations in shoots, roots, petioles, and stems are presented in Table S2, Figures S4 and S5; Cl^−^ concentrations in flowers, pod walls, and seeds are presented in Figure S10.

The levels of Cl^−^ in different plant tissues varied among species. Soybean subjected to NaCl and Cl^−^ salts (without Na^+^) had the highest Cl^−^ concentration in roots, followed by petioles, stems, and leaf lamina (Figure 3 and Figures S4 and S5). Mungbean had the highest Cl^−^ concentrations in petioles, while cowpea and common bean had the highest Cl^−^ concentrations in leaf lamina, followed by petioles, roots, and stems (Figure 3 and Figures S4 and S5). The non-saline control, Na^+^ salts (without Cl^−^), and high-cation negative control had low tissue Cl^−^ concentrations, ranging from 20 µmol g^−1^ dry mass in soybean to 50 µmol g^−1^ dry mass in mungbean and common bean.

In the leaf lamina of soybean, mungbean, and cowpea, Cl^−^ salts (without Na^+^) produced significantly higher Cl^−^ concentrations (30–60%) than NaCl at the three sampling times (Figure 3). In contrast, NaCl produced significantly higher leaf lamina Cl^−^ concentrations in common bean than Cl^−^ salts (without Na^+^). In the NaCl treatment, leaf lamina Cl^−^ concentrations at the vegetative and podding stages were highest in common bean (1793 µmol g^−1^ dry mass at the vegetative stage and 3166 µmol g^−1^ dry mass at the podding stage), followed by mungbean (1219 µmol g^−1^ dry mass at the vegetative stage and 1783 µmol g^−1^ dry mass at the podding stage), as well as cowpea and soybean (470 µmol g^−1^ dry mass at the vegetative and podding stages) (Figure 3A,B). At the pod-filling stage, cowpea and mungbean had the highest leaf lamina Cl^−^ concentrations, and soybean had the lowest (common bean not assessed, as the plants had died) (Figure 3C). For Cl^−^ salts (without Na^+^), leaf lamina Cl^−^ concentrations at the vegetative stage ranged from 600 µmol g^−1^ dry mass in soybean to 1862 µmol g^−1^ dry mass in mungbean (Figure 3A). At the podding stage, leaf lamina Cl^−^ concentrations significantly increased in mungbean (2605 µmol g^−1^ dry mass) and common bean (2296 µmol g^−1^ dry mass), but were similar in soybean (500 µmol g^−1^ dry mass) and cowpea (1142 µmol g^−1^ dry mass) relative to those at the vegetative stage (Figure 3B). At the pod-filling stage, leaf lamina Cl^−^ concentrations ranged from 726 µmol g^−1^ dry mass in soybean to 2518 µmol g^−1^ dry mass in common bean (mungbean not assessed, as the plants had died) (Figure 3C).

### 2.5. Treatment Effects on K^+^ Concentrations in Tissues

The shoot and root K^+^ concentrations differed significantly among species, treatments, and the species × treatment interaction at all three sampling times (Figure S6). In all four species, NaCl and Na^+^ salts (without Cl^−^) reduced the shoot and root K^+^ concentrations relative to non-saline controls, with the reduction increasing with time (Figure S6). In common bean subjected to NaCl and Na^+^ salts (without Cl^−^), the shoot and root K^+^ concentrations decreased significantly at all three sampling times and were about 20–40% higher than those for Cl^−^ salts (without Na^+^). The Cl^−^ salts (without Na^+^) had no effect on shoot K^+^ concentrations at the vegetative stage, but they declined at the podding and pod-filling stages to a lesser extent than NaCl and Na^+^ salts. The high-cation negative control (with elevated K^+^) increased or maintained shoot and root K^+^ concentration in all species at all sampling times (Figure S6).

**Figure 4:**
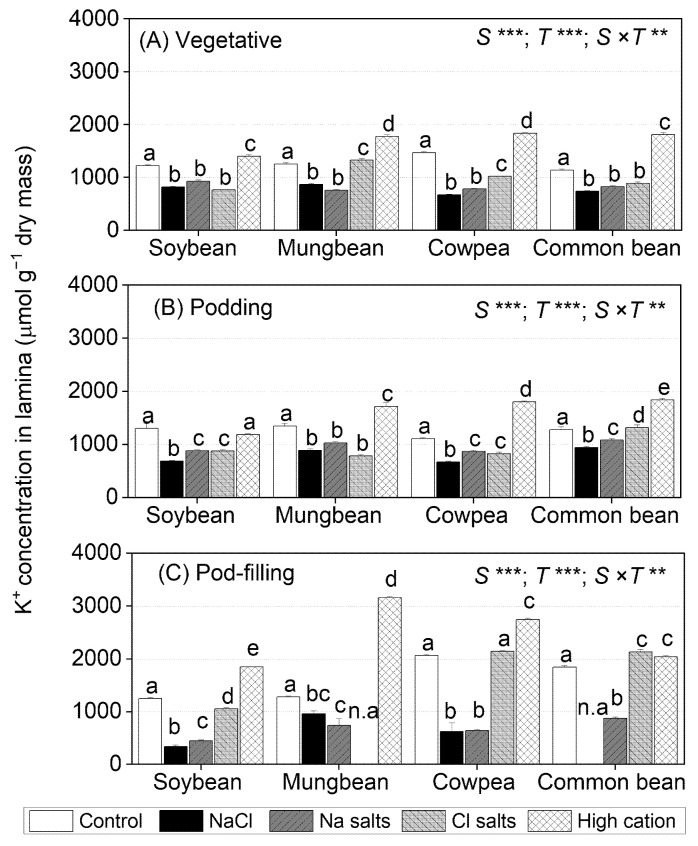
K^+^ concentrations in lamina of soybean, mungbean, cowpea, and common bean grown in control (non-saline), 100 mM NaCl, 100 mM Na^+^ (without Cl^−^), 100 mM Cl^−^ (without Na^+^), and high-cation negative control (K^+^, Mg^2+^, and Ca^2+^ equivalent to those in the 100 mM Cl^−^ treatment) treatments. The salts used in the various treatments are given in Table 1. The treatments were imposed on 13-day-old plants and sampled after (**A**) 15 (vegetative stage), (**B**) 36 (podding stage), and (**C**) 57 (pod-filling stage) days of treatment. The values are means ± SE (*n* = 4). Significant differences for treatment means within each species are indicated by different letters (a–d) (*p* = 0.05). The probability levels for two-way ANOVA were used to compare species (S), treatment (T), and species × treatment interaction (S × T) effects (** *p* < 0.01 and *** *p* < 0.001). Note: Mungbean subjected to Cl^−^ (without Na^+^) and common bean subjected to the NaCl treatment did not have enough green leaf lamina at the pod-filling stage for ion analysis, as indicated by n.a. K^+^ concentrations in shoots, roots, petioles, and stems are presented in Table S2, Figures S6 and S7; K^+^ concentrations in flowers, pod walls, and seeds are presented in Table S4, Figure S10.

At the vegetative stage, NaCl and Na^+^ salts (without Cl^−^) significantly decreased leaf lamina K^+^ concentrations in soybean and cowpea (~80% of control), while Cl^−^ salts (without Na^+^) reduced leaf lamina K^+^ concentrations in mungbean and common bean (Figure 4A). At the podding stage, NaCl, Na^+^ salts (without Cl^−^), and Cl^−^ salts (without Na^+^) reduced leaf lamina K^+^ concentrations by about 50% in soybean, mungbean, and cowpea, but only NaCl and Na^+^ salts (without Cl^−^) reduced leaf lamina K^+^ concentrations in common bean (Figure 4B). At the pod-filling stage, leaf lamina K^+^ concentrations continued to decline with NaCl and Na^+^ salts (without Cl^−^) in soybean and cowpea (30% of control), as well as mungbean and common bean (60% of control), but Cl^−^ salts (without Na^+^) maintained leaf lamina K^+^ concentrations in cowpea and common bean and slightly reduced them in soybean (80% of control) (Figure 4C). The high-cation negative control increased leaf lamina K^+^ concentrations in all species at all sampling times, except soybean at the podding stage.

### 2.6. Treatment Effects on the K^+^/Na^+^ Ratio in Tissues

The shoot and root K^+^/Na^+^ ratios differed significantly among species, treatments, and the species × treatment interaction at all three sampling times (Figure S8). For all four species, NaCl and Na^+^ salts (without Cl^−^) caused the greatest reductions in shoot and root K^+^/Na^+^ ratios relative to the non-saline control at all sampling times (Figures S8 and S9). Cl^−^ salts (without Na^+^) reduced the shoot and root K^+^/Na^+^ ratio in mungbean and common bean at the vegetative stage and in all species at the podding and pod-filling stages, but to a lesser extent than NaCl and Na^+^ salts (without Cl^−^). The high-cation negative control had an inconsistent impact on shoot and root K^+^/Na^+^ ratios in the four species (Figure S8).

**Figure 5:**
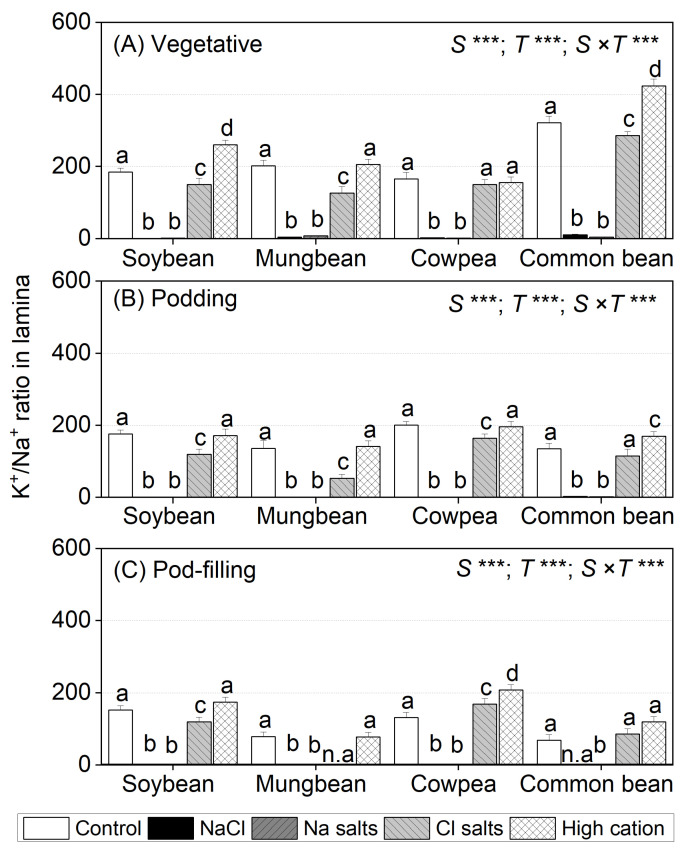
K^+^/Na^+^ ratios in lamina of soybean, mungbean, cowpea, and common bean grown in control (non-saline), 100 mM NaCl, 100 mM Na^+^ (without Cl^−^), 100 mM Cl^−^ (without Na^+^), and high-cation negative control (K^+^, Mg^2+^ and Ca^2+^ equivalent to those in the 100 mM Cl^−^ treatment) treatments. The salts used in the various treatments are given in Table 1. The treatments were imposed on 13-day-old plants and sampled after (**A**) 15 (vegetative stage), (**B**) 36 (podding stage), and (**C**) 57 (pod-filling stage) days of treatment. The values are means ± SE (*n* = 4). Significant differences for treatment means within each species are indicated by different letters (a–d) (*p* = 0.05). The probability levels for two-way ANOVA were used to compare species (S), treatment (T), and species × treatment interaction (S × T) effects (*** *p* < 0.001). Note: Mungbean subjected to Cl^−^ (without Na^+^) and common bean subjected to the NaCl treatment did not have enough green leaf lamina at the pod-filling stage for ion analysis, as indicated by n.a. K^+^/Na^+^ ratios in shoots, roots, petioles, and stems are presented in Tables S2 and S3, Figures S8 and S9; K^+^/Na^+^ ratios in flowers, pod walls, and seeds are presented in Table S4, Figure S10.

For leaf lamina, NaCl and Na^+^ salts (without Cl^−^) significantly decreased the K^+^/Na^+^ ratio in all species to about 4.0 at the vegetative stage, with further reduction at the podding (1.5) and pod-filling stages (1.0) (Figure 5A). Cl^−^ salts (without Na^+^) decreased the K^+^/Na^+^ ratio in soybean (80 and 60% of control at the vegetative and podding stages, respectively) and mungbean (40% of control at the vegetative and podding stages), but had an inconsistent effect in cowpea and common bean (Figure 5B,C). The high-cation negative control maintained the leaf lamina K^+^ concentration in all species at all sampling times relative to the non-saline control (Figure 5).

### 2.7. Treatment Effects on Leaf Gas Exchange at the Vegetative Stage

**Figure 6:**
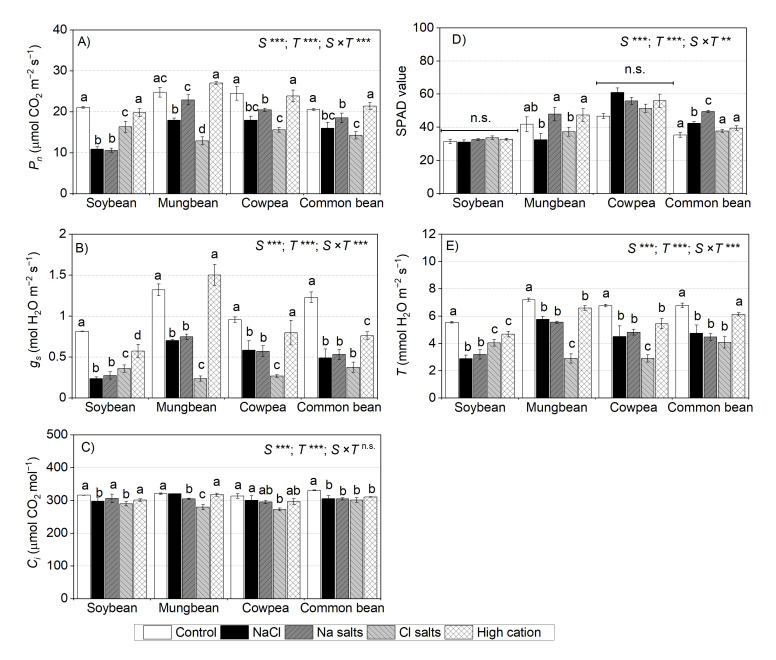
Net photosynthetic rate (**A**) (*P_n_*), (**B**) stomatal conductance (*g_s_*), (**C**) sub-stomatal CO_2_ (internal CO_2_ concentration (*C_i_*), (**D**) chlorophyll concentration (SPAD value), and (**E**) transpiration rate (*T*) of soybean, mungbean, cowpea, and common bean grown in control (non-saline), 100 mM NaCl, 100 mM Na^+^ (without Cl^−^), 100 mM Cl^−^ (without Na^+^), and high-cation negative control (K^+^, Mg^2+^ and Ca^2+^ equivalent to those in the 100 mM Cl^−^ treatment) treatments. The salts used in the various treatments are given in Table 1. The treatments were imposed on 13-day-old plants, with gas exchange measured after 13–14 days of treatment (vegetative stage) between 09:00 and 15:00 at a photosynthetically active radiation of 1500 µmol photons m^–2^ s^−1^, CO_2_ concentration of 400 µmol mol^−1^, leaf chamber temperature of 28 °C, and 60–70% relative humidity. The values are means ± SE (*n* = 4). Significant differences for treatment means within each species are indicated by different letters (a–c) (*p* = 0.05). The probability levels for two-way ANOVA were used to compare species (S), treatment (T), and species × treatment interaction (S × T) effects (** *p* < 0.01, *** *p* < 0.001, and n.s. = not significant). Note: Different scales among graphs.

For soybean, at the vegetative stage, NaCl, Na^+^ salts (without Cl^−^), and Cl^−^ salts (without Na^+^) significantly reduced the net photosynthetic rates (*P_n_*), stomatal conductance (*g_s_*), and transpiration (*T*), more so with NaCl and Na^+^ salts (without Cl^−^) (on average, 50% of the control) than Cl^−^ salts (without Na^+^) (on average, 77% of the control) (Figure 6A–C). The high-cation negative control did not affect *P_n_* in soybean (Figure 6A). The *C_i_* of soybean subjected to NaCl and Cl^−^ salts (without Na^+^) remained within 5% of the non-saline control, and in the Na^+^ salts (without Cl^−^), the *Ci* was not affected (Figure 6C). There was no significant effect of any treatments on chlorophyll concentration in leaf lamina (SPAD values) (Figure 6D).

For mungbean, at the vegetative stage, Cl^−^ salts (without Na^+^) had a significant adverse effect on *P_n_* (51% of the control), *g_s_* (17% of the control), and *T* (40% of the control) relative to NaCl (75%, 50%, and 79% of the control in P_n_, *G_s_*, and *T*, respectively) and Na^+^ salts (without Cl^−^) (90%, 56%, and 77% of the control in *P_n_*, *g_s_*, and *T*, respectively) (Figure 6A,B,E). There were small reductions in *C_i_* (<10%) in plants subjected to NaCl or Cl^−^ salts (without Na^+^) (Figure 6C). Mungbean subjected to Na^+^ salts (without Cl^−^) and the high-cation negative control had similar leaf SPAD values to those of the non-saline control, while those subjected to NaCl and Cl^−^ salts (without Na^+^) had 10–20% lower leaf SPAD values than those of the non-saline control (Figure 6D).

For cowpea, NaCl, Na^+^ salts (without Cl^−^), and Cl^−^ salts (without Na^+^) had a significant adverse impact on *P_n_*, *g_s_*, and *T*, while the high-cation negative control did not affect *P_n_* or *C_i_* (Figure 6A,B,E). NaCl and Cl^−^ salts (without Na^+^) had more adverse effects on *P_n_* (68% of the control) than Na^+^ salts (without Cl^−^) (83% of the control) (Figure 6A). The *g_s_* and *T* were the most affected by Cl^−^ salts (without Na^+^) (27% of the control for *g_s_* and 40% of the control for *T*), followed by NaCl and Na^+^ salts (without Cl^−^) (60% of the control for *g_s_* and 70% of the control for *T*) (Figure 6B,E). NaCl, Na^+^ salts (without Cl^−^), and the high-cation negative control did not affect *C_i_* in cowpea leaves, while Cl^−^ salts (without Na^+^) slightly reduced *C_i_* (15% of the control) (Figure 6C). There was no significant effect of any treatment on the leaf SPAD values (Figure 6D).

For common bean, *P_n_* significantly decreased when subjected to the salinity treatments (Figure 6A,B), more so with Cl^−^ salts (without Na^+^) and NaCl (73% of the control) than Na^+^ salts (without Cl^−^) (89% of the control). The high-cation negative control had no effect on *P_n_* relative to the non-saline control. NaCl, Na^+^ salts (without Cl^−^), and Cl^−^ salts (without Na^+^) reduced *g_s_* (~40% of the control) and *T* (~65% of the control). The high-cation negative control reduced *g_s_* (61% of the control), but did not impair *T* of common bean (Figure 6B,E). The *C_i_* in leaves declined by about 10% in all plants subjected to salinity treatments and the high-cation negative control (Figure 6C). Interestingly, none of the treatments affected leaf SPAD values (Figure 6D).

### 2.8. Treatment Effects on Leaf Sap Osmotic Potential (Ψπ)

Leaf sap osmotic potential (Ψπ_sap_) at each sampling time differed significantly among species, treatments, and the species × treatment interaction (Table 3).

For soybean, leaf sap Ψπ was significantly more negative than that of the external solution bathing the roots at all sampling times (Table 3 and Table S6). Interestingly, the change in leaf sap Ψπ was about double that of the change in the external medium in the NaCl treatment at the vegetative stage and Na^+^ salts (without Cl^−^) at the pod-filling stage. At the vegetative stage, soybean with the NaCl treatment had the most negative leaf sap Ψπ relative to the non-saline control and other salt treatments. However, at the pod-filling stage, plants subjected to Na^+^ salts (without Cl^−^) had the most negative leaf sap Ψπ relative to the non-saline control and other salt treatments, as well as the external solution bathing the roots.

For mungbean, changes in leaf sap Ψπ with NaCl and Cl^−^ salts (without Cl^−^) were about three times that of the external solution bathing the roots at the vegetative and podding stages (Table 3 and Table S6). No data were available for leaf sap Ψπ in mungbean subjected to NaCl and Cl^−^ salts (without Na^+^) at the pod-filling stage due to the lack of green leaves. The leaf sap Ψπ for plants treated with Na^+^ salts (without Cl^−^) was significantly more negative than that for the non-saline control, but less negative than that for plants treated with NaCl, Cl^−^ salts (without Na^+^), and the high-cation negative control (except at the pod-filling stage).

For cowpea subjected to NaCl and Cl^−^ salts (without Na^+^), changes in leaf sap Ψπ were less than those in the external solution bathing the roots at the vegetative stage (Table 3 and Table S6), while cowpea subjected to Na^+^ salts (without Cl^−^) and the high-cation negative control had similar changes in leaf sap Ψπ to those in the external solution. The leaf sap Ψπ of cowpea subjected to the salinity treatments and high-cation negative control was significantly more negative than that of the non-saline control at the vegetative stage, but no differences were evident at the podding and pod-filling stages.

For common bean subjected to NaCl and Cl^−^ salts (without Cl^−^), changes in leaf sap Ψπ were double those in the external solution bathing the roots at the podding and pod-filling stages (Table 3 and Table S6). Leaf sap Ψπ was significantly more negative with NaCl, Na^+^ salts (without Cl^−^), and Cl^−^ salts (without Na^+^) relative to the non-saline control at all sampling times. The change in leaf sap Ψπ in the high-cation negative control was either higher or lower than the non-saline control.

### 2.9. Relationships of Shoot Dry Mass with Shoot Ion Concentrations

Regression analyses were undertaken to assess possible relationships of shoot dry mass with shoot ion concentrations (Na^+^, Cl^−^, K^+^, and K^+^/Na^+^) (Figures S12–S15).

Shoot dry mass and shoot Na^+^ concentration (stems, petioles, and lamina) had a strong negative relationship in all four species at the vegetative stage in soybean, in cowpea and common bean at the podding and pod-filling stages, and in mungbean at the pod-filling stage (Figure S12).

No relationship was found between shoot dry mass and shoot Cl^−^ concentration in soybean at any stage (Figure S13) or in mungbean at the vegetative stage, but a negative relationship was evident at the podding and pod-filling stages. No relationship was found between shoot dry mass and shoot Cl^−^ concentration for cowpea and common bean at the vegetative or podding stages, but a negative relationship was evident at the pod-filling stage.

Shoot dry mass and shoot K^+^ concentration had a positive relationship for soybean and cowpea at all three sampling times (Figure S14). For mungbean, no relationship was found for shoot dry mass and shoot K^+^ concentration at the vegetative or pod-filling stages, but a positive relationship was evident at the podding stage. For common bean, shoot dry mass had a positive relationship with shoot K^+^ concentration at the vegetative stage.

Shoot dry mass and shoot K^+^/Na^+^ ratio had a positive relationship for soybean, cowpea, and mungbean at all three stages, as well as for common bean at the podding stage (Figure S15).

### 2.10. Relationships of Net Photosynthetic Rate (P_n_), Stomatal Conductance (g_s_), Intercellular CO_2_ (C_i_), and Transpiration Rate (T) with Leaf Ion Concentrations

Regression analyses were undertaken between *P_n_* and *g_s_* and *P_n_* and *C_i_* at the vegetative stage (Figure S16). *P_n_* and *g_s_* had a positive relationship in all four species at the vegetative stage, as did *P_n_* and *C_i_* in mungbean, cowpea, and common bean, but not in soybean.

**Figure 7:**
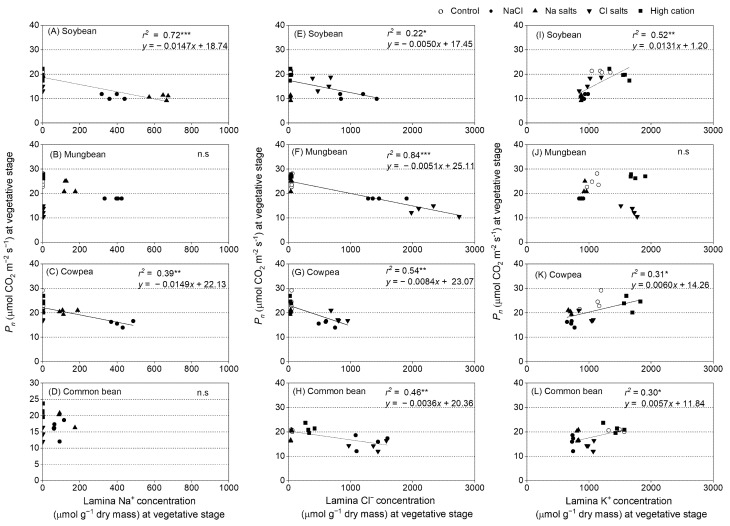
Scatter plots of net photosynthesis (*P_n_*) versus (**A**–**D**) lamina Na^+^ concentration, (**E**–**H**) lamina Cl^−^ concentration, (**I**–**L**) and lamina K^+^ concentration of soybean, mungbean, cowpea, and common bean grown in control (non-saline; open circles), 100 mM NaCl (solid circles), 100 mM Na^+^ (without Cl^−^) (solid, upward triangles), 100 mM Cl^−^ (without Na^+^) (solid, downward triangles), and high-cation negative control (solid squares) (K^+^, Mg^2+^, and Ca^2+^ equivalent to those in the 100 mM Cl^−^) treatments. The salts used in the various treatments are given in Table 1. Treatments were imposed on 13 day-old plants with gas exchange measured after 13–14 (vegetative stage) days of treatment between 09:00 and to 15:00 at photosynthetically active radiation of 1500 µmol photons m^–2^ s^−1^, CO_2_ concentration of 400 µmol mol^−1^, 28 °C leaf chamber temperature, and 60–70% relative humidity. Each value is an individual replicate, and each replicate is one plant grown in a different pot. * significant at *p* < 0.05, ** significant at *p* < 0.01, *** significant at *p* < 0.001, and n.s. = not significant.

Regression analyses were undertaken between *P_n_*, g_s,_ *T,* and ion concentrations in the lamina of the second-youngest fully expanded leaf at 13 and 14 days of treatment (Figure 7, Figures S17 and S18). *P_n_* had a negative relationship with Na^+^ and Cl^−^ concentrations in the leaf lamina in soybean (Figure 7A,E) and cowpea (Figure 7C,G), as well as with Cl^−^ concentration in the leaf lamina in mungbean and common bean at the vegetative stage (Figure 7F,H). *P_n_* had a positive relationship with K^+^ concentration in the leaf lamina in soybean, cowpea, and common bean (Figure 7I,K,L), but not in mungbean (Figure 7J). The stomatal conductance had similar results to those of *P_n_,* due to the dependence of *P_n_* on stomatal conductance (Figure S17). *T* had a negative relationship with Na^+^ and Cl^−^ concentrations in the leaf lamina in soybean (Figure S18A,E) and common bean (Figure S18D,H), as well as with Cl^−^ concentration in the leaf lamina in mungbean and cowpea at the vegetative stage (Figure S18F,G). *T* had a positive relationship between K^+^ concentration in the leaf lamina in soybean and common bean (Figure S18I,L), but not in mungbean and cowpea (Figure S18J,K).

## 3. Discussion

### 3.1. Soybean Is More Sensitive to High Na^+^ Than High Cl^−^

This study showed that Na^+^ toxicity severely inhibits soybean growth, as the shoot and root dry mass decreased to about 20% of the control when exposed to Na^+^ salts (without Cl^−^) and 40% of the control when subjected to NaCl, compared with about 70% of the control when exposed to Cl^−^ salts (without Na^+^) and the high-cation negative control (Figure 1; Table S1). Seed production per plant decreased the most with Na^+^ salts (without Cl^−^) (30% of the control), followed by NaCl (50% of the control) and Cl^−^ salts (without Na^+^) (60% of the control) (Table 2). In soybean, shoot dry mass had a negative relationship with shoot Na^+^ concentration at the three sampling times (*r*^2^ = 0.63, *r*^2^ = 0.56, and *r*^2^ = 0.65; Figure S12), but no significant relationship was found between shoot dry mass and shoot Cl^−^ concentration (Figure S13). The finding showing greater sensitivity of soybean to Na^+^ than Cl^−^ is important, since earlier studies (discussed below) had not clarified the relative roles of these ions in the response of soybean to salinity.

The lack of a clear relationship between tissue Cl^−^ concentration and soybean growth is similar to findings in a field trial with six soybean genotypes under five NaCl irrigation levels [45]. The authors found that stem and leaf Cl^−^ concentrations increased 10–15 times in intermediate- and low-tolerance genotypes relative to tolerant genotypes, but there was no relationship between stem and leaf Cl^−^ concentrations and their respective dry masses. In addition, soybean mortality occurred at stem and leaf Cl^−^ concentrations above 15,000 and 30,000 ppm, respectively, but may have been due to factors other than Cl^−^ due to the lack of a relationship between Cl^−^ concentrations and dry mass. However, the study did not include a Na^+^ analysis, so the potential impact of Na^+^ on plants was ignored [45]. Others have suggested that plants might uptake Cl^−^ for osmotic adjustment under salt stress [46]. Hence, the effect of elevated tissue Cl^−^ on plant functioning needs further investigation.

Some studies have concluded that soybean (*Glycine max*) is more sensitive to high Cl^−^ than high Na^+^ because soybean exposed to NaCl had much higher leaf Cl^−^ than Na^+^ concentrations, and a positive relationship between foliar injury and high tissue Cl^−^ concentrations was found [45,47]; however, doubts remain, as both Cl^−^ and Na^+^ were present. Another study used various salts to separate Na^+^ and Cl^−^ exposure based on a Na^+^ treatment containing concentrated macronutrient anions with 150 mM Na^+^ and a Cl^−^ treatment containing concentrated macronutrient cations with 150 mM Cl^−^ [26]. The Cl^−^ salt treatment caused more leaf damage to *G. max* than Na^+^ salts, and the authors concluded that *G. max* was more sensitive to Cl^−^ than to Na^+^_,_ while the reverse was true for *Glycine soja* [26]. In addition, *G. max* had greater relative electrolyte leakage of the first fully expanded leaf from plants with Cl^−^ salts than with Na^+^ salts and lower dry mass of *G. max* with Cl^−^ salts than Na^+^ salts, while *G. soja* had greater leaf relative electrolyte leakage and lower dry mass for plants exposed to Na^+^ salts compared to Cl^−^ salts and NaCl [26]. However, the treatment duration was only for six days, which might not be long enough for a clear conclusion on ion toxicity. Chickpea showed salt damage 10 days after being subjected to 30 mM NaCl (15 mM NaCl + 7.5 mM Na_2_SO_4_) [48]. In addition, polyethylene glycol (PEG–6000) was added to the Na^+^ and Cl^−^ treatments so that the osmotic potential was equal to that in 150 mM NaCl (–0.68 MPa) [48], but PEG–6000 could also affect plant growth [49]. In the present study, the treatments were imposed for 57 days with three sampling times; NaCl and Na^+^ salts (without Cl^−^) had greater adverse effects on *G. max* cv. Bunya than Cl^−^ salts (without Na^+^).

Salt-tolerant genotypes of *G. max* can be better able to control Na^+^ entering leaves by transporting less Na^+^ from roots to the shoot compared to susceptible genotypes [50,51]. Hence, most Na^+^ was held in the roots, with less in the stems and much less in the leaves of NaCl-treated salt-tolerant plants [50,51]. In contrast, most Cl^−^ accumulated in leaves, less in stems, and much less in roots [50,52,53]. In the present experiment, Na^+^ and Cl^−^ concentrations increased the most in roots and remained low in leaves (Figure 2 and Figure 3; Figures S2 and S4). Thus, the salt tolerance of *G. max* cv. Bunya appears to be related to its successful withholding of Na^+^ and/or Cl^−^ in roots and stems to reduce their concentrations in leaves.

Na^+^ concentrations in the second-youngest fully expanded leaves of soybean exposed to NaCl and Na^+^ salts (without Cl^−^) were assessed as exceeding the level regarded as potentially toxic (>0.5% or 217 µmol g^−1^ dry mass) [54] (Figure 2). In contrast, Cl^−^ concentrations in the second-youngest fully expanded leaves of plants exposed to NaCl and Cl^−^ salts (without Na^+^) did not reach the threshold level for Cl^−^ toxicity (<2.6–5.0% or 713–1407 µmol g^−1^ dry mass) [54] (Figure 3). Other research on soybean grown in solution culture reported that Cl^−^ concentrations greater than 2.84% (800 µmol g^−1^ dry mass) in the second-youngest fully expanded leaf caused soybean death after 17 days of NaCl treatment, and a wide range of leaf Cl^−^ concentrations caused toxicity (0.53–8.63% or 149–2428 µmol g^−1^ dry mass) (Table S7) [38]. In vitro studies showed that Na^+^ starts to inhibit most enzymes at concentrations approaching 100 mM [55]. In the present study, leaf Na^+^ concentrations in soybean subjected to NaCl and Na^+^ salts (without Cl^−^) were about 100 and 150 mM, respectively, on a tissue water basis, and leaf Cl^−^ concentrations in soybean subjected to NaCl and Cl^−^ salts (without Na^+^) were 70 and 140 mM, respectively (calculated from Figure 2 and Figure 3). The shoot K^+^/Na^+^ ratios of the Cl^−^ salts (without Na^+^) and high-cation negative control were about 100, while those in NaCl and Na^+^ salts (without Cl^−^) were 0.4 (Figure 5, Table S3), which could be detrimental for metabolic activities [43,56].

In addition to the large reductions in growth, *P_n_* in soybean declined in plants subjected to NaCl and Na^+^ salts (without Cl^−^) (Figure 6) and was negatively correlated with leaf Na^+^ and Cl^−^ concentrations (Figure 7A,E). However, Cl^−^ salts (without Na^+^) had a relatively marginal effect on *P_n_* (Figure 6). There was a positive relationship between *P_n_* and *g_s_* in soybean (*r*^2^ = 0.76), but no relationship between *P_n_* and *C_i_* at the vegetative stage (Figure S16A,E). Hence, *C_i_* (based on *g_s_*) was unlikely to be the cause of the reduction in *P_n_* in soybean. A salt-induced stomatal limitation of *P_n_* has been reported in plants such as soybean [57] and sorghum [58], and non-stomatal limitation of *P_n_* has been reported in chickpea [27,59]. Other studies conducted in greenhouses or growth chambers [60,61,62] and in the field reported that salt stress significantly decreases *P_n_*, *g_s_*, and Chl fluorescence parameters in soybean, and concluded that *g_s_* is a primary limiting factor for the reduction of *P_n_* under salt stress [63]. However, these experiments did not measure tissue ion concentrations. Na^+^ in leaves could have a negative impact on photosystems and/or photosynthetic metabolism in soybean, as suggested by the negative correlation of leaf Na^+^ and *P_n_* in the present study.

### 3.2. Mungbean Is More Sensitive to High Cl^−^ Than High Na^+^

Some studies have suggested that Cl^−^ toxicity might reduce growth in mungbean under saline conditions [28,64]. The present study showed that NaCl, Na^+^ salts (without Cl^−^), Cl^−^ salts (without Na^+^), and the high-cation negative control all substantially reduced shoot dry mass and seed dry mass, although more so with NaCl and Cl^−^ salts (without Na^+^) (Figure 1; Table S1; Table 2). Negative relationships occurred between mungbean shoot dry mass and shoot Na^+^ at the vegetative and pod-filling stages (*r*^2^ = 0.38 and *r*^2^ = 0.24, respectively) (Figure S12), as well as between shoot dry mass and shoot Cl^−^ at the podding and pod-filling stages (*r*^2^ = 0.37, and *r*^2^ = 0.33) (Figure S13). In addition, the highest concentrations of Na^+^ were in the roots and stems of mungbean subjected to NaCl or Na^+^ salts (without Cl^−^), with less in petioles and much less reaching the lamina; in contrast, most Cl^−^ accumulated in leaves and petioles, with less in stems, and much less in roots (Figure 2 and Figure 3) These distributions of Na^+^ and Cl^−^ are similar to those found in another study on mungbean [65]. Hence, salt-sensitive mungbean could not withhold Cl^−^ in roots to avoid leaf Cl^−^ accumulation, which could impact the photosynthetic machinery.

The *P_n_* of mungbean subjected to NaCl and Cl^−^ salts (without Na^+^) declined to about 30–50% of the control (Figure 6), and *P_n_* had a negative relationship with leaf Cl^−^ concentration (Figure 7F). In another study, salinity adversely impacted chlorophyll concentrations in mungbean (50% of control), as chlorophyll synthesis was suppressed [65]. In the present study, the SPAD values of mungbean subjected to NaCl and Cl^−^ salts (without Na^+^) were about 80% of the control (Figure 6D), and shoot Cl^−^ concentrations were about 3–4% of dry mass (845–1125 µmol g^−1^ dry mass) (calculated from Figure 2), which was much greater than the critical toxicity level (1.18% or 332 µmol g^−1^ dry mass) (Table S7) [38]. No information is available on leaf or shoot Na^+^ toxicity levels in mungbean, so there is no benchmark for comparison with the present study.

Mungbean subjected to the high-cation negative control suffered reduced shoot dry mass (60%, 50%, and 40% of control at the vegetative, podding, and pod-filling stages, respectively), but these plants still had more dry mass than the plants subjected to Na^+^ salts (without Cl^−^), Cl^−^ salts (without Na^+^), or NaCl (average of 50%, 15%, and 10% of control at vegetative, podding, and pod-filling stages, respectively). The reduction in mungbean growth in the high-cation negative control could be due to the osmotic effect of high ion concentrations or a specific ion effect from one or more of the ions used (cf. chickpea in [34,59]).

### 3.3. Cowpea Is Sensitive to Both High Na^+^ and High Cl^−^

Na^+^ salts (without Cl^−^) and Cl^−^ salts (without Na^+^) had the same adverse effects on cowpea shoot and root dry mass, but the combined NaCl treatment had the greatest reductions in growth, so there appeared to be an additive effect of these two ions (Figure 1; Table S1). NaCl, Na^+^ salts (without Cl^−^), and Cl^−^ salts (without Na^+^) reduced seed dry mass in cowpea by about 70%, 50%, and 50%, respectively (Table 2). A similar finding was reported for fava bean and was considered the result of additive effects of Na^+^ and Cl^−^ [41]. Another study on fava bean subjected to 100 mM NaCl suggested that Na^+^ inhibits K^+^ uptake and stomatal regulation, while Cl^−^ could cause chlorophyll degradation [57]. In the present study, shoot dry mass had a negative relationship with shoot Na^+^ concentration at the three sampling times (*r*^2^ = 0.66, *r*^2^ = 0.31, and *r*^2^ = 0.36 at the vegetative, podding, and pod-filling stages, respectively) (Figure S12C,G,K) and shoot Cl^−^ concentration at the pod-filling stage (*r*^2^ = 0.27) (Figure S13C,G,K). In addition, cowpea appeared to withhold Na^+^ in the roots, with less transferred to stems and petioles, and much less to leaves; in contrast, leaf Cl^−^ concentration increased with time and was much greater than that in the roots at the pod-filling stage (Figure 2 and Figure 3). Another study of two cowpea genotypes found that biomass reduction was only weakly associated with ability to restrict Na^+^ and Cl^−^ accumulation in shoots (*r*^2^ = 0.10 and r^2^ = –0.11, *n* = 150) after 24 days of being subjected to 75 mM NaCl [30]. There is no information on the toxicity levels for Na^+^ and/or Cl^−^ concentrations in cowpea, but the levels regarded as “adequate” (i.e., non-toxic) are 0.01–0.03% *w*/*w* of Na^+^ (4–13 µmol g^−1^ dry mass) and 0.7–1.6% *w*/*w* of Cl^−^ (197–450 µmol g^−1^ dry mass) in the youngest mature leaves during the vegetative stage (Table S7) [54]. In the present study, Na^+^ concentrations in the second-youngest fully expanded leaf were 0.2% for Na^+^ salts (without Cl^−^) and 1.0% for NaCl, and Cl^−^ concentrations in the same leaves were 1.5% for NaCl and 1.7% for Cl^−^ salts (without Na^+^). These values are much higher than those reported as non-toxic in cowpea [54]. In the present study, shoot Na^+^ concentrations were greater than the shoot Cl^−^ concentrations, but the reduction in pod dry mass was similar and to a lesser extent than that of NaCl; thus, both Na^+^ and Cl^−^ impaired cowpea growth, and there might be an adverse additive effect of these two ions on cowpea growth.

Cowpea subjected to NaCl, Na^+^ salts (without Cl^−^), and Cl^−^ salts (without Na^+^) had greater reductions in shoot dry mass than the high-cation negative control, so the reduction was likely due to Na^+^ and Cl^−^ (Figure 2). In addition, cowpea subjected to NaCl, Na^+^ salts (without Cl^−^), and Cl^−^ salts (without Na^+^) had substantial reductions in *P_n_* (Figure 6). Scatter plots of leaf ion concentrations and *P_n_* showed negative relationships between *P_n_* and both Na^+^ and Cl^−^ (*r*^2^ = 0.39 and *r*^2^ = 0.54, respectively) (Figures S12 and S13). These results are similar to those of a previous study that reported significant declines in leaf *g_s_*, *P_n_,* and transpiration in cowpea subjected to NaCl, as well as a negative linear relationship between leaf Na^+^ concentration and *P_n_* after 10 days of treatment [66]. Therefore, the greater reductions in growth under NaCl than Na^+^ or Cl^−^ suggests that high concentrations of Na^+^ and Cl^−^ both limit cowpea growth.

### 3.4. Common Bean Is Sensitive to Both High Cl^−^ and to High Na^+^

Shoot and root dry mass and seed dry mass in common bean declined significantly when subjected to NaCl, Na^+^ salts (without Cl^−^), Cl^−^ salts (without Na^+^), and the high-cation negative control relative to the non-saline control, more so in the NaCl treatment (Figure 1; Table S1; Table 2). This result was similar to that for cowpea in the present study and fava bean, where high Na^+^ and Cl^−^ concentrations inhibited plant growth more than Na^+^ or Cl^−^ alone [42]. In the present study, the highest concentration of Na^+^ in common bean was in the roots, with less in the leaves, despite the leaves having about a 10% greater Cl^−^ tissue concentration than the roots (Figure 2 and Figure 3). Similarly, salt-sensitive common bean had higher leaf Na^+^ and Cl^−^ concentrations than salt-tolerant *Sesbania aculeata* [35]. Another study reported a negative relationship between growth and shoot Na^+^ concentration in common bean subjected to NaCl, but this study did not measure Cl^−^ [15]. In the present study, shoot dry mass was negatively correlated with shoot Na^+^ concentration at the three sampling times (*r*^2^ = 0.36, *r*^2^ = 0.19, and *r*^2^ = 0.26, at the vegetative, podding, and pod-filling stages, respectively), as well as shoot Cl^−^ concentration at the pod-filling stage (*r*^2^ = 0.49) (Figures S12 and S13). Hence, both Na^+^ and Cl^−^ impaired common bean growth.

NaCl, Na^+^ salts (without Cl^−^), and Cl^−^ salts (without Na^+^) also impaired *P_n_* in common bean (Figure 6), with a negative relationship between *P_n_* and leaf Cl^−^ concentration (*r*^2^ = 0.46) (Figure 7H), but interestingly no significant relationship with leaf Na^+^ concentration was found (Figure 7D). A salt-induced decrease in *P_n_* can be due to stomatal limitations; this condition occurs when *g_s_* decreases and reduces *C_i_,* which, in turn, restricts the rate of *P_n_* [42]. In the present study, the *C_i_* of common bean subjected to NaCl, Na^+^ salts (without Cl^−^), Cl^−^ salts (without Na^+^), and the high-cation negative control was about 90% of the non-saline control, and there were positive relationships between *P_n_* and *g_s_* (*r*^2^ = 0.56) and *P_n_* and *C_i_* (*r*^2^ = 0.37) (Figure S16D,H). Although the reduced stomatal conductance likely contributed to the reduced *P_n_*, high concentrations of ions in the leaves with possible adverse effects on photosystems and photosynthetic metabolism may also have contributed to the large reduction in *P_n_* in common bean (Figure 6A), since shoot Na^+^ concentration under NaCl reached a level regarded as toxic (Na^+^ 0.7% by dry mass or 305 µmol g^−1^ dry mass [38]) and shoot Cl^−^ concentration under NaCl reached critical toxic levels (>30 mmol kg^−1^ shoot water (calculated from Figures S2 and S4)) [64]. In this study, plants subjected to NaCl did not have any green leaves at the final harvest (57 days of treatment), highlighting the sensitivity of common bean to salinity.

### 3.5. Osmotic Contribution to Salinity Tolerance

To avoid tissue water deficit during salt stress, plants need to maintain their osmotic potential at levels below that of the osmotic potential of the external solution bathing the roots [67,68,69]. In this study, the leaf sap osmotic potential (Ψπ_sap_) was more negative than the external solution bathing the roots in all four legume species (Table 3 and Table S6). All four legume species showed leaf osmotic adjustment by decreasing (i.e., more negative) leaf osmotic potential and maintaining leaf water content when subjected to 100 mM NaCl at the vegetative stage. The change in leaf sap osmotic potential in all four legume species was greater than the change in the osmotic potential of the external NaCl bathing the roots (Table 3 and Table S6).

Under salt stress, plants must regulate osmotic adjustment by synthesizing organic compounds and transporting ions. These activities induce a small reduction in plant growth potential [70,71]. Therefore, when subjected to the high-cation negative control, plants had to cope with the osmotic stress, not the high Na^+^ and/or Cl^−^ stress, which might be the primary cause of the growth reduction in all four legumes (Table 1 and Table S6). For some plants exposed to salinity stress, proline and other organic compounds increase, e.g., in rice [72] and soybean [50]. In the present study, proline was not detected in the second-youngest fully expanded leaf lamina of any species, but moderate amounts of pinitol in soybean and sucrose were detected in all four legume species (Figure S11). Salt-stressed soybean leaves had about 20–40% greater molar concentration of pinitol than sucrose, which was similar to the findings of a study on drought stress, where pinitol, proline, and sugars accumulated in leaf blades of soybean plants, but pinitol had higher molar concentrations in stressed plants than proline or sugars [73]. Sucrose accumulation in leaves and roots can contribute to osmotic adjustment [74]. In the present study, no significant differences were observed in leaf sucrose concentrations for soybean and cowpea, but mungbean and common bean had significantly higher sucrose concentrations than the non-saline control (Figure S11). This finding is similar to that reported for barley and soybean, where salt stress induced sucrose accumulation in leaves and roots [43,75].

### 3.6. Soybean Is More Salinity Tolerant Than the Three Other Grain Legume Species

Yield reductions of 50% have been reported at EC_e_ of 5.0 dS m^−1^ for soybean, 4.9 dS m^−1^ for cowpea, 1.8 dS m^−1^ for mungbean, and 1.0 dS m^−1^ for common bean (Maas and Grattan 1999) based on data from several studies. While the four legume species in this study differ in salt tolerance, all four are regarded as salt sensitive when compared with, for example, barley (*Hordeum vulgare* L.) (EC_e_ of 8.0 dS m^−1^) and canola (*Brassica napus* L.) (EC_e_ of 9.0 dS m^−1^) [76]. In the present study, salinity tolerance (total mature seed dry mass in NaCl as % of non-saline control) in the four legume species, ranked from highest to lowest, was soybean = cowpea > mungbean = common bean (Table 2). Salt tolerance in soybean was evident through the higher dry mass, *P_n_,* and filled pod production compared to those of the three other species in the same salinity treatments (Figure 1 and Figure 6, Table 2). Soybean had higher or equal concentrations of Na^+^ and/or Cl^−^ in leaf tissues than the three other species (combined mean of harvests and genotypes), but maintained the greatest relative growth and pod and seed production. This study did not assess the intraspecific variations in salt tolerance of these four legume species, so the rankings could be refined with further assessment using several genotypes within each species.

The data on the ionic compositions of shoot tissues allow for speculation on the physiological basis of the differences in salt tolerance. The four (sub)tropical grain legumes had pronounced differences in the quantities of Na^+^ in lamina (Figure 2), but there was no clear explanation of the relationship between Na^+^ exclusion and salt tolerance. The (sub)tropical grain legumes differed in lamina Cl^−^ accumulation at high salt levels (Figure 3), but there was no clear relationship with salt tolerance. Highly salinity-tolerant species (halophytes) sequester Na^+^ and Cl^−^ in vacuoles and organic solutes in the cytoplasm to adjust osmotically under salt stress [9,37]. In this study, at the vegetative stage, the most tolerant species (soybean) accumulated the most Na^+^ and low Cl^−^ in lamina, while the most sensitive species (mungbean and common bean) were comparatively effective in maintaining low Na^+^ levels, but high Cl^−^, in lamina. Cowpea accumulated the highest Na^+^ and Cl^−^ in lamina (Figure 2 and Figure 3).

### 3.7. Shoots Are More Sensitive to Salinity Stress Than Roots (Three of the Four Species), and the Vegetative Stage Is Less Sensitive to Salinity Than the Reproductive Stage

In this study, salinity significantly reduced shoot dry mass in soybean, cowpea, and common bean (>50% of dry mass), with a more moderate impact on root dry mass (20% of dry mass). In mungbean, salinity reduced shoot dry mass by 70% and root dry mass by 90% (Figure 1). Other studies have reported that roots are more salinity tolerant than shoots in soybean [8,77] and common bean [78].

In addition, legume plants exposed to salinity significantly reduced shoot dry mass by 40% relative to the non-saline control at the vegetative stage, >50% at the podding stage, and about 70% at the pod-filling stage (Figure 1). A study on wheat and barley indicated greater sensitivity to salinity during the seedling stage than germination and that seedlings are more sensitive than older plants [79]. Chickpea subjected to 30 mM NaCl showed no genotypic differences at the vegetative stage, but when subjected to 60 mM NaCl, the genotypic differences were evident at the late vegetative stage [16,59]. Therefore, the present study adds to the information available on salinity tolerance regarding developmental stage, length of exposure, salinity treatments, and previously undocumented species [8,26,44].

## 4. Materials and Methods

### 4.1. Plant Materials and Growth Conditions

The experiments were conducted in a phytotron (temperature-controlled glasshouse; 28/22 ± 2 °C day/night) in summer and autumn (February to April 2017) in Perth, WA, Australia (31°57′ S, 115°47′ E). Plants received natural sunlight transmitted through polycarbonate panels, and no supplementary light was needed.

Seeds of soybean (*Glycine max* (L.) Merr.) cv. Bunya, mungbean (*Vigna radiata* (L.) R. Wilczek) cv. Jade, cowpea (*Vigna unguiculata* (L.) Walp.) cv. Red Caloona, and common bean (*Phaseolus vulgaris* L.) cv. Spearfelt were washed with 0.042% (*w*/*v*) sodium hypochlorite for 5 min, rinsed thoroughly with tap water, and placed on wet tissue paper in Al-covered 20 L buckets. Germinated seeds were placed on plastic mesh floating on 10% (*v*/*v*) strength aerated nutrient solution in the dark for two days. On day 3, the nutrient solution was changed to 25% (*v*/*v*) strength and the seedlings were exposed to natural sun light. Seven-day-old seedlings were transferred to pots (four seedlings per 4.5 L plastic pot) containing 100% (*v*/*v*) strength aerated nutrient solution (Table 1) [59]. The plastic pots were covered with aluminium foil to exclude light from the root zone. After five days, the salt treatments were imposed on 13 day-old plants.

### 4.2. Treatments

The experiment involved a non-saline control and four salt treatments (100 mM Na^+^ salts (without Cl^−^), 100 mM Cl^−^ salts (without Na^+^), 100mM NaCl, and 100 mM high-cation negative control (no Na^+^, no Cl^−^)), four legume species, and four replicates. Eighty pots (4 species × 5 treatments × 4 replications) were arranged in a completely randomized design. The pots were reorganized each week, when the solution in each pot was renewed, to minimize any possible effects of environmental variation within the phytotron.

The Na^+^ salts (without Cl^−^), Cl^−^ salts (without Na^+^), and NaCl treatments were used to elucidate the individual effects of Na^+^ and Cl^−^, as well as the possible adverse effects when combined. The high-cation treatment (no Cl^−^, no Na^+^, with the same cations as in the Cl^−^ treatment, but with SO_4_^2−^ and NO_3_^−^ as anions) was a “negative control” used to examine the effects of elevated levels of the cations accompanying Cl^−^ in the “Cl^−^ without Na^+^ treatment,” and as an osmotic treatment, albeit not as negative as that in the NaCl treatment. The composition of the full-strength nutrient solution in deionized water was (mM): 5.0 Ca^2+^, 5.0 K^+^, 0.6 NH_4_^+^, 0.4 Mg^2+^, 0.2 Na^+^, 5.4 SO_4_^2−^, 4.4 NO_3_^−^, 0.2 H_2_PO_4_^−^, 0.1 SiO_3_^2−^, 0.1 Fe-sequestrene, 0.05 Cl^−^, 0.025 BO_3_^3−^, 0.002 Mn^2+^, and 0.002 Zn^2+^. The solution was buffered with 1.0 mM MES (2-(*N*-morpholino)ethanesulfonic acid) and adjusted to pH 6.5 using KOH [60]. The ion concentrations (mM) and osmotic potential (Ψπ) of the various solutions are shown in Table 1. Treatment imposition followed Khan et al.’s [59] method, except for the higher salt concentrations. The salt treatments were imposed across four days in increments of equal size (e.g., 25 mM NaCl increments added daily to reach the 100 mM NaCl concentration). The solution in each pot was renewed weekly and topped up with deionized water as required (initially every two days for the first week and daily thereafter).

### 4.3. Plant Samplings and Measurements

One plant per pot was used for each sampling time. The first sampling occurred before treatments were imposed, with three subsequent samplings at the vegetative stage (15 days after treatment (DAT), counted from the first dose of salt addition), podding stage (36 DAT, middle of podding), and pod-filling stage (57 DAT—some pods at maturity). At each sampling, the roots and basal parts of stems were rinsed for 30 s in 5 mM CaSO_4_ (control plants) or in 5 mM CaSO_4_ + 200 mM mannitol (for salt-treated plants to avoid an osmotic shock), before being gently blotted with a paper towel to remove external water. The plants were then separated into green leaves (laminas), damaged leaves (laminas), dead leaves (excluding leaf drop), petioles, stems, and roots. Dead leaves had browned and were fully or partially desiccated, while damaged leaves were predominantly brown, but still had some green areas on leaflets. Green leaves were green and turgid, but occasionally had brown edges (less than 10% of the leaflet area). Plants were considered dead when shoots had no green leaves and stems had turned brown. All tissues were measured for fresh mass, and then for dry mass after oven-drying at 65 °C for 48–62 h. At maturity, pods were separated and counted for mature filled pods, empty pods (pods were individually opened), and seed numbers.

### 4.4. Leaf Gas Exchange and Chlorophyll Measurements

Stomatal conductance (*g_s_*), net photosynthetic rate (*P_n_*), transpiration (*T*), and internal CO_2_ concentration (*C_i_*) were measured on the second-youngest fully expanded leaf during the vegetative, podding, and pod-filling stages using an LI–6400XT open gas exchange system coupled with a 6 cm^2^ chamber head (LI–COR Biosciences Inc., Lincoln, NB, USA). The measurements were conducted at a photosynthetically active radiation of 1500 µmol photons m^−2^ s^−1^ (light-saturated) and CO_2_ concentration of 400 µmol mol^−1^ [80]. The gas exchange measurements were taken on the same day between 09:00 and 15:00 h. The leaf chamber temperature was 28 °C with 60–70% relative humidity. Chlorophyll concentrations in leaves (lamina) were measured on the same day using an SPAD meter (Minolta, Osaka, Japan) on the same leaf used for the gas exchange measurements.

### 4.5. Tissue Ion Analysis

Oven-dried samples of various tissues (green leaves, damaged leaves, dead leaves, petioles, stems, roots, flowers, seeds, and pods) of the same sampled plants were ground to a fine powder and analysed for Na^+^, K^+^, and Cl^−^ following the procedures of Munns et al. [81]. Tissues (100 mg) were extracted in 0.5 M HNO_3_ (10 mL) by shaking for 48 h in darkness at room temperature. Diluted samples of the extracts were then analysed for Na^+^ and K^+^ using a flame photometer (Flame Photometer 410, Sherwood, Cambridge, UK) and for Cl^−^ with a chloridometer (Model 50CL, SLAMED ING. GmbH, Frankfurt, Germany). A reference tissue (broccoli, ASPAC no. 85) with known ion concentrations was taken through the same analyses to confirm the reliability of the methods.

### 4.6. Organic Solutes (Sugars and Sugar Alcohols)

Tissues (lamina of the second-youngest fully expanded leaf) of the same sampled plants were frozen in liquid N_2_ and freeze-dried to preserve metabolites. Organic solutes (sugars, sugar alcohols) were extracted twice from 0.1 g of a ground leaf tissue with 3 mL of ice-cold 5% (*w*/*v*) perchloric acid, following previously described methods [82,83]. The supernatants from each extraction were combined and neutralized using K_2_CO_3_ until pH 3.0–3.5; the neutralized supernatants were collected after centrifugation at 15,000× rpm for 30 min. The neutralised extracts were analysed using high-performance liquid chromatography (HPLC), with a 600 E pump, 717 plus autoinjector, and 996 photodiode-array (PDA) detector (Waters, Milford MA, USA) with an evaporative light-scattering detector (ELSD) (Alltech, Deerfield, IL, USA). Separation was achieved with a Sugar-Pak column, as described elsewhere [82]. The Sugar-Pak column (300 × 6.5 mm i.d.) was held at 90 + 0.5 °C with separation achieved using a mobile phase of 2.5 mg L^−1^ Ca-EDTA at 0.6 mL min^−1^. Detection and quantification were undertaken with the PDA at 195 nm, with peak spectral and purity comparisons of samples made with the standards.

### 4.7. Leaf Sap Osmotic Potential and Tissue Water Content

Part of the leaf lamina of the second-youngest fully expanded leaf was sampled into a 2 mL airtight cryovial, quickly frozen in liquid N_2_, and stored at –20 °C until required. Samples were thawed in the sealed vials and then crushed in a manual press to obtain tissue sap [84]. The osmotic potential was measured using 20 µL of sap in a calibrated freezing-point depression osmometer (Fiske Associates, Model One–Ten, Haverhill, MA, USA). The readings were converted from milli osmol L^−1^ to MPa using the formula (2.447 × *X*)/1000, where *X* = is the value in milli osmol L^−1^ and 2.447 is R × T, where R is the universal gas constant (8.314472 J K^−1^ mol^−1^) and T is temperature in Kelvin (293 at 20 °C). Tissue water content was calculated using fresh and dry mass data to calculate mL g^−1^ dry mass.

### 4.8. Statistical Analyses

Data were subjected to one-way, two-way, or three-way analysis of variance (ANOVA) using Genstat Software (VSN International Ltd., Hemel Hempstead, UK) to observe differences between treatments and species and to test for any species × treatment interactions. The figure and table captions provide details on the tests used. The three-way ANOVA included a factor for the time or level of CO_2_ for some datasets. Means were compared for significant differences using least significant differences (LSDs) at the 5% probability level. Figures and scatter plots were graphed using Origin Pro 2019 (v. 9.65).

## 5. Conclusions

Four (sub)tropical grain legume species (soybean, mungbean, cowpea, and common bean) were exposed to nine weeks of salinity treatments, which were a non-saline control, 100 mM NaCl, 100 mM Na^+^ (without Cl^−^), 100 mM Cl^−^ (without Na^+^), and a high-cation negative control (K^+^, Mg^2+^, and Ca^2+^ equivalent to those in the 100 mM Cl^−^ treatment, but without Cl^−^ as the counter-ion). The results showed that salt sensitivity is determined by Na^+^ toxicity in soybean, Cl^−^ toxicity in mungbean, and Na^+^ and Cl^−^ toxicity in cowpea and common bean.

Soybean accumulated the least Na^+^ and Cl^−^ in the lamina and accumulated the most Na^+^ and Cl^−^ in the roots. Mungbean, cowpea, and common bean accumulated the least Na^+^ in the lamina, but accumulated the most Na^+^ in the roots. Mungbean and common bean accumulated the most Cl^−^ in the lamina and the least in the roots, while cowpea accumulated similar amounts of Cl^−^ in the lamina and roots.

Exclusion of Na^+^ from leaves is generally considered a feature of plants that contributes to their salinity tolerance; low leaf Na^+^ concentrations in soybean, mungbean, cowpea, and common bean relative to those of the petioles, stem, and roots suggest that the four (sub)tropical grain legume species can exclude Na^+^ from the leaves.

Na^+^ salts (without Cl^−^) affected the photosynthetic capacity of soybean and seed dry mass more than Cl^−^ salts (without Na^+^), while the reverse was true for mungbean and common bean. NaCl affected the photosynthetic capacity of cowpea and seed dry mass the most, while Na^+^ salts (without Cl^−^) and Cl^−^ salts (without Na^+^) had similar effects on cowpea photosynthesis and seed production.

Among the four (sub)tropical grain legumes, mungbean and common bean had the greatest sensitivity to salinity (100 mM NaCl), while soybean and cowpea were more tolerant. This study used single genotypes of mungbean, cowpea, and common bean; further research on more genotypes is needed to elucidate whether the findings are more generally applicable and to assess genotypic variation in various traits to further understand salt tolerance mechanisms in these species. Finally, evaluation of the physiology of plants in saline soils in field conditions will also be an important aspect of future research, although field research will require detailed soil characterisation, as salinity can be variable, even across short distances.

## Figures and Tables

**Table 1:** Ion concentrations, salts used, and osmotic potential of the non-saline control and four salt treatments’ (100 mM Na^+^ salts (without Cl^−^), 100 mM Cl^−^ salts (without Na^+^), 100mM NaCl, and 100 mM high-cation negative control (no Na^+^, no Cl^−^)) nutrient solutions.

Treatments	Ψπ (MPa)	Ion Concentrations (mM)									
		Na^+^	Cl^−^	K^+^	Ca^2+^	Mg^2+^	SO_4_^2−^	NO_3_^−^	SiO_3_^−^	NH_4_^+^	H_2_PO_4_^−^
Control	−0.05	0.2	0.05	5.0	5.0	0.4	5.4	4.4	0.1	0.6	0.2
NaCl (100 mM)	−0.49	100.2	100.05	5.0	5.0	0.4	5.4	4.4	0.1	0.6	0.2
Na^+^ (100 mM, without Cl^−^): 33.33 mM Na_2_SO_4_ + 33.33 mM NaNO_3_	−0.41	100.2	0.05	5.0	5.0	0.4	38.7	37.7	0.1	0.6	0.2
Cl^−^ (100 mM, without Na^+^): 16.67 mM CaCl_2_ + 16.67 mM MgCl_2_ + 33.33 mM KCl	−0.41	0.2	100.05	38.3	16.7	17.1	5.4	4.4	0.1	0.6	0.2
High-cation negative control (equivalent to Cl^−^ treatment): 10.0 mM CaSO_4_ + 6.67 mM Ca(NO_3_)_2_ + 16.67 mM MgSO_4_ + 16.67 mM K_2_SO_4_	−0.43	0.2	0.05	71.6	28.3	17.1	65.3	11.1	0.1	0.6	0.2

For the basal nutrient solution, the macronutrient concentrations are shown in the table (Control). The micronutrients in all solutions were (µM): 100 Fe–sequestrene, 25 HBO_3_^2−^, 2.0 mM Mn^2+^, 2.0 Zn^2+^, 0.50 Cu^2+^, 0.50 MoO_4_^2−^, 1.0 Ni^2+^. The solution was buffered with 1.0 mM MES (2-(*N*-morpholino) ethanesulfonic acid) and adjusted to pH 6.5 using KOH.

**Table 2:** Reproductive attributes of soybean, mungbean, cowpea, and common bean grown in control (non-saline), 100 mM NaCl, 100 mM Na^+^ (without Cl^−^), 100 mM Cl^−^ (without Na^+^), and high-cation negative control (K^+^, Mg^2+^, and Ca^2+^ equivalent to those in the 100 mM Cl^−^ treatment) treatments. The salts used in the various treatments are given in Table 1. Treatments were imposed on 13-day-old plants. The data are for mature pods (pod walls and seeds), mature seed dry mass, and the number of mature seeds per plant at 57 days. The values are means ± SE (*n* = 4). The least significant differences (LSDs) for treatment means within each species, treatment, and species × treatment interaction are given at the bottom of each data column (*p* = 0.05). The probability levels for two-way ANOVA were used to compare species (S), treatment (T), and species × treatment interaction (S × T) effects (* *p* < 0.05, ** *p* < 0.01, *** *p* < 0.001, and n.s. = not significant).

Species	Treatment	Pod Dry Mass (g)	% of Control	Seed Dry Mass (g)	% of Control	Seed Number	% of Control
Soybean	Control	21.1 ± 0.8	-	10.0 ± 0.5		101 ± 5	-
	NaCl	10.9 ± 1.6	52	6.2 ± 0.9	62	41 ± 8	41
	Na^+^ salts	5.2 ± 2.9	25	3.0 ± 1.6	30	49 ± 5	48
	Cl^−^ salts	15.6 ± 3.1	74	6.3 ± 1.5	63	65 ± 6	64
	High cation	15.5 ± 1.4	73	10.4 ± 1.6	104	84 ± 9	83
	LSD (5%)	6.4 ***	-	3.9 **	-	53 *	-
Mungbean	Control	15.5 ± 1.0		10.9 ± 0.9		151 ± 10	-
	NaCl	0.4 ± 0.2	3	0.2 ± 0.1	2	3 ± 1	2
	Na^+^ salts	1.8 ± 0.8	12	1.3 ± 0.6	12	18 ± 6	12
	Cl^−^ salts	1.1 ± 0.2	7	0.3 ± 0.04	3	6.0 ± 2	4
	High cation	6.1 ± 1.3	39	4.3 ± 1.1	39	67 ± 10	44
	LSD (5%)	3.4 ***	-	2.9 ***	-	47 ***	-
Cowpea	Control	26.2 ± 2.5	-	22.0 ± 1.6	-	372 ± 12	-
	NaCl	6.3 ± 1.0	24	4.7 ± 1.3	21	71 ± 15	19
	Na^+^ salts	11.3 ± 2.0	43	9.7 ± 1.6	44	166 ± 11	45
	Cl^−^ salts	12.7 ± 2.0	48	10.6 ± 1.6	48	167 ± 12	45
	High cation	18.6 ± 0.9	71	15.6 ± 0.7	71	240 ± 14	64
	LSD (5%)	5.3 ***	-	4.2 ***	-	89 ***	-
Common bean	Control	17.9 ± 3.6		13.5 ± 3.3		94 ± 12	
	NaCl	1.4 ± 0.7	8	0.6 ± 0.1	4	6 ± 2	6
	Na^+^ salts	9.4 ± 0.9	52	6.5 ± 1.0	48	46 ± 11	49
	Cl^−^ salts	4.8 ± 2.8	27	2.8 ± 1.9	21	56 ± 11	59
	High cation	8.2 ± 2.5	46	5.5 ± 1.8	41	54 ± 11	57
	LSD (5%)	7.6 **	-	5.9 **	-	59 *	-
LSD (5%)	S	2.5 ***	-	1.8 ***	-	31 ***	-
	T	2.8 ***	-	2.1 ***	-	35 ***	-
	S × T	5.3 ***	-	4.1 *	-	71 ***	-

**Table 3:** Leaf sap osmotic potential (Ψπ_sap_) measured on the second-youngest fully expanded leaves (leaf lamina) of soybean, mungbean, cowpea, and common bean grown in control (non-saline), 100 mM NaCl, 100 mM Na^+^ (without Cl^−^), 100 mM Cl^−^ (without Na^+^), and high-cation negative control (K^+^, Mg^2+^, and Ca^2+^ equivalent to those in the 100 mM Cl^−^ treatment) treatment. The salts used in the various treatments are given in Table 1. Treatments were imposed on 13-day-old plants and sampled after 15 (vegetative stage), 36 (podding stage), and 57 (pod-filling stage) days of treatment. Ψπ_sap_ was measured by using a freezing-point depression osmometer. The change in Ψπ_sap_ = Control–Treatment. The values are means ± SE (*n* = 4). The least significant differences (LSDs) for treatment means within each species, treatments, and species × treatment interaction are given at the bottom of each data column (*p* = 0.05). The probability levels for two-way ANOVA were used to compare species (S), treatment (T), and species × treatment interaction (S × T) effects (* *p* < 0.05, ** *p* < 0.01, *** *p* < 0.001, and n.s. = not significant).

Species	Treatment	Pod-Filling Stage					
		Ψπ_sap_ (MPa)	Change in Ψπ_sap_ (MPa)	Ψπ_sap_ (MPa)	Change in Ψπ_sap_ (MPa)	Ψπ_sap_ (MPa)	Change in Ψπ_sap_ (MPa)
Soybean	Control	−1.01 ± 0.10	-	−0.94 ± 0.05	-	−1.11 ± 0.11	-
	NaCl	−1.63 ± 0.17	0.62	−1.19 ± 0.04	0.25	−1.35 ± 0.07	0.25
	Na^+^ salts	−1.10 ± 0.05	0.09	−1.14 ± 0.10	0.20	−1.76 ± 0.11	0.65
	Cl^−^ salts	−1.18 ± 0.08	0.17	−1.13 ± 0.01	0.19	−1.39 ± 0.04	0.28
	High cation	−1.10 ± 0.10	0.09	−1.15 ± 0.11	0.21	−1.52 ± 0.10	0.41
	LSD (5%)	0.32 ***	-	n.s.	-	0.27 **	-
Mungbean	Control	−0.76 ± 0.05	-	−0.77 ± 0.05	-	−0.94 ± 0.05	-
	NaCl	−1.88 ± 0.25	1.12	−1.24 ± 0.01	0.46	-	-
	Na^+^ salts	−0.91 ± 0.08	0.15	−1.01 ± 0.07	0.23	−1.77 ± 0.04	0.82
	Cl^−^ salts	−2.05 ± 0.11	1.29	−1.62 ± 0.01	0.85	-	-
	High cation	−1.11 ± 0.02	0.35	−1.17 ± 0.09	0.39	−1.33 ± 0.07	0.39
	LSD (5%)	0.39 ***	-	0.15 ***	-	0.5 ***	-
Cowpea	Control	−0.83 ± 0.03	-	−0.85 ± 0.08	-	−1.00 ± 0.11	-
	NaCl	−1.12 ± 0.03	0.29	−1.07 ± 0.05	0.22	−1.08 ± 0.02	0.08
	Na^+^ salts	−1.10 ± 0.06	0.27	−1.13 ± 0.08	0.28	−1.35 ± 0.23	0.34
	Cl^−^ salts	−1.12 ± 0.06	0.29	−1.03 ± 0.06	0.17	−1.25 ± 0.07	0.25
	High cation	−1.13 ± 0.07	0.30	−1.00 ± 0.04	0.14	−1.05 ± 0.05	0.04
	LSD (5%)	0.16 **	-	n.s.	-	n.s.	-
Common bean	Control	−0.98 ± 0.02	-	−0.75 ± 0.03	-	−0.95 ± 0.16	-
	NaCl	−1.25 ± 0.06	0.27	−1.36 ± 0.11	0.61	−1.75 ± 0.04	0.80
	Na^+^ salts	−1.28 ± 0.10	0.30	−1.11 ± 0.06	0.36	−1.38 ± 0.09	0.43
	Cl^−^ salts	−1.20 ± 0.03	0.22	−1.11 ± 0.09	0.36	−1.74 ± 0.10	0.79
	High cation	−1.10 ± 0.03	0.12	−1.04 ± 0.04	0.39	−1.12 ± 0.05	0.17
	LSD (5%)	0.16 *	-	0.22 ***		0.37 *	
LSD (5%)	S	0.11 ***		0.08 ***		0.36 **	
	T	0.13 ***		0.09 ***		0.40 ***	
	S × T	0.24 ***		0.18 ***		0.80 **	

## Data Availability

The data presented in this study are available in the article and its Supplementary Material.

## Supplementary Materials

Supplementary Materials can be found at https://www.mdpi.com/1422-0067/22/4/1909/s1. Figure S1: Dry mass of lamina, petioles, and stems ; Figure S2: Na^+^ concentration in shoots and roots; Figure S3: Tissue Na^+^ concentration in green petioles, and green stems; Figure S4: Cl^−^ concentration in shoots and roots; Figure S5: Tissue Cl^−^ concentration in green petioles, and green stems; Figure S6: K^+^ concentration in shoots and roots; Figure S7: Tissue K^+^ concentration in green petioles, and green stems; Figure S8: K^+^/Na^+^ ratios in shoots nd roots; Figure S9: Tissue K^+^/ Na^+^ ratios in green petioles and green stems; Figure S10: Tissue Na^+^ and Cl^−^ concentration in flowers, mature pod walls, and mature seeds; Figure S11: Sucrose and pinitol; Figure S12: Scatter plots of shoot dry mass against shoot Na^+^ concentration; Figure S13: Scatter plots of shoot dry mass against shoot Cl^−^ concentration; Figure S14: Scatter plots of shoot dry mass against shoot K^+^ concentration; Figure S15: Scatter plots of shoot dry mass against shoot K^+^/Na^+^ ratio; Figure S16: Scatter plots of net photosynthesis against stomatal conductance and intercellular CO_2_ concentration; Figure S17: Scatter plots of stomatal conductance against lamina Na^+^ concentration; Figure S18: Scatter plots of transpiration rate against lamina Na^+^, Cl^−^, ans K^+^ concentrations; Table S1: Days to first leaf damage and days to first flower; Table S2: Na^+^, Cl^−^, K^+^, and K^+^/Na^+^ in lamina, petiole, stems and roots; Table S3: K^+^/Na^+^ in lamina, petiole, stems and roots; Table S4: K^+^ concentration and K^+^/Na^+^ ratio in flowers, pod walls, and seeds; Table S5: Leaf water content measured on the second youngest fully expanded leaves; Table S6: Osmotic potential of the external solution bathing the roots; Table S7: Summary of toxic, marginal levels, and adequate levels of ion concentration.
