# Supplementary material for: Na+ and/or Cl− Toxicities Determine Salt Sensitivity in Soybean (Glycine max (L.) Merr.), Mungbean (Vigna radiata (L.) R. Wilczek), Cowpea (Vigna unguiculata (L.) Walp.), and Common Bean (Phaseolus vulgaris L.)
Source: Int J Mol Sci. 2021 Feb 14;22(4):1909. doi: 10.3390/ijms22041909 (PMC7918652; doi:10.3390/ijms22041909)
Supplement: Supplementary file 1 [file ijms-22-01909-s001.pdf]

International Journal of
Molecular Sciences

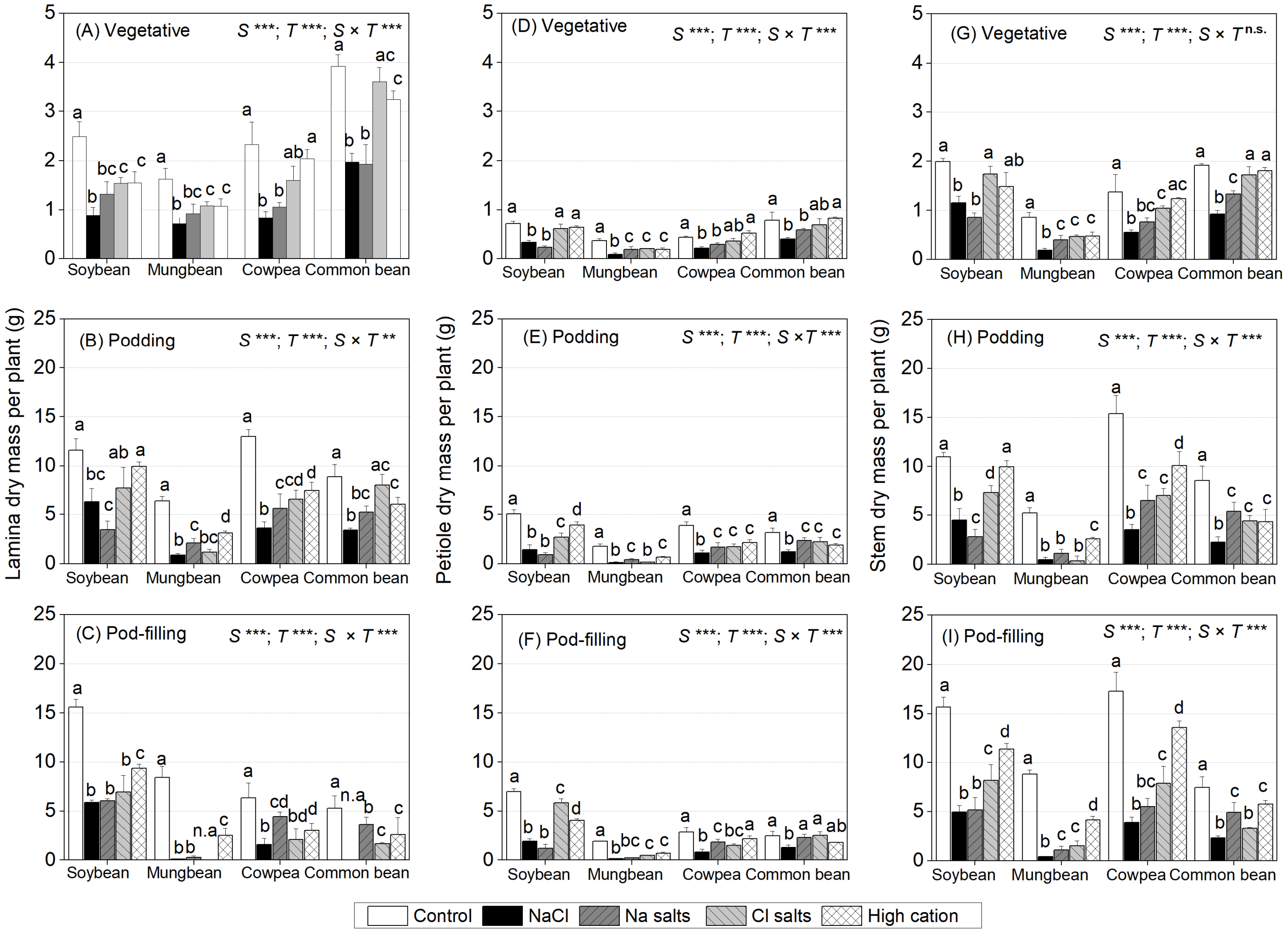

Figure S1. Dry mass of lamina, petioles, and stems per plant of soybean, mungbean, cowpea and common bean grown in control (non-saline), 100 mM NaCl, 100 mM Na^+ (without Cl^-), 100 mM Cl^- (without Na^+) and high cation negative control (K^+, Mg^{2+} and Ca^{2+} equivalent to those in the 100 mM Cl^- salts) treatments. Salts used in the various treatments are given in Table 1. Treatments were imposed on 13 day-old plants and sampled after (**A**,**D**,**G**) 15 (vegetative stage), (**B**,**E**,**H**) 36 (podding stage), and (**C**,**F**,**I**) 57 (pod-filling stage) days of treatment. Values are means ± SE (n = 4). Significant differences for treatment means within each species are indicated by different letters (a–d) (p = 0.05). The probability levels for two-way ANOVA were used to compare species (S), treatment (T) and species × treatment interaction ($S \times T$) effects (** $p < 0.01$, *** $p < 0.001$, and n.s. = not significant). Note: Mungbean subjected to Cl^- (without Na^+) and common bean subjected to the NaCl treatment did not have enough green leaf lamina at the pod–filling stage for ion analysis, as indicated by n.a (data not available); The axis scales for lamina, petiole, and stems dry mass at the (**A**,**D**,**G**) vegetative stage differs from the others.

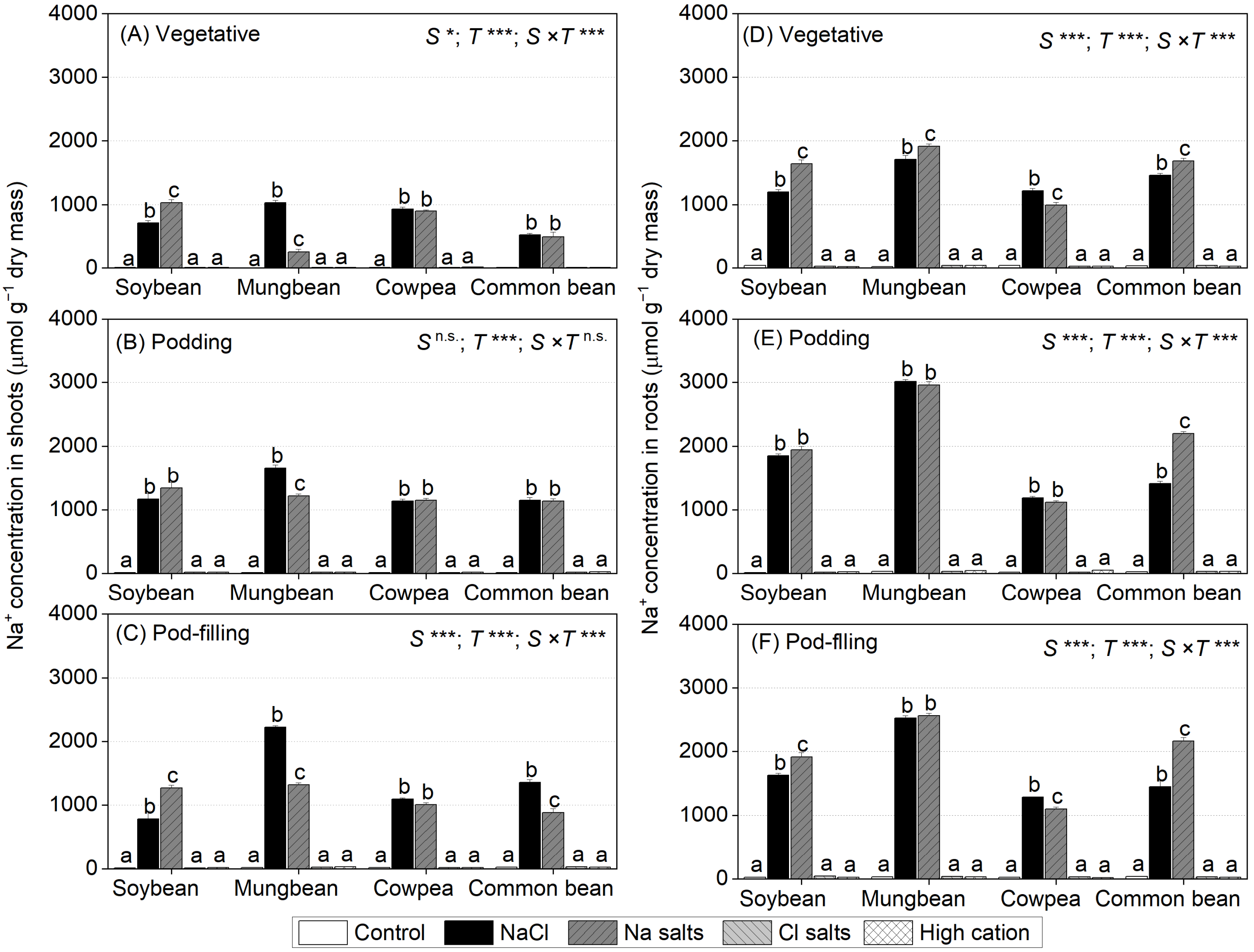

Figure S2. Na^+ concentration in shoots (stems, petioles and lamina) and roots of soybean, mungbean, cowpea and common bean grown in control (non-saline), 100 mM NaCl, 100 mM Na^+ (without Cl^-), 100 mM Cl^- (without Na^+), and high cation negative control (K^+, Mg^{2+} and Ca^{2+} equivalent to those in the 100 mM Cl^- treatment) treatments. Salts used in the various treatments are given in Table 1. Treatments were imposed on 13 day-old plants and sampled after (**A**,**D**) 15 (vegetative stage), (**B**,**E**) 36 (podding stage) and (**C**,**F**) 57 (pod-filling stage) days of treatment. Values are means ± SE (n = 4). Significant differences for treatment means within each species are indicated by different letters (a–c) ($p = 0.05$). The probability levels for two-way ANOVA were used to compare species (*S*), treatment (*T*), and species × treatment interaction (*S* × *T*) effects (* $p < 0.05$, *** $p < 0.001$, and n.s. = not significant).

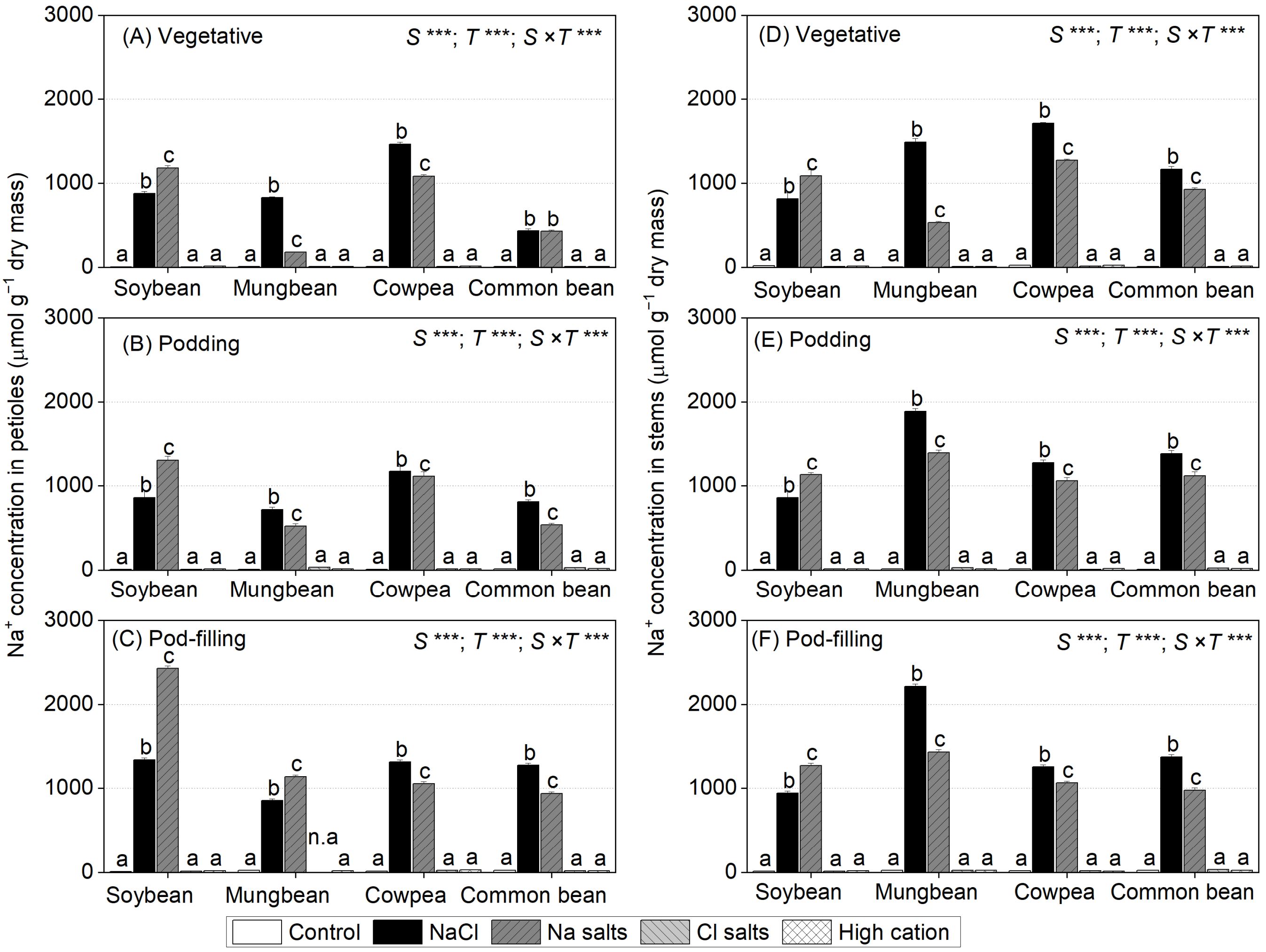

Figure S3. Tissue Na^+ concentration in green petioles, and green stems of soybean, mungbean, cowpea, and common bean grown in control (non-saline), 100 mM NaCl, 100 mM Na^+ (without Cl^-), 100 mM Cl^- (without Na^+) and high cation negative control (K^+, Mg^{2+} and Ca^{2+} equivalent to those in the 100 mM Cl^- treatment) treatments. Salts used in the various treatments are given in Table 1. Treatments were imposed on 13 day-old plants and sampled after (**A**,**D**) 15 (vegetative stage), (**B**,**E**) 36 (podding stage), and (**C**,**F**) 57 (pod-filling stage) days of treatment. Values are means ± SE ($n = 4$). Significant differences for treatment means within each species are indicated by different letters (a–c) ($p = 0.05$). The probability levels for two–way ANOVA were used to compare species (*S*), treatment (*T*) and species × treatment interaction (*S* × *T*) effects (** $p < 0.01$, *** $p < 0.001$, and n.s. = not significant). Note: Mungbean subjected to Cl^- (without Na^+) did not have enough green petioles at the pod–filling stage for ion analysis, as indicated by n.a.

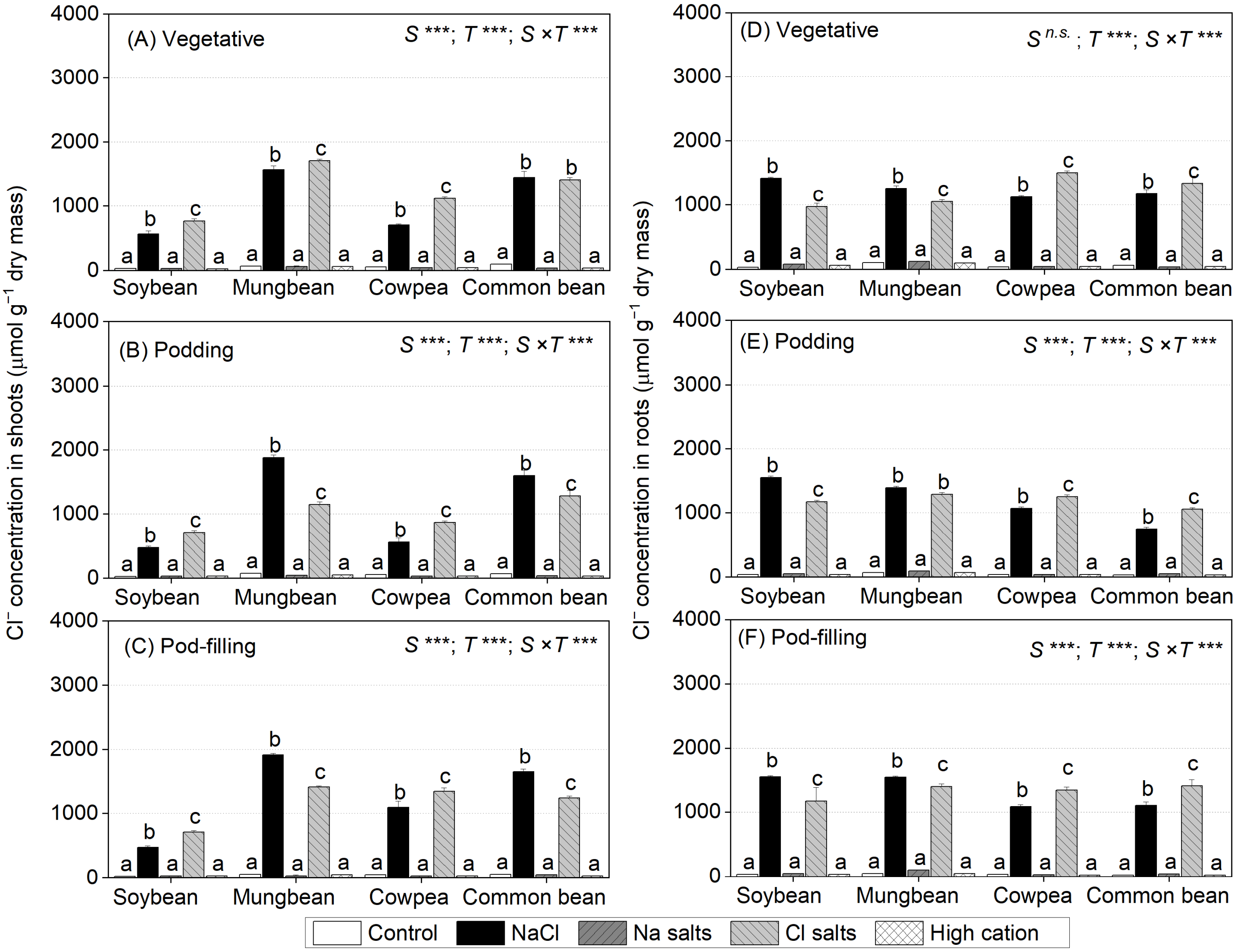

Figure S4. Cl^- concentration in shoots (stems, petioles and lamina) and roots of soybean, mungbean, cowpea and common bean grown in control (non-saline), 100 mM NaCl, 100 mM Na^+ (without Cl^-), 100 mM Cl^- (without Na^+), and high cation negative control (K^+, Mg^{2+} and Ca^{2+} equivalent to those in the 100 mM Cl^- treatment) treatments. Salts used in the various treatments are given in Table 1. Treatments were imposed on 13 day-old plants and sampled after (**A**,**D**) 15 (vegetative stage), (**B**,**E**) 36 (podding stage), and (**C**,**F**) 57 (pod-filling stage) days of treatment. Values are means ± SE ($n = 4$). Significant differences for treatment means within each species are indicated by different letters (a–c) ($p = 0.05$). The probability levels for two–way ANOVA were used to compare species (S), treatment (T) and species × treatment interaction ($S \times T$) effects (* $p < 0.05$, *** $p < 0.001$, and *n.s.* = not significant).

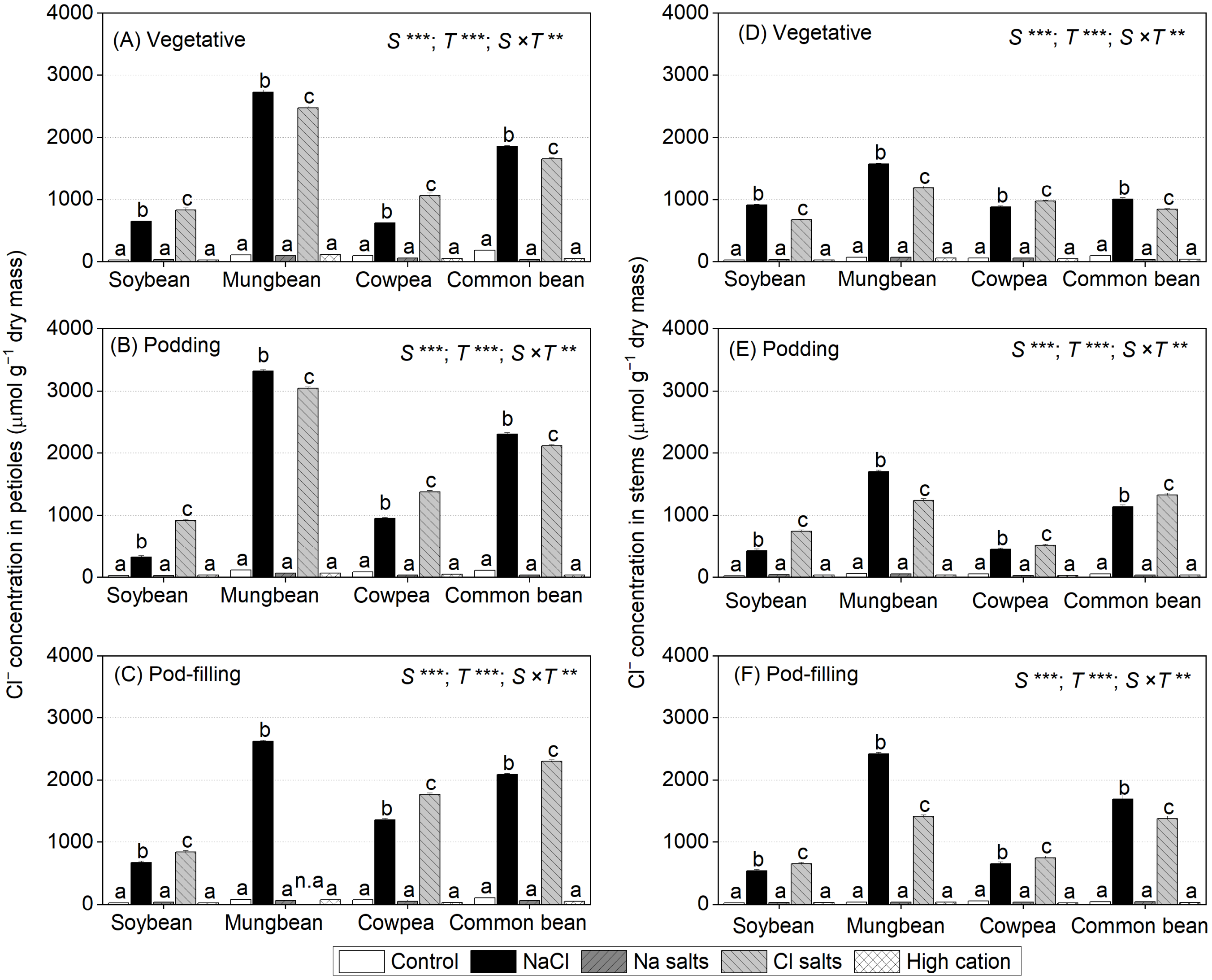

Figure S5. Tissue Cl^- concentration in green petioles, and green stems of soybean, mungbean, cowpea and common bean grown in control (non-saline), 100 mM NaCl, 100 mM Na^+ (without Cl^-), 100 mM Cl^- (without Na^+) and high cation negative control (K^+, Mg^{2+} and Ca^{2+} equivalent to those in the 100 mM Cl^- treatment) treatments. Salts used in the various treatments are given in Table 1. Treatments were imposed on 13 day-old plants and sampled after (**A**,**D**) 15 (vegetative stage), (**B**,**E**) 36 (podding stage) and (**C**,**F**) 57 (pod-filling stage) days of treatment. Values are means ± SE (n = 4). Significant differences for treatment means within each species are indicated by different letters (a–c) ($p = 0.05$). The probability levels for two-way ANOVA were used to compare species (S), treatment (T) and species × treatment interaction ($S \times T$) effects (** $p < 0.01$, and *** $p < 0.001$). Note: Mungbean subjected to Cl^- (without Na^+) did not have enough green petioles at the pod–filling stage for ion analysis, as indicated by n.a.

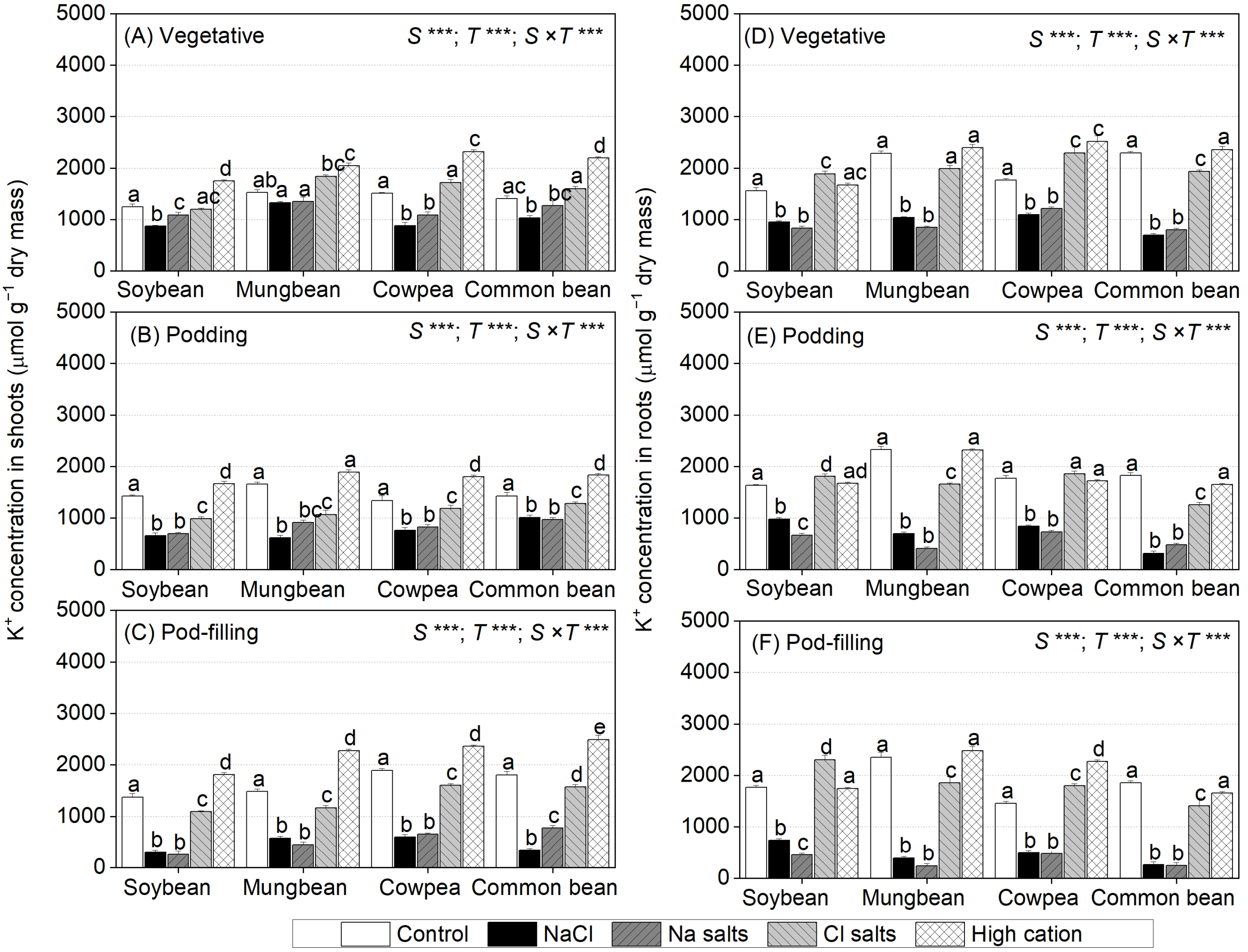

Figure S6. K^+ concentration in shoots (stems, petioles and lamina) and roots of soybean, mungbean, cowpea and common bean grown in control (non-saline), 100 mM NaCl, 100 mM Na^+ (without Cl^-), 100 mM Cl^- (without Na^+) and high cation negative control (K^+, Mg^{2+} and Ca^{2+} equivalent to those in the 100 mM Cl^- treatment) treatments. Salts used in the various treatments are given in Table 1. Treatments were imposed on 13 day-old plants and sampled after (**A**,**D**) 15 (vegetative stage), (**B**,**C**) 36 (podding stage) and (**C**,**F**) 57 (pod-filling stage) days of treatment. Values are means ± SE (n = 4). Significant differences for treatment means within each species are indicated by different letters (a–d) (p = 0.05). The probability levels for two-way ANOVA were used to compare species (S), treatment (T) and species × treatment interaction ($S \times T$) effects *** $p < 0.001$).

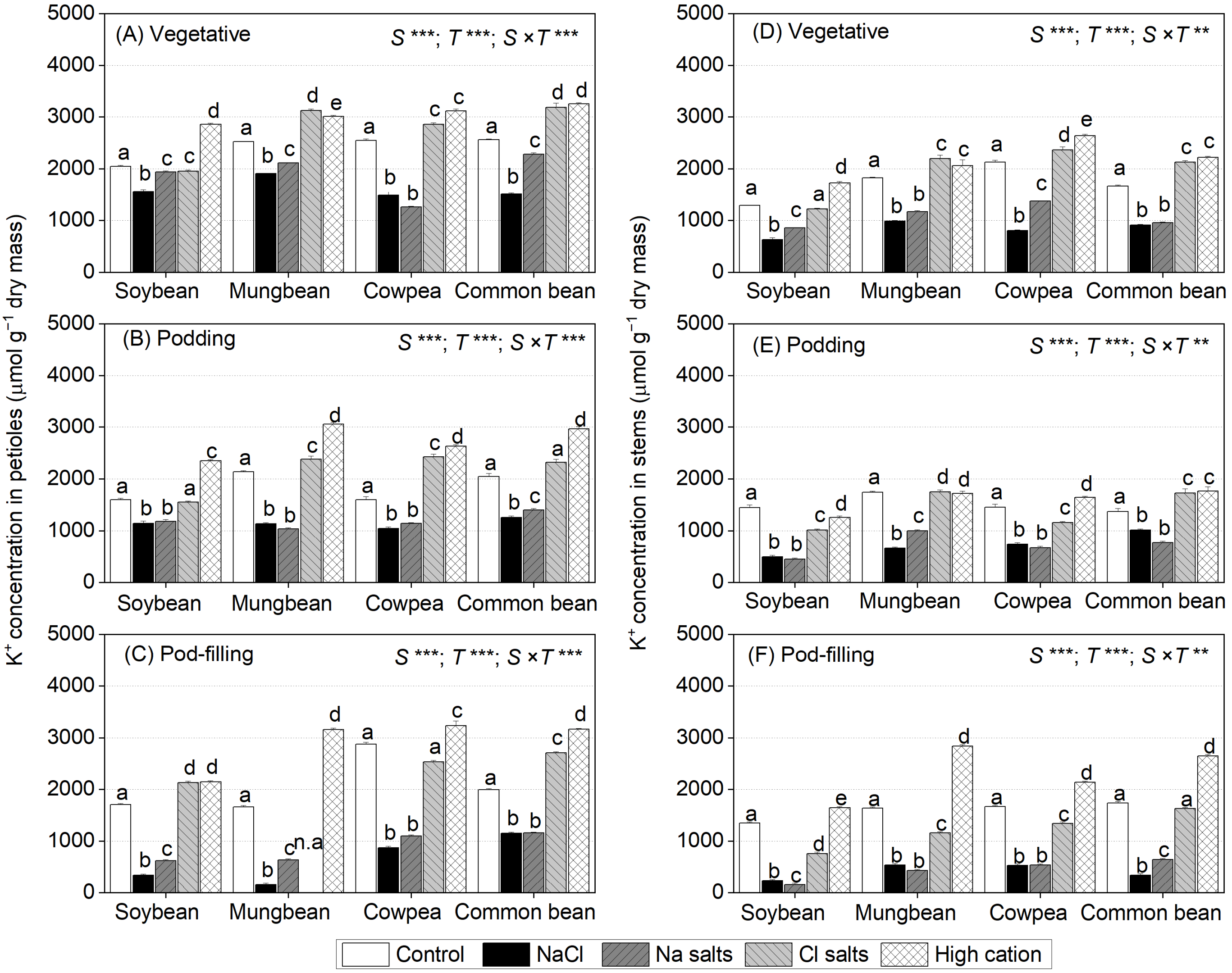

Figure S7. Tissue K^+ concentration in green petioles, and green stems of soybean, mungbean, cowpea, and common bean, grown in control (non-saline), 100 mM NaCl, 100 mM Na^+ (without Cl^-), 100 mM Cl^- (without Na^+), and high cations negative control (K^+, Mg^{2+} and Ca^{2+} equivalent to those in the 100 mM Cl^- treatment) treatments. Salts used in the various treatments are given in Table 1. Treatments were imposed on 13 day-old plants and sampled after (**A**,**D**) 15 (vegetative stage;), (**B**,**E**) 36 (podding stage) and (**C**,**F**) 57 (pod–filling stage) days of treatment. Values are means ± SE ($n = 4$). Significant differences for treatment means within each species are indicated by different letters (a–d) ($p = 0.05$). The probability levels for two-way ANOVA was used to compare species (*S*), treatment (*T*) and species × treatment interaction (*S* × *T*) effects (** $p < 0.01$ and *** $p < 0.001$). Mungbean subjected to Cl^- (without Na^+) did not have enough green petioles at the pod–filling stage for ion analysis, as indicated by n.a.

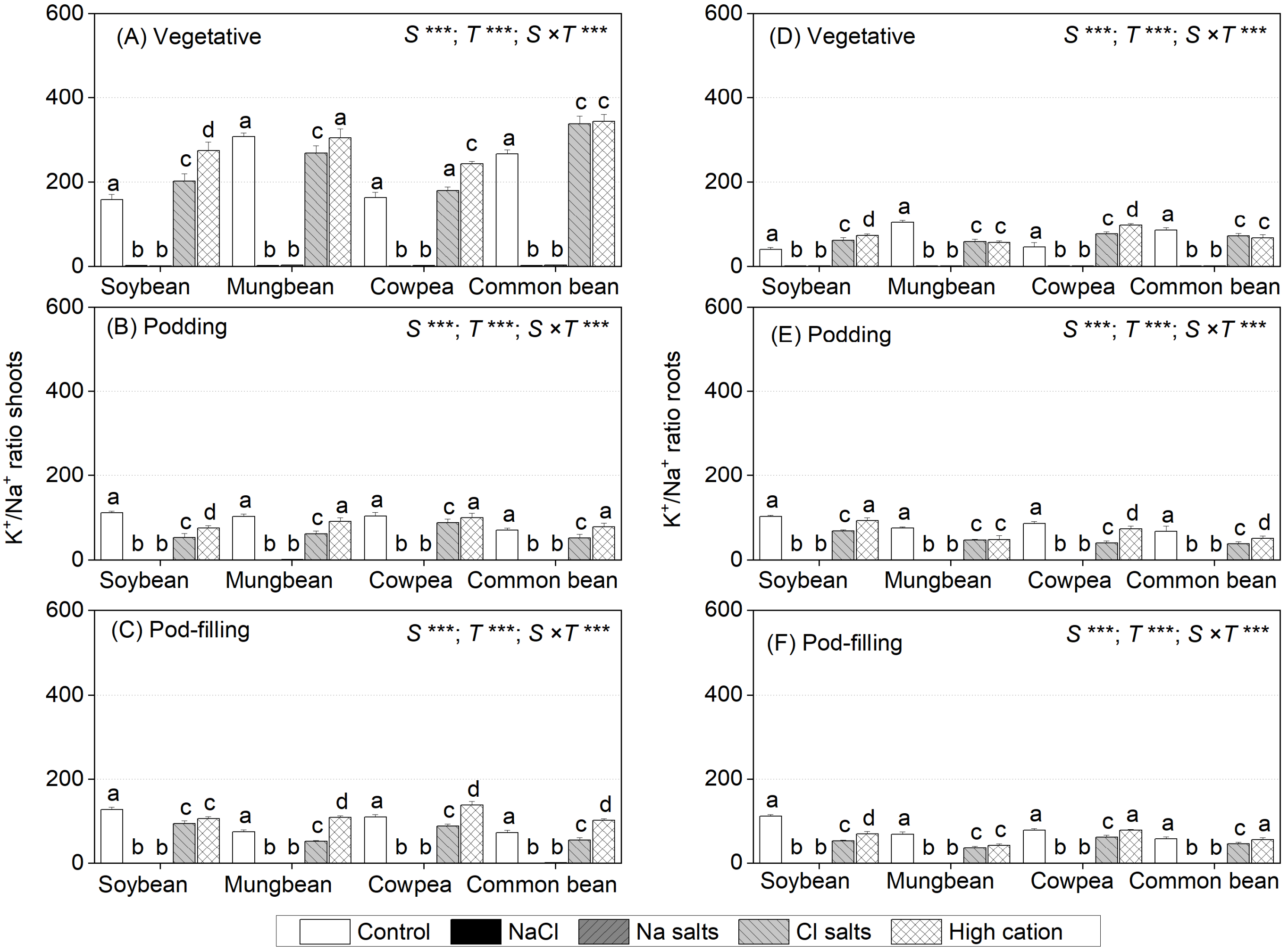

Figure S8. K^+/Na^+ ratios in shoots (stems, petioles and lamina) and roots of soybean, mungbean, cowpea and common bean grown in control (non-saline), 100 mM NaCl, 100 mM Na^+ (without Cl^-), 100 mM Cl^- (without Na^+) and high cation negative control (K^+, Mg^{2+} and Ca^{2+} equivalent to those in the 100 mM Cl^- treatment) treatments. Salts used in the various treatments are given in Table 1. Treatments were imposed on 13 day-old plants and sampled after (**A**,**D**) 15 (vegetative stage), (**B**,**E**) 36 (podding stage) and (**C**,**F**) 57 (pod-filling stage) days of treatment. Values are means ± SE ($n = 4$). Significant differences for treatment means within each species are indicated by different letters (a–d) ($p = 0.05$). The probability levels for two-way ANOVA were used to compare specie (*S*), treatment (*T*) and species × treatment interaction (*S* × *T*) effects (*** $p < 0.001$). Note: Data for the K^+/Na^+ ratios in the shoots and roots of the four legume species subjected to NaCl and Na^+ salts are also presented in Table S3.

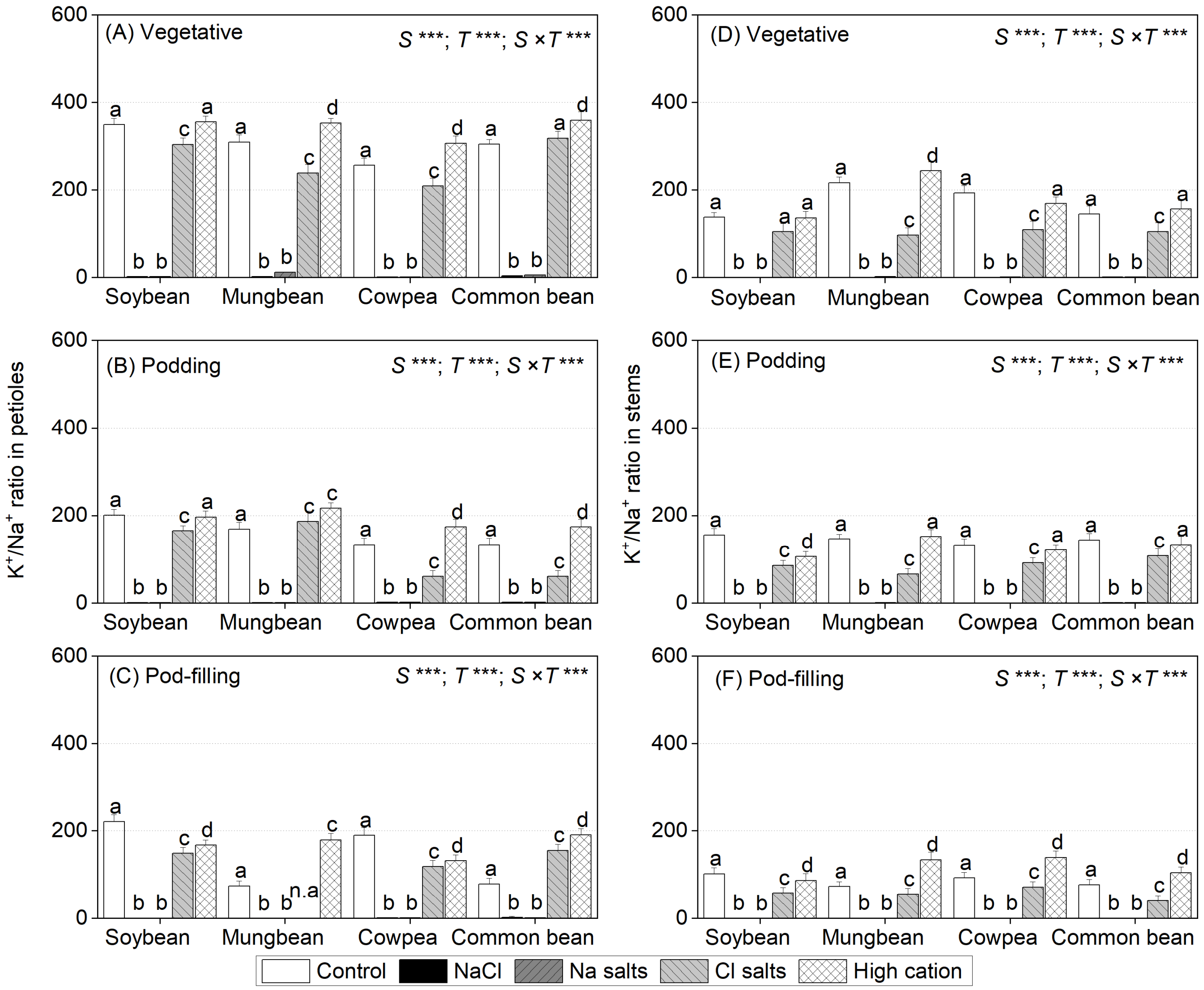

Figure S9. Tissue K^+/ Na^+ ratios in green petioles and green stems of soybean, mungbean, cowpea and common bean grown in control (non-saline), 100 mM NaCl, 100 mM Na^+ (without Cl^-), 100 mM Cl^- (without Na^+) and high cation negative control (K^+, Mg^{2+} and Ca^{2+} equivalent to those in the 100 mM Cl^-treatment) treatments. Salts used in the various treatments are given in Table 1. Treatments were imposed on 13 day-old plants and sampled after (**A**,**D**) 15 (vegetative stage), (**B**,**E**) 36 (podding stage) and (**C**,**F**) 57 (pod-filling stage) days of treatment. Values are means ± SE (n = 4). Significant differences for treatment means within each species are indicated by different letters (a–d) (p = 0.05). The probability levels for two-way ANOVA were used to compare species (S), treatment (T) and species × treatment interaction ($S \times T$) effects (*** $p < 0.001$). Note: Data for the K^+/Na^+ ratios in the shoots and roots of the four legume species subjected to NaCl and Na^+ salts are also presented in Table S3. Mungbean subjected to Cl^- (without Na^+) did not have enough green petioles at the pod–filling stage for ion analysis, as indicated by n.a.

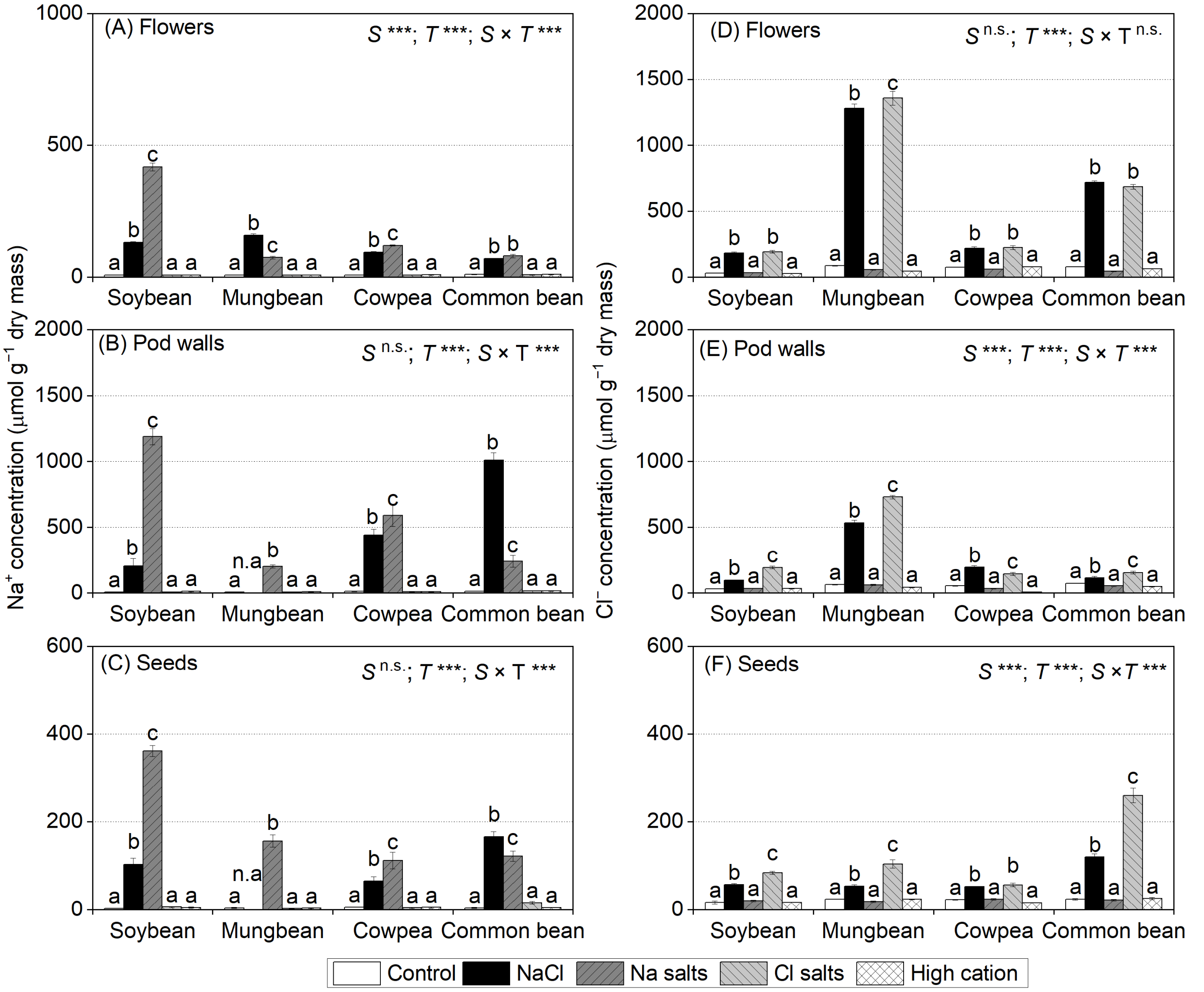

Figure S10. Tissue Na^+ and Cl^- concentration in (**A**,**D**) flowers, (**B**,**F**) mature pod walls, and (C,**H**) mature seeds of soybean, mungbean, cowpea, and common bean grown in control (non–saline), 100 mM NaCl, 100 mM Na^+ (without Cl^-), 100 mM Cl^- (without Na^+) and high cation negative control (K^+, Mg^{2+} and Ca^{2+} equivalent to those in the 100 mM Cl^-) treatments. Salts used in the various treatments are given in Table 1. Treatments were imposed on 13 day-old plants and sampled after 57 (pod-filling stage) days of treatment. Values are means ± SE (n = 4). Significant difference for treatment means within each species are indicated by different letters (a–c) (p = 0.05). The probability levels for two–way ANOVA were used to compare species (S), treatment (T) and species × treatment interaction ($S \times T$) effects (*** $p < 0.001$ and *n.s.* = not significant). Note: Data for the K^+ and K^+/Na^+ ratio in the shoots and roots of the four legume species subjected to NaCl and Na^+ salts are also presented in Table S4. Mungbean subjected to Cl^- (without Na^+) did not have enough green petioles at the pod–filling stage for ion analysis, as indicated by n.a.

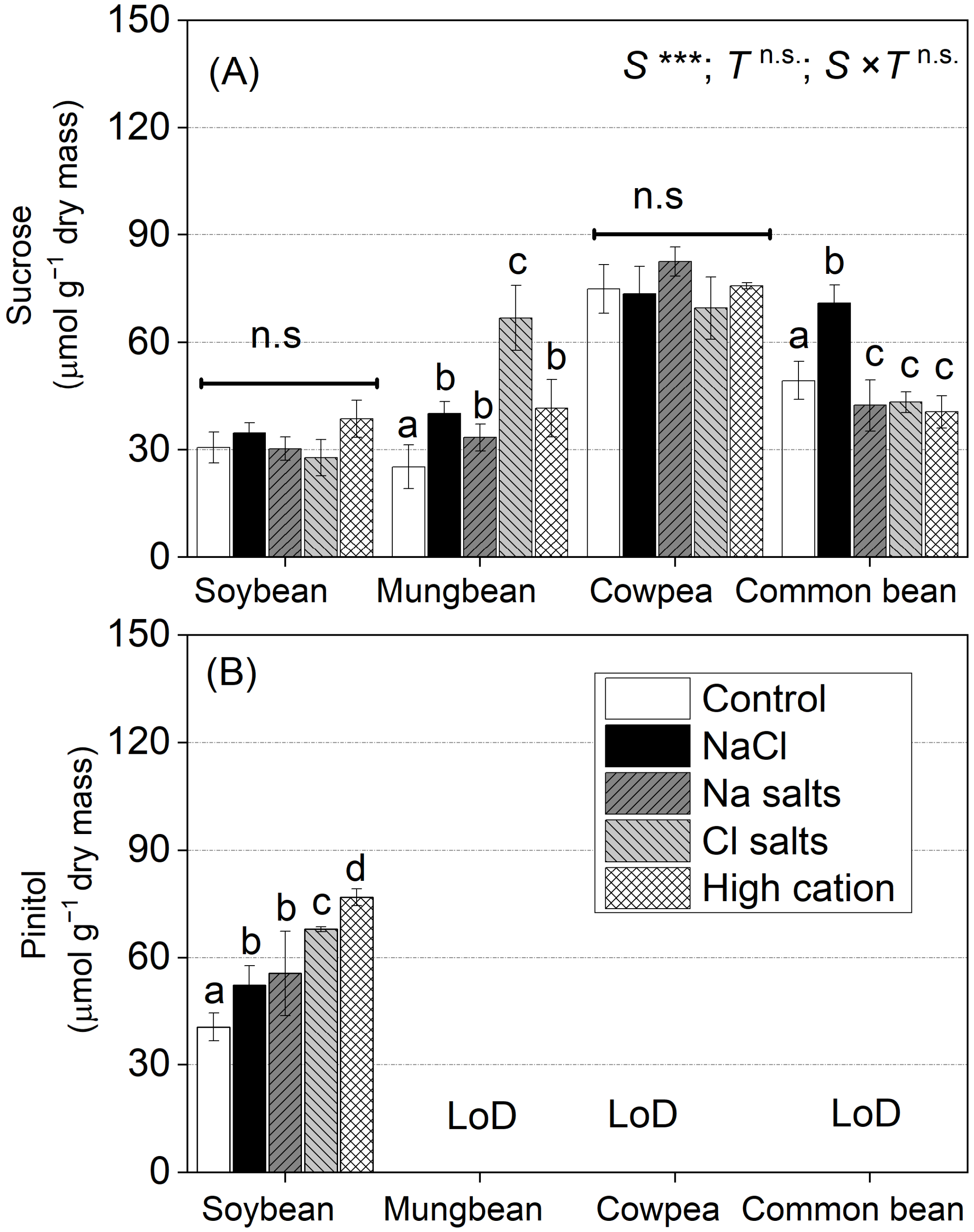

Figure S11. Sucrose (**A**) and pinitol (**B**) of soybean, mungbean, cowpea, and common bean grown in control (non–saline), 100 mM NaCl, 100 mM Na^+ (without Cl^-), 100 mM Cl^- (without Na^+), and high cation negative control (K^+, Mg^{2+} and Ca^{2+} equivalent to those in the 100 mM Cl^- treatment) treatments. Salts used in the various treatments are given in Table 1. Treatments were imposed on 13 day-old plants, with sucrose and pinitol measured on the lamina of the second youngest leaf sampled at the vegetative stage. Values are means ± SE ($n = 4$). Significant differences for treatment means within each species are indicated by different letters (a–d) ($p = 0.05$). The probability levels for two–way ANOVA were used to compare species (*S*), treatment (*T*) and species × treatment interaction ($S \times T$) effects (*** $p < 0.001$ and n.s. = not significant). Note: LoD = limit of detection.

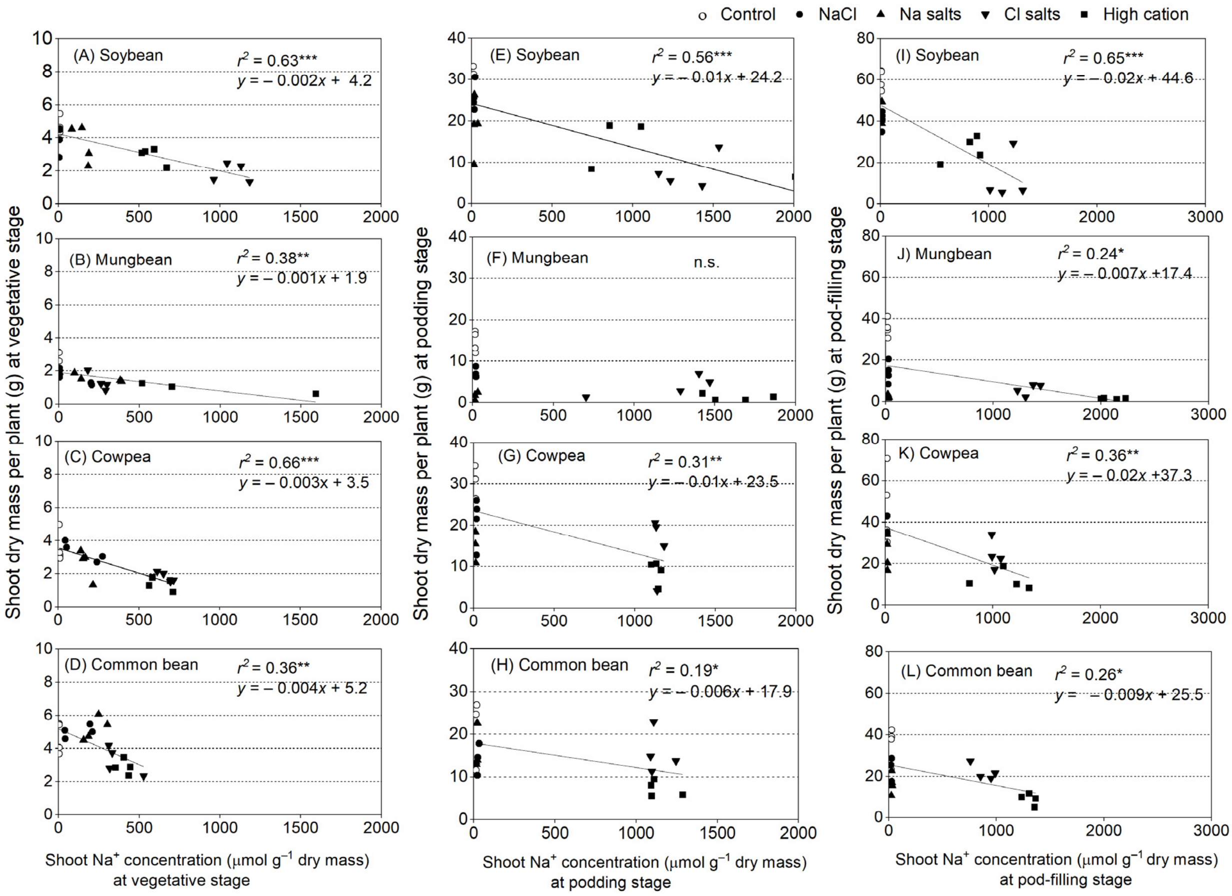

Figure S12. Scatter plots of shoot dry mass against shoot Na^+ concentration (stems, petioles, and lamina) of soybean, mungbean, cowpea , and common bean grown in control (non-saline; open circles), 100 mM NaCl (solid circles), 100 mM Na^+ (without Cl^-) (solid, upward triangles), 100 mM Cl^- (without Na^+) (solid, downward triangles) and high cation negative control (solid squares) (K^+, Mg^{2+} and Ca^{2+} equivalent to those in the 100 mM Cl^- treatment) treatments. Salts used in the various treatments are given in Table 1. Treatments were imposed on 13-day-old plants and sampled after (**A–D**) 15 (vegetative stage), (**E–H**) 36 (podding stage), and (**I–L**) 57 (pod–filling stage) days of treatment. Each value is an individual replicate and each replicate is one plant growing in a different pot. * significant at $p < 0.05$, ** significant at $p < 0.01$, *** significant at $p < 0.001$, and n.s. = not significant. Note: the axis scales differ for (**A–D**) vegetative stage, (**E–H**) podding stage, and (**I–L**) reproductive stage.

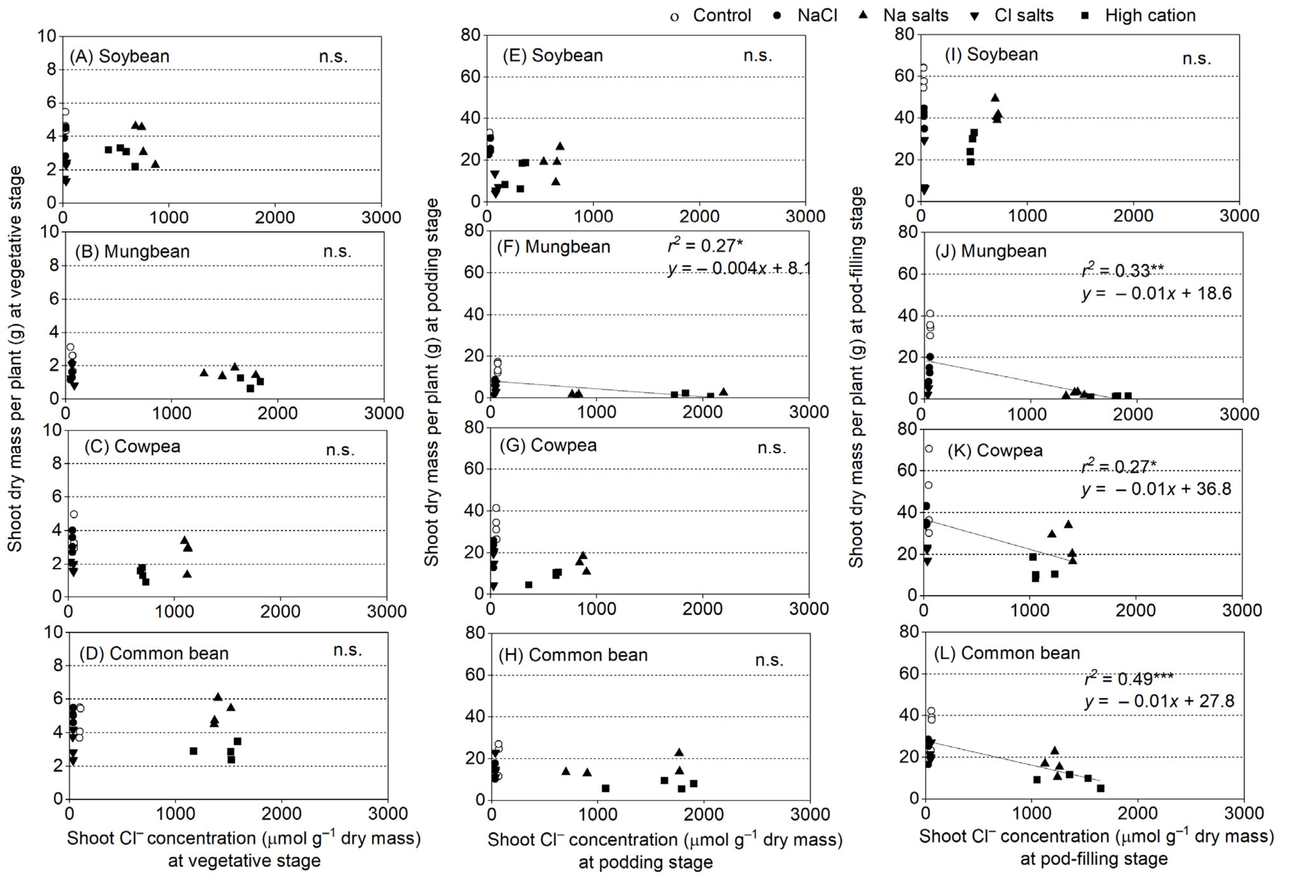

Figure S13. Scatter plots of shoot dry mass against shoot Cl^- concentration in soybean, mungbean, cowpea, and common bean grown in control (non-saline; open circles), 100 mM NaCl (solid circles), 100 mM Na^+ (without Cl^-) (solid, upward triangles), 100 mM Cl^- (without Na^+) (solid, downward triangles) and high cation negative control (solid squares) (K^+, Mg^{2+} and Ca^{2+} equivalent to those in the 100 mM Cl^- treatment) treatments. Salts used in the various treatments are given in Table 1. Treatments were imposed on 13 day-old plants and sampled after (**A**–**D**) 15 (vegetative stage), (**E**–**H**) 36 (podding stage) and (**I**–**L**) 57 (pod-filling stage) days of treatment. Each value is an individual replicate and each replicate is one plant growing in a different pot. * significant at $p < 0.05$, ** significant at $p < 0.01$, *** significant at $p < 0.001$, and n.s. = not significant. Note: the axis scales differ for (**A**–**D**) vegetative stage, (**E**–**H**) podding stage, and (**I**–**L**) reproductive stage.

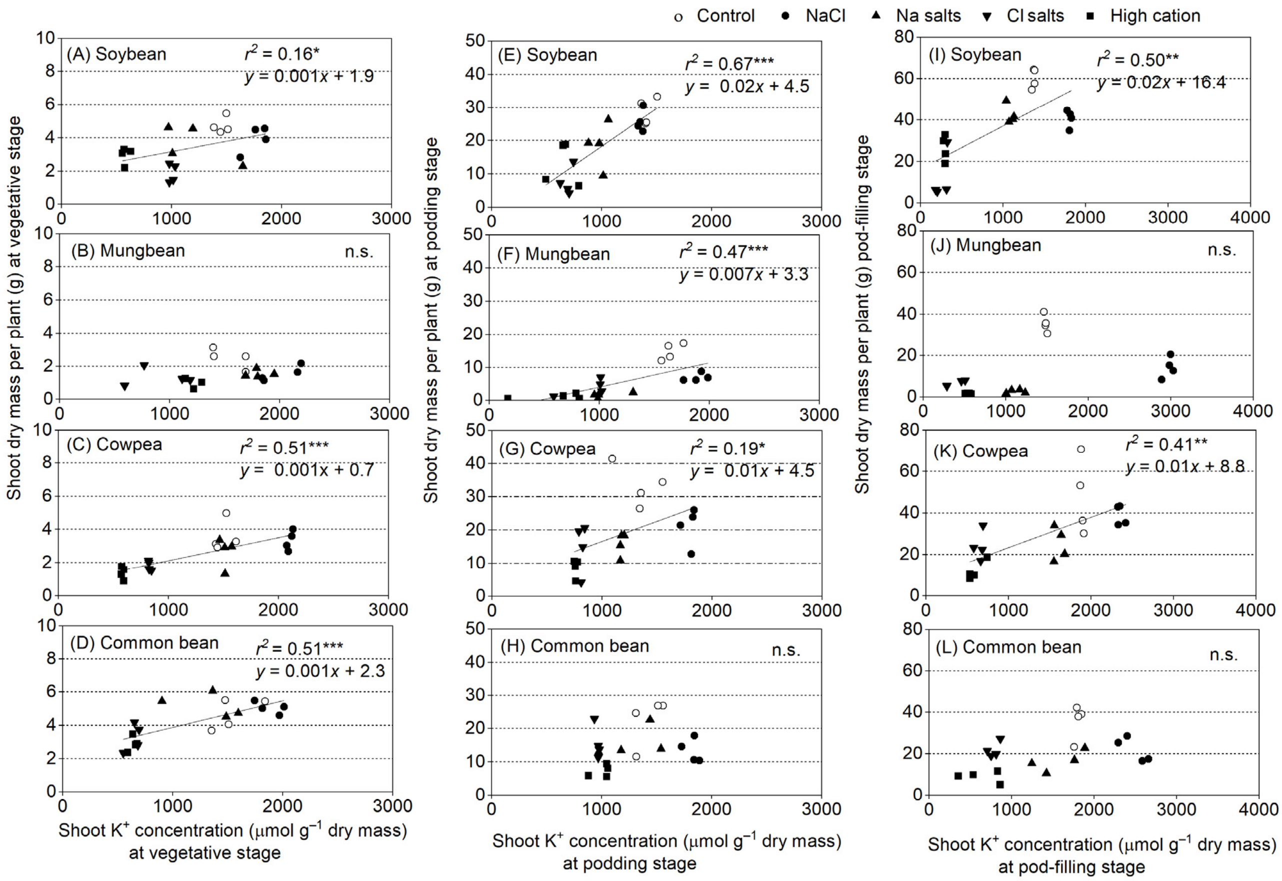

Figure S14. Scatter plots of shoot dry mass against shoot K^+ concentration of soybean, mungbean, cowpea, and common bean grown in control (non-saline; open circles), 100 mM NaCl (solid circles), 100 mM Na^+ (without Cl^-) (solid, upward triangles), 100 mM Cl^- (without Na^+) (solid, downward triangles) and high cation negative control (solid squares) (K^+, Mg^{2+} and Ca^{2+} equivalent to those in the 100 mM Cl^- salts) treatments. Salts used in the various treatments are given in Table 1. Treatments were imposed on 13 day-old plants and sampled after (**A**–**D**) 15 (vegetative stage), (**E**–**H**) 36 (podding stage) and (**I**–**L**) 57 (pod-filling stage) days of treatment. Each value is an individual replicate and each replicate is one plant growing in a different pot. * significant at $p < 0.05$, ** significant at $p < 0.01$, *** significant at $p < 0.001$, and n.s. = not significant. Note: the axis scales differ for (**A**–**C**) vegetative stage, (**D**–**F**) podding stage, and (**G**–**I**) reproductive stage.

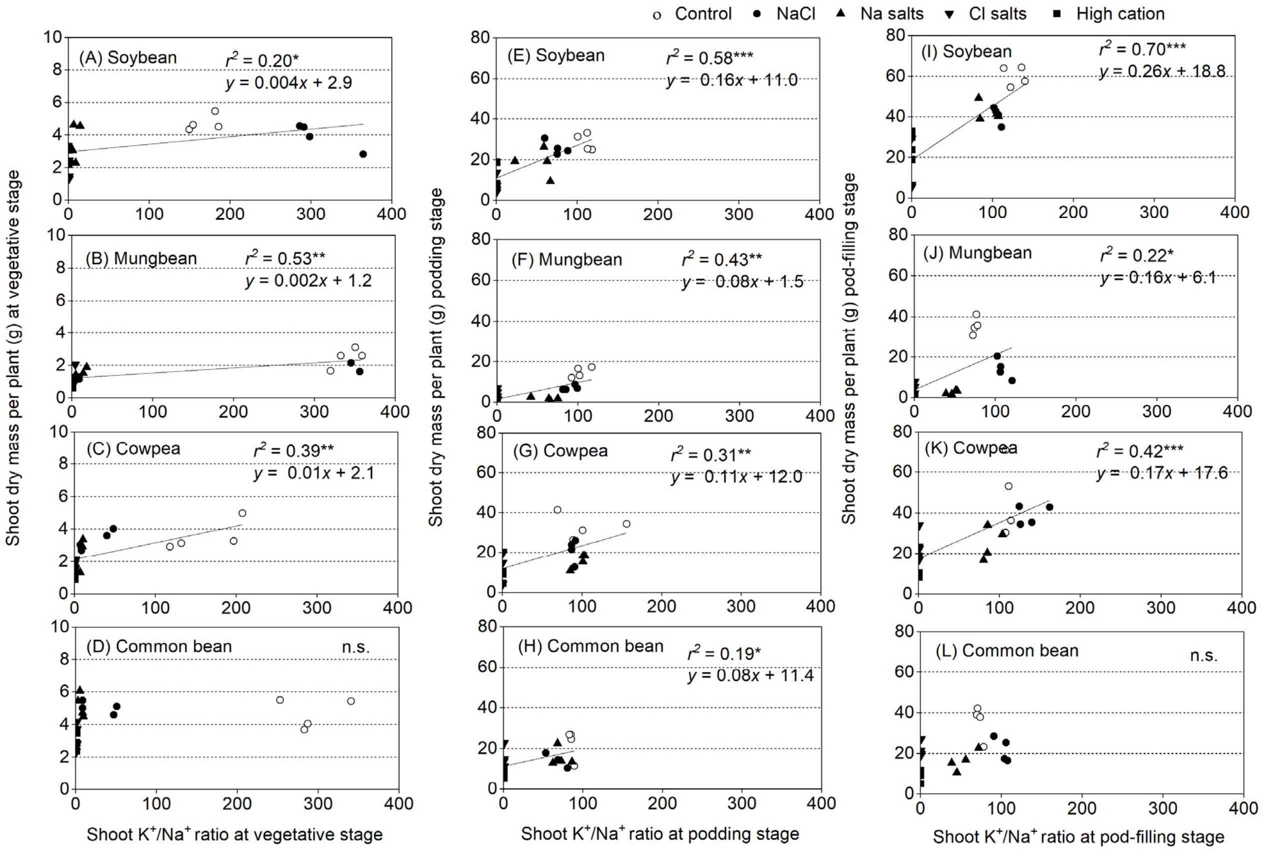

Figure S15. Scatter plots of shoot dry mass against shoot K^+/Na^+ ratio of soybean, mungbean, cowpea, and common bean grown in control (non-saline; open circles), 100 mM NaCl (solid circles), 100 mM Na^+ (without Cl^-) (solid, upward triangles), 100 mM Cl^- (without Na^+) (solid, downward triangles) and high cation negative control (solid squares) (K^+, Mg^{2+} and Ca^{2+} equivalent to those in the 100 mM Cl^- treatment) treatments. Salts used in the various treatments are given in Table 1. Treatments were imposed on 13 day-old plants and sampled after (**A**–**D**) 15 (vegetative stage), (**E**–**H**) 36 (podding stage) and (**I**–**L**) 57 (pod–filling stage) days of treatment. Each value is an individual replicate and each replicate is one plant growing in a different pot. * significant at $p < 0.05$, ** significant at $p < 0.01$, *** significant at $p < 0.001$, and n.s. = not significant. Note: the axis scale for vegetative stage (**A**–**C**) differs from the others.

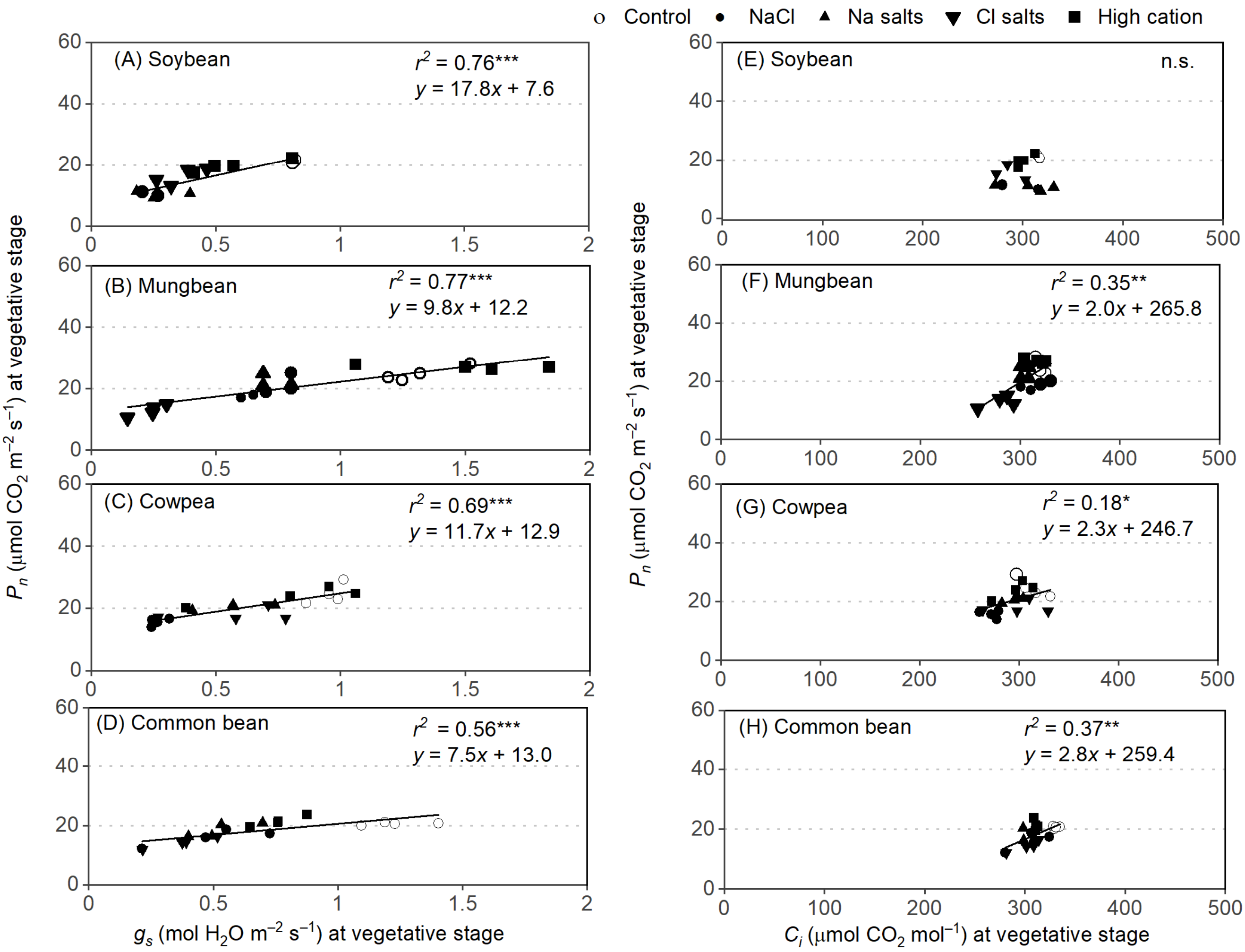

Figure S16. Scatter plots of net photosynthesis (P_n) against (**A–D**) stomatal conductance (g_s) and (**E–H**) intercellular CO_2 concentration (C_i) measured on the second fully expanded leaf after 13 and 14 days of treatment in soybean, mungbean, cowpea, and common bean grown in control (non-saline; open circles), 100 mM NaCl (solid circles), 100 mM Na^+ (without Cl^-) (solid, upward triangles), 100 mM Cl^- (without Na^+) (solid, down triangles) and high cation negative control (solid squares) (K^+, Mg^{2+} and Ca^{2+} equivalent to those in the 100 mM Cl^- treatment) treatments. Salts used in the various treatments are given in Table 1. Treatments were imposed on 13 day-old plants with gas exchange measured after 13–14 (vegetative stage), 33–35 (podding stage), 53–55 (pod-filling) days of treatment between 09:00 and 15:00 at photosynthetically active radiation of 1500 μmol photons m^{-2} s^{-1}, CO_2 concentration of 400 μmol mol^{-1}, 28° C leaf chamber temperature and 60–70% relative humidity. Each value is an individual replicate and each replicate is one plant growing in a different pot. * significant at $p < 0.05$, ** significant at $p < 0.01$, *** significant at $p < 0.001$, and n.s. = not significant.

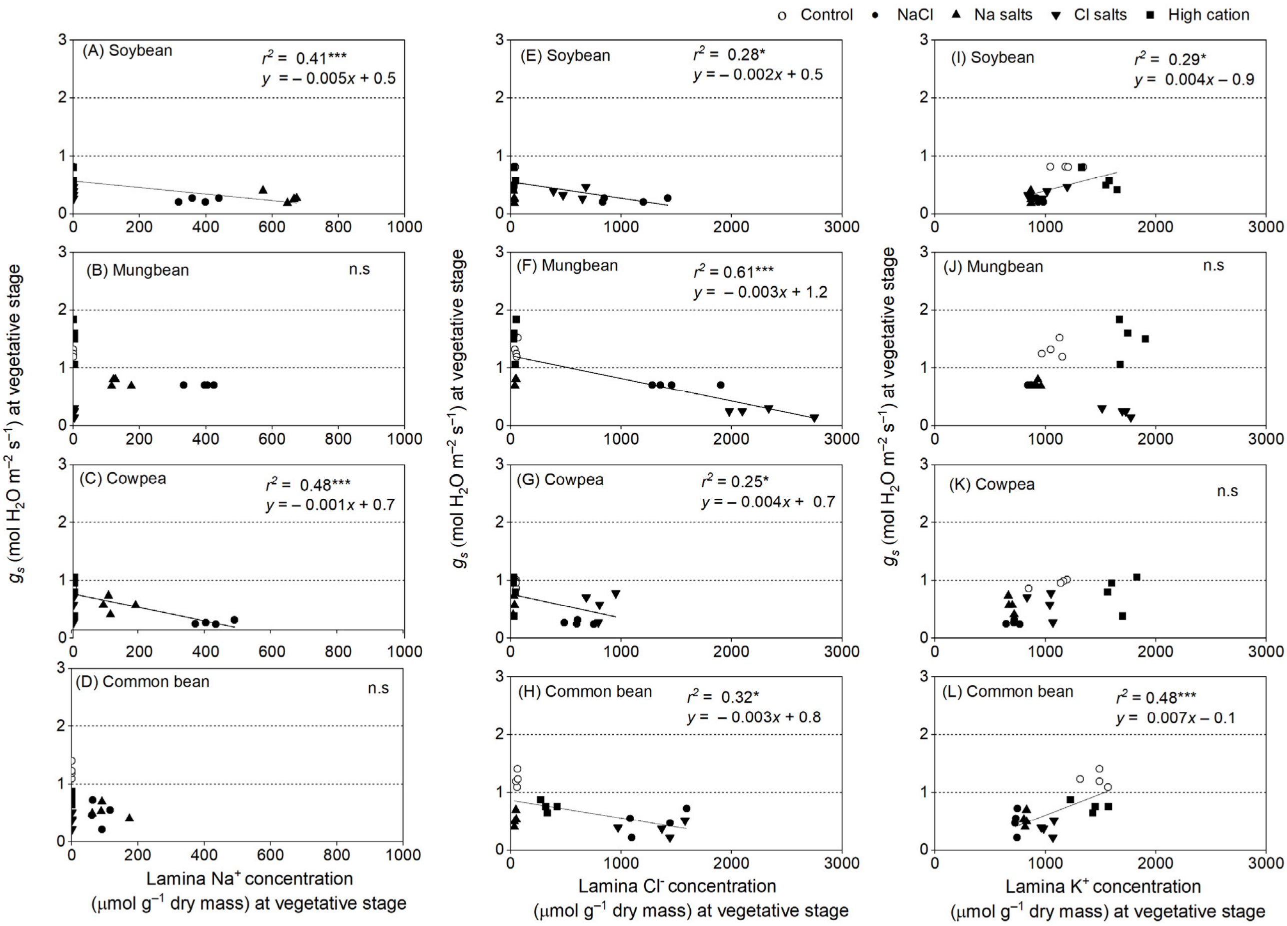

Figure S17. Scatter plots of stomatal conductance (g_s) against (**A**–**D**) lamina Na^+ concentration, (**E**–**H**) lamina Cl^- concentration, (**I**–**L**) lamina K^+ concentration of soybean, mungbean, cowpea, and common bean grown in control (non-saline; open circles), 100 mM NaCl (solid circles), 100 mM Na^+ (without Cl^-) (solid, upward triangles), 100 mM Cl^- (without Na^+) (solid, downward triangles) and high cation negative control (solid squares) (K^+, Mg^{2+} and Ca^{2+} equivalent to those in the 100 mM Cl^-) treatments. Salts used in the various treatments are given in Table 1. Treatments were imposed on 13 day-old plants with gas exchange measured after 13–14 (vegetative stage) days of treatment between 09:00 and to 15:00 at photosynthetically active radiation of 1500 μmol photons m^{-2} s^{-1}, CO_2 concentration of 400 μmol mol^{-1}, 28 °C leaf chamber temperature, and 60–70% relative humidity. Each value is an individual replicate and each replicate is one plant.

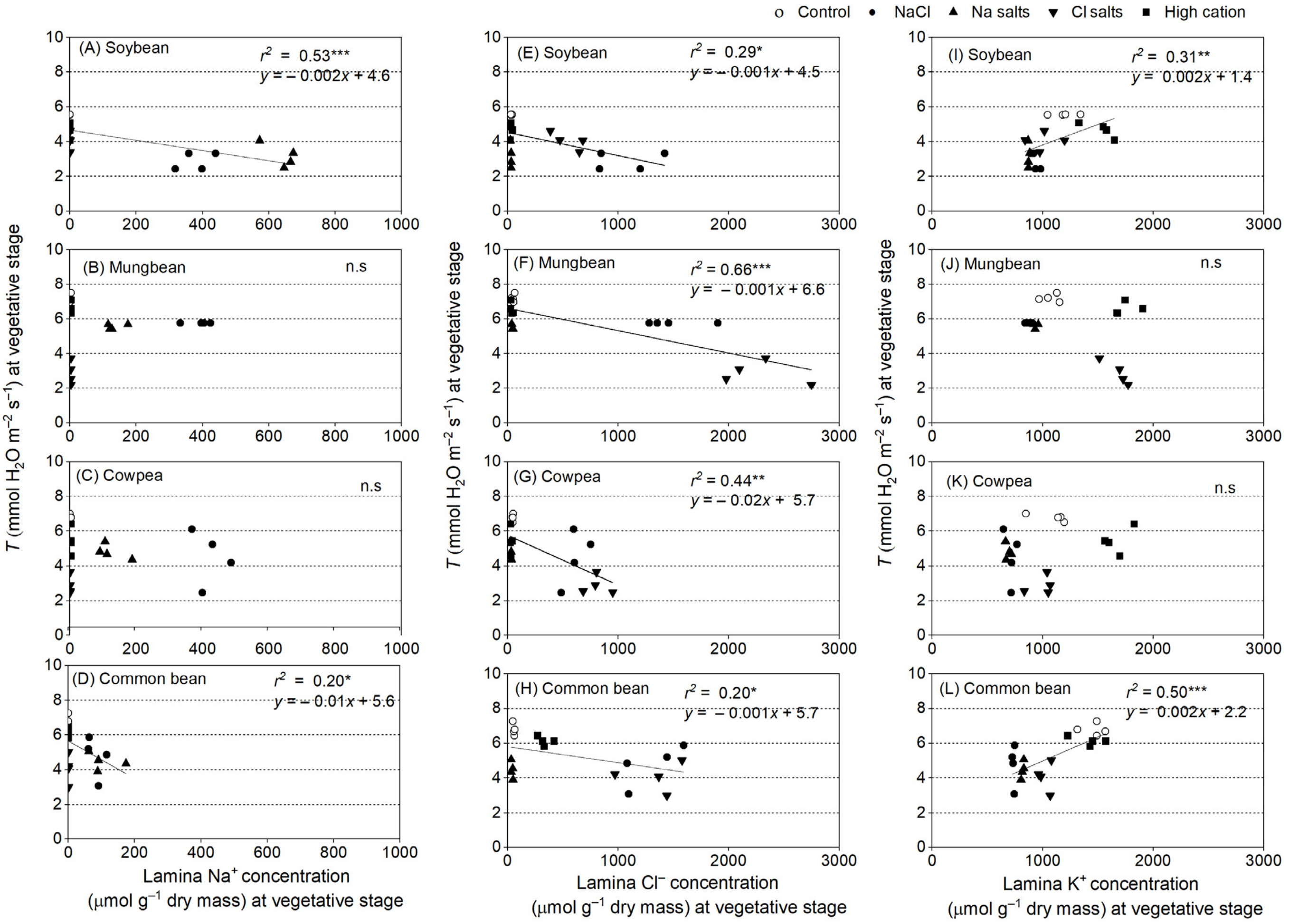

Figure S18. Scatter plots of transpiration rate (*T*) against (**A**–**D**) lamina Na^+ concentration, (**E**–**H**) lamina Cl^- concentration, (I, J, K, L) lamina K^+ concentration of soybean, mungbean, cowpea, and common bean grown in control (non-saline; open circles), 100 mM NaCl (solid circles), 100 mM Na^+ (without Cl^-) (solid, upward triangles), 100 mM Cl^- (without Na^+) (solid, downward triangles) and high cation negative control (solid squares) (K^+, Mg^{2+} and Ca^{2+} equivalent to those in the 100 mM Cl^-) treatments. Salts used in the various treatments are given in Table 1. Treatments were imposed on 13 day-old plants with gas exchange measured after 13–14 (vegetative stage) days of treatment between 09:00 and to 15:00 at photosynthetically active radiation of 1500 μmol photons m^{-2} s^{-1}, CO_2 concentration of 400 μmol mol^{-1}, 28°C leaf chamber temperature, and 60–70% relative humidity. Each value is an individual replicate and each replicate is one plant.

Table S1. Days to first leaf damage (brown and desiccated tissue and 10% of the lamina damaged on a leaf) and days to first flower in soybean, mungbean, cowpea, and common bean grown in control (non–saline), 100 mM NaCl, 100 mM Na^+ (without Cl^-), 100 mM Cl^- (without Na^+) and high cation negative control (K^+, Mg^{2+} and Ca^{2+} equivalent to those in the 100 mM Cl^-) treatments. Salts used in the various treatments are given in Table 1. Treatments were imposed on 13 day-old plants. The number of days is since the imposition of treatments. Values are means ± SE (n = 4). Least significant differences (LSD) for treatment means within each species, treatment, and species × treatment interaction are given at the bottom of each data column (p = 0.05). The probability levels for two-way ANOVA were used to compare species (S), treatment (T) and species × treatment interaction ($S \times T$) effects (*** $p < 0.001$ and n.s. = not significant).

Species	Treatment	Days to First Leaf Damage since Treatments commenced	Days to First Flower since Treatments Commenced
Soybean	Control	CN.A	25 ± 0.5
	NaCl	15 ± 0.5	23 ± 0.4
	Na^+ salts	13 ± 0.5	27 ± 0.5
	Cl^- salts	15 ± 0.5	23 ± 0.4
	High cation	N.A	23 ± 0.4
	LSD (5%)	1.5 ***	1.3 ***
Mungbean	Control	N.A	27 ± 0.6
	NaCl	8 ± 0.4	29 ± 0.5
	Na^+ salts	12 ± 0.8	28 ± 0.3
	Cl^- salts	10 ± 0.5	32 ± 0.5
	High cation	16 ± 0.5	21 ± 0.5
	LSD (5%)	1.3 ***	1.4 ***
Cowpea	Control	N.A	27 ± 0.4
	NaCl	14 ± 0.3	28 ± 0.5
	Na^+ salts	13 ± 0.5	32 ± 0.5
	Cl^- salts	14 ± 0.3	30 ± 0.3
	High cation	18 ± 0.8	26 ± 0.5
	LSD (5%)	0.8 ***	1.3 ***
Common bean	Control	N.A	29 ± 0.3
	NaCl	13 ± 0.8	29 ± 0.5
	Na^+ salts	11 ± 0.3	26 ± 0.5
	Cl^- salts	11 ± 1.0	25 ± 0.3
	High cation	16 ± 0.5	24 ± 0.5
	LSD (5%)	1.7 ***	1.2 ***
LSD (5%)	S	0.9 ***	0.5 ***
	T	n.s.	0.6 ***
	$S \times T$	1.5 ***	1.2 ***

Table S2. Tissue ion (Na^+, Cl^-, K^+, and K^+/Na^+) in lamina, petiole, stems and roots of soybean, mungbean, cowpea and common bean grown in control (non-saline). Ion concentrations and osmotic potential in a complete basal (non-saline control) nutrient solution are given in Table 1. Plants sampled at the imposition of treatments on 13 day-old plants. Values are means ± SE (*n* = 4). Least significant differences (LSD) for treatment means within each species, treatment, and species × treatment interaction are given at the bottom of each data column (*p* = 0.05). The probability levels for two-way ANOVA were used to compare specie (*S*), treatment (*T*), and species × treatment interaction (*S* × *T*) effects (*** $p < 0.001$ and n.s. = not significant).

Tissue	Species	Na^+ (µmol g^{-1} Dry Mass)	K^+ (µmol g^{-1} Dry Mass)	Cl^- (µmol g^{-1} Dry Mass)	K^+/Na^+ Ratio
Lamina	Soybean	2.6 ± 0.1	1151 ± 63	19.9 ± 0.5	431 ± 1.3
	Mungbean	2.6 ± 0.03	1268 ± 182	44.0 ± 0.5	478 ± 7.3
	Cowpea	2.5 ± 0.1	1115 ± 19	35.8 ± 0.5	436 ± 1.4
	Common bean	2.5 ± 0.04	1133 ± 41	41.2 ± 0.3	441 ± 1.5
	LSD (5%)	n.s	n.s	***	n.s
Petioles	Soybean	2.9 ± 0.4	2117 ± 13	43.9 ± 1.4	724 ± 13.0
	Mungbean	2.5 ± 0.1	681 ± 3	42.5 ± 4.1	624 ± 2.1
	Cowpea	6.8 ± 0.4	5604 ± 92	177.3 ± 2.8	82.7 ± 3.5
	Common bean	5.8 ± 0.4	2187 ± 32	47.8 ± 0.1	37.1 ± 0.8
	LSD (5%)	***	n.s	***	***
Stems	Soybean	2.6 ± 0.03	1416 ± 21	36. 3 ± 0.7	52.5 ± 0.5
	Mungbean	4.1 ± 0.8	1669 ± 10	120.3 ± 1.9	43.8 ± 6.4
	Cowpea	2.7 ± 0.1	1685 ± 94	37.0 ± 0.3	61.0 ± 0.5
	Common bean	2.5 ± 0.07	1519 ± 61	79.1 ± 1.5	59.9 ± 0.6
	LSD (5%)	n.s	n.s	***	n.s
Roots	Soybean	5.1 ± 0.1	1374 ± 38	36.2 ± 0.9	26.8 ± 0.5
	Mungbean	5.3 ± 0.07	2134 ± 25	120.3 ± 0.4	39.8 ± 0.8
	Cowpea	5.3 ± 0.1	1797 ± 43	37.0 ± 0.4	33.8 ± 0.3
	Common bean	5.5 ± 0.03	1235 ± 14	79.1 ± 1.5	22.4 ± 0.2
	LSD (5%)	n.s	***	***	n.s

Table S3. K^+/Na^+ in lamina, petiole, stems and roots of soybean, mungbean, cowpea and common bean grown in NaCl and Na^+ salts (without Cl^-). Ion concentrations and osmotic potential in a complete basal (non-saline control) nutrient solution are given in Table 1. Treatments were imposed on 13 day-old plants and sampled after15 (vegetative stage), 36 (podding stage) and 57 (pod-filling stage) days of treatment. Values are means ± SE (n = 4). There were no significant differences between these two treatments within each species.

Tissue	Species	Vegetative Stage		Podding Stage		Pod-Filling Stage	
		K^+/Na^+ Ratio in NaCl	K^+/Na^+ Ratio in Na^+	K^+/Na^+ Ratio in NaCl	K^+/Na^+ Ratio in Na^+	K^+/Na^+ Ratio in NaCl	K^+/Na^+ Ratio in Na^+
**Lamina**	Soybean	1.1 ± 0.1	1.9 ± 0.1	1.2 ± 0.1	1.2 ± 0.1	0.5 ± 0.01	0.5 ± 0.02
	Mungbean	4.7 ± 0.4	7.7 ± 0.5	1.4 ± 0.5	1.3 ± 0.2	1.4 ± 0.01	1.2 ± 0.01
	Cowpea	3.0 ± 0.1	1.5 ± 0.1	1.4 ± 0.2	0.9 ± 0.03	0.7 ± 0.2	0.7 ± 0.02
	Common bean	4.1 ± 1.0	4.7 ± 0.4	2.6 ± 0.2	1.8 ± 0.1	-	1.4 ± 0.1
Petioles	Soybean	1.8 ± 0.1	1.6 ± 0.01	1.4 ± 0.2	0.9 ± 0.03	0.3 ± 0.01	0.3 ± 0.01
	Mungbean	2.3 ± 0.01	5.8 ± 0.01	0.9 ± 0.04	1.0 ± 0.05	0.2 ± 0.01	0.6 ± 0.01
	Cowpea	1.0 ± 0.1	1.2 ± 0.2	1.8 ± 0.3	2.3 ± 0.1	0.7 ± 0.01	1.0 ± 0.2
	Common bean	3.5 ± 0.2	5.4 ± 0.03	1.8 ± 0.3	2.3 ± 0.1	1.9 ± 0.3	1.4 ± 0.03
Stems	Soybean	0.4 ± 0.02	0.5 ± 0.01	0.6 ± 0.1	0.4 ± 0.01	0.3 ± 0.01	0.1 ± 0.01
	Mungbean	0.7 ± 0.02	2.1 ± 0.1	0.3 ± 0.01	0.7 ± 0.01	0.2 ± 0.01	0.3 ± 0.02
	Cowpea	0.5 ± 0.01	1.1 ± 0.01	0.6 ± 0.01	0.6 ± 0.02	0.4 ± 0.01	0.5 ± 0.01
	Common bean	0.8 ± 0.02	1.0 ± 0.01	0.7 ± 0.02	0.7 ± 0.03	0.3 ± 0.02	0.7 ± 0.01
Shoots	Soybean	1.2 ± 0.1	1.1 ± 0.1	0.6 ± 0.1	0.5 ± 0.01	0.4 ± 0.1	0.6 ± 0.01
	Mungbean	1.5 ± 0.4	2.3 ± 0.4	0.5 ± 0.1	0.2 ± 0.01	0.3 ± 0.01	0.2 ± 0.01
	Cowpea	0.9 ± 0.01	1.2 ± 0.01	0.7 ± 0.01	0.7 ± 0.01	0.5 ± 0.1	0.4 ± 0.01
	Common bean	2.0 ± 0.1	2.7 ± 0.2	0.5 ± 0.03	0.2 ± 0.01	0.3 ± 0.1	0.2 ± 0.01
Roots	Soybean	0.8 ± 0.01	0.5 ± 0.01	0.5 ± 0.02	0.3 ± 0.01	0.2 ± 0.1	0.3 ± 0.03
	Mungbean	0.6 ± 0.01	0.4 ± 0.01	0.8 ± 0.03	0.1 ± 0.03	0.3 ± 0.04	0.1 ± 0.01
	Cowpea	1.2 ± 0.01	1.2 ± 0.1	0.7 ± 0.01	0.7 ± 0.02	0.6 ± 0.02	0.4 ± 0.03
	Common bean	0.9 ± 0.1	0.9 ± 0.01	0.5 ± 0.02	0.2 ± 0.03	0.9 ± 0.02	0.1 ± 0.01

Table S4. K^+ concentration and K^+/Na^+ ratio in flowers, pod walls, and seeds of soybean, mungbean, cowpea and common bean grown in control (non-saline). Ion concentrations and osmotic potential in a complete basal (non–saline control) nutrient solution are given in Table 1. Plants sampled after 57 days of treatment. Values are means ± SE (n = 4). Least significant differences (LSD) for treatment means within each species, treatment and species × treatment interaction are given at the bottom of each data column ($p = 0.05$). The probability levels for two-way ANOVA were used to compare specie (S), treatment (*T*) and species × treatment interaction (*S* × *T*) effects (* $p < 0.05$, *** $p < 0.001$, and n.s. = not significant).

Species	Treatment	Flowers		Pod walls		Seeds	
		K^+	K^+/Na^+	K^+	K^+/Na^+	K^+	K^+/Na^+
Soybean	Control	1119.4 ± 26.5	149.3 ± 11.8	1658.5 ± 23.5	333.3 ± 13.6	1073.3 ± 13.4	418.1 ± 14.9
	NaCl	1069.2 ± 12.3	8.2 ± 0.2	745.9 ± 22.9	3.1 ± 0.6	567.7 ± 10.9	5.5 ± 2.4
	Na^+ salts	1157.1 ± 25.0	2.8 ± 0.5	832.3 ± 15.8	1.4 ± 0.9	580.4 ± 17.6	2.1 ± 1.4
	Cl^- salts	1091.6 ± 15.4	151.9 ± 11.5	1447.6 ± 19.1	187.1 ± 13.4	1543.3 ± 19.3	278.7 ± 14.2
	High cation	738.9 ± 21.9	95.0 ± 13.1	1527.3 ± 19.5	112.8 ± 15.4	1638.9 ± 12.1	428.2 ± 15.0
	LSD (5%)	n.s.	45.6 ***	115 ***	124.5 ***	34.4 ***	128 *
Mung bean	Control	1231.6 ± 16.6	136.0 ± 10.2	1762.8 ± 17.5	310.4 ± 15.1	849.2 ± 15.1	306.1 ± 14.2
	NaCl	880.0 ± 15.1	5.5 ± 0.3	-	-	-	-
	Na^+ salts	840.6 ± 16.5	11.4 ± 0.9	862.5 ± 15.5	4.3 ± 2.3	557.8 ± 11.9	9.9 ± 0.4
	Cl^- salts	1682.4 ± 24.0	246.8 ± 12.3	797.6 ± 12.0	112.2 ± 23.1	122.2 ± 12.0	111.7 ± 11.5
	High cation	1434.7 ± 28.8	199.4 ± 16.8	2055.5 ± 25.8	253.6 ± 13.1	613.7 ± 11.1	195.7 ± 15.4
	LSD (5%)	240.9 ***	44.0 ***	73.9 ***	212.9 ***	40.1 ***	121 ***
Cowpea	Control	974.0 ± 11.3	138.9 ± 12.6	1161.5 ± 20.6	103.2 ± 15.1	629.0 ± 14.0	116.6 ± 18.8
	NaCl	1065.9 ± 24.1	11.3 ± 1.2	679.5 ± 15.0	1.6 ± 0.2	424.0 ± 14.5	6.5 ± 0.5
	Na^+ salts	1015.4 ± 22.5	8.5 ± 0.9	557.7 ± 17.3	1.0 ± 0.1	363.0 ± 10.2	3.2 ± 0.5
	Cl^- salts	1030.2 ± 31.6	146.8 ± 15.3	927.9 ± 85.0	112.1 ± 13.8	742.4 ± 16.9	227.6 ± 13.2
	High cation	1180.0 ± 29.5	145.2 ± 10.1	1123.8 ± 24.8	134.4 ± 11.4	737.6 ± 13.6	217.2 ± 14.8
	LSD (5%)	63.8 ***	15.7 ***	145.3 ***	65. 0 ***	34.3 ***	191.3 *
Common bean	Control	1571.1 ± 20.9	124.2 ± 11.3	1459.6±15.0	125.2±13.0	1053.4±15.4	381.3 ± 13.2
	NaCl	1034.0 ± 14.7	17.5 ± 3.2	756.0±78.3	1.1±0.2	642.3±24.6	2.9 ± 0.4
	Na^+ salts	1083.7 ± 29.9	15.2 ± 1.4	839.3±14.8	3.8±0.6	461.3±7.6	21.5 ± 1.0
	Cl^- salts	1503.5 ± 15.7	214.8 ± 15.8	1542.6±22.0	93.4±11.8	1425.0±19.3	142.8 ± 13.3
	High cation	1574.7 ± 64.8	163.3 ± 17.6	1576.3±54.3	153.1±13.2	1557.4±19.7	338.4 ± 13.6
	LSD (5%)	48.6 ***	25.6 ***	270.3 ***	65.2 ***	35.3 ***	304.4 *
LSD (5%)	*S*	73.9 ***	14.0 ***	73.4 **	37.6 ***	15.1 ***	115 *
	T	81.4 ***	15.4 ***	84.9 ***	45.4 ***	17.0 ***	131 ***
	S × *T*	165.5 ***	31.4 ***	161.7 ***	84.4 ***	33.1 ***	n.s.

Table S5. Leaf water content measured on the second youngest fully expanded leaves (leaf lamina) of soybean, mungbean, cowpea, and common bean, grown in control (non-saline), 100 mM NaCl, 100 mM Na^+ (without Cl^-), 100 mM Cl^- (without Na^+), and high cation negative control (K^+, Mg^{2+} and Ca^{2+} equivalent to those in the 100 mM Cl^- treatment) treatment. Salts used in the various treatments are given in Table 1. Treatments were imposed on 13 day-old plants and sampled after 15 (vegetative stage), 36 (podding stage), and 57 (pod-filling stage) days of treatment. Values are means ± SE (n = 4). The least significant differences (LSD) for treatment means within each species, treatments, and species × treatment interaction are given at the bottom of each data column (p = 0.05). The probability levels for two-way ANOVA were used to compare species (S), treatment (T) and species × treatment interaction ($S \times T$) effects (* $p < 0.05$, ** $p < 0.01$, *** $p < 0.001$, and n.s. = not significant).

Species	Treatment	Leaf Water Content (mL g^{-1} Dry Mass)		
		Vegetative	Podding	Pod-Filling
Soybean	Control	4.1 ± 0.2	2.6 ± 0.3	2.5 ± 0.3
	NaCl	4.5 ± 0.3	2.7 ± 0.1	2.4 ± 0.3
	Na^+ salts	4.8 ± 0.4	2.5 ±0.3	2.6 ± 0.1
	Cl^- salts	4.3 ± 0.2	3.7 ±0.4	3.0 ± 0.2
	High cation	4.3 ± 0.2	2.9 ±0.3	2.9 ± 0.1
	LSD (5%)	n.s.	n.s.	n.s.
Mungbean	Control	5.4 ± 0.4	4.0 ± 0.3	3.8 ± 0.3
	NaCl	5.1 ± 1.5	6.6 ± 0.0	–
	Na^+ salts	4.3 ± 0.3	3.3 ± 0.2	3.3 ± 0.1
	Cl^- salts	5.9 ± 0.3	6.4 ± 0.0	–
	High cation	4.6 ± 0.5	4.8 ± 0.4	4.7 ± 0.4
	LSD (5%)	n.s.	1.5 **	0.9 *
Cowpea	Control	4.9 ± 0.8	3.1 ± 0.1	4.8 ± 0.2
	NaCl	5.4 ± 0.3	5.1 ± 0.3	7.4 ± 0.6
	Na^+ salts	4.8 ± 0.2	3.9 ± 0.4	4.2 ± 0.3
	Cl^- salts	5.9 ± 0.5	5.4 ± 0.2	7.9 ± 0.7
	High cation	4.4 ± 0.7	4.2 ± 0.4	5.9 ± 0.3
	LSD (5%)	n.s.	0.9 ***	1.5 ***
Common bean	Control	5.6 ± 0.6	3.9 ± 0.7	4.8 ± 0.4
	NaCl	5.4 ± 0.6	5.9 ± 0.8	7.7 ± 0.0
	Na^+ salts	3.7 ± 0.2	4.3 ± 0.3	3.5 ± 0.2
	Cl^- salts	6.2 ± 0.0	6.9 ± 0.2	8.6 ± 0.0
	High cation	4.9 ± 0.3	3.7 ± 1.0	6.0 ± 0.4
	LSD (5%)	1.3 **	2.2 *	1.3 ***
LSD (5%)	*S*	n.s.	0.6 ***	0.5 ***
	T	0.7 ***	0.7 ***	0.6 ***
	S x T	n.s.	n.s.	1.1 ***

Table S6. Osmotic potential of the external solution bathing the roots (Ψπsol) (MPa), measured in control (non-saline), 100 mM NaCl, 100 mM Na^+ (without Cl^-), 100 mM Cl^- (without Na^+), and high cation negative control (K^+, Mg^{2+} and Ca^{2+} equivalent to those in the 100 mM Cl^- treatment) treatment. Ψπsol was measured of 7 days old nutrient solution at the same time of measuring leaf water content (Table S5) and leaf sap osmotic potential ($\Psi\pi_{sap}$) (Table 3) after 15 (vegetative stage), 36 (podding stage), and 57 (pod-filling stage) days of treatment. The change in $\Psi\pi_{sap}$ = Control – Treatment. Values are means ± SE (n = 4). The least significant differences (LSD) for treatment means within each species, treatments, and species × treatment interaction are given at the bottom of each data column (p = 0.05). The probability levels for one-way ANOVA were used to compare treatment *** $p < 0.001$).

Treatment	Vegetative		Podding		Pod-Filling	
	Ψπsol (MPa)	Change in Ψπsol (MPa)	Ψπsol (MPa)	Change in Ψπsol (MPa)	Ψπsol (MPa)	Change in Ψπsol (MPa)
Control	−0.04 ± 0.005	-	−0.03 ± 0.004	-	−0.03 ± 0.005	-
NaCl	−0.49 ± 0.018	0.45	−0.46 ± 0.019	0.43	−0.45 ± 0.015	0.42
Na^+ salts	−0.38 ± 0.021	0.34	−0.37 ± 0.018	0.34	−0.38 ± 0.023	0.35
Cl^- salts	−0.39 ± 0.033	0.35	−0.35 ± 0.020	0.32	−0.36 ± 0.025	0.33
High cation	−0.30 ± 0.028	0.26	−0.30 ± 0.009	0.27	−0.30 ± 0.010	0.27
LSD (5%)	0.06 ***	-	0.04 ***	-	0.05 ***	-

Table S7. Summary of toxic, marginal levels, and adequate levels of ion concentration for soybean, mungbean, cowpea and common bean.

Legume Species	Toxic Level	Sources
Soybean	Leaf Na^+ concentration: >0.5% by dry mass or 217 μmol g^{-1} dry mass (toxic)<br>Leaf Cl^- concentration: <2.6–5.0% by dry mass or 713–1407 μmol g^{-1} dry mass (toxic)<br>Leaf Ca^{2+} concentration: 2.5–3.0% by dry mass or 623–748 μmol g^{-1} dry mass (high – non–toxic); 0.21–0.35% by dry mass or 50–100 μmol g^{-1} dry mass (marginal)<br>Leaf K^+ concentration: 4.0% by dry mass or 1023 μmol g^{-1} dry mass (high – non–toxic); 1.0–1.5% by dry mass or 255–383 μmol g^{-1} dry mass (marginal); <0.8% by dry mass or < 204 μmol g^{-1} dry mass (deficiency)	(Weir 1994)
Cowpea	Leaf Na^+ concentration: 0.01– 0.03% by dry mass or 4.3–13 μmol g^{-1} dry mass (adequate – non–toxic)<br>Leaf Cl^- concentration: 0.7–1.6% by dry mass or 197–450 μmol g^{-1} dry mass (adequate – non–toxic), 1.9% by dry mass or 534 μmol g^{-1} dry mass (high – non–toxic)<br>Leaf K^+ concentration: 1.7–3.0% by dry mass or 434–767 μmol g^{-1} dry mass (adequate – non–toxic), 1.1–1.2% by dry mass or 281–306 μmol g^{-1} dry mass (marginal), 0.5–0.9% by dry mass or 127–230 μmol g^{-1} dry mass (deficiency)	(Weir 1994)
Mungbean	Shoot Cl^- concentrations: 1.18% or 332 μmol g^{-1} dry weight (toxic)<br>No information on Na^+ and K^+	(Reuter & Robinson 1997)
Common bean	Shoot Na^+ concentration: 0.7% by dry mass or 305 μmol g^{-1} dry mass (toxic)	(Awada et al. 1995)
	Shoot Na^+ concentration: 20–25 mmol kg^{-1} tissue water) (toxic)<br>Shoot Cl^- concentration: 30–40 mmol kg^{-1} tissue water) (toxic)	(Salim 1989)
	Leaf K^+ concentration: 1.3–1.5% by dry mass or 332–383 μmol g^{-1} dry mass (adequate–non toxic)	(Reuter & Robinson 1997)
